# Regularization Methods Based on the *L_q_*-Likelihood for Linear Models with Heavy-Tailed Errors

**DOI:** 10.3390/e22091036

**Published:** 2020-09-16

**Authors:** Yoshihiro Hirose

**Affiliations:** 1 Faculty of Information Science and Technology, Hokkaido University, Kita 14, Nishi 9, Kita-ku, Sapporo, Hokkaido 060-0814, Japan; hirose@ist.hokudai.ac.jp; 2 Global Station for Big Data and Cybersecurity, Global Institution for Collaborative Research and Education, Hokkaido University, Hokkaido 060-0814, Japan

**Keywords:** least absolute shrinkage and selection operator (LASSO), minimax concave penalty (MCP), power function, *q*-normal distribution, smoothly clipped absolute deviation (SCAD), sparse estimation

## Abstract

We propose regularization methods for linear models based on the $L_{q}$-likelihood, which is a generalization of the log-likelihood using a power function. Regularization methods are popular for the estimation in the normal linear model. However, heavy-tailed errors are also important in statistics and machine learning. We assume *q*-normal distributions as the errors in linear models. A *q*-normal distribution is heavy-tailed, which is defined using a power function, not the exponential function. We find that the proposed methods for linear models with *q*-normal errors coincide with the ordinary regularization methods that are applied to the normal linear model. The proposed methods can be computed using existing packages because they are penalized least squares methods. We examine the proposed methods using numerical experiments, showing that the methods perform well, even when the error is heavy-tailed. The numerical experiments also illustrate that our methods work well in model selection and generalization, especially when the error is slightly heavy-tailed.

## 1. Introduction

We propose regularization methods based on the $L_{q}$-likelihood for linear models with heavy-tailed errors. These methods turn out to coincide with the ordinary regularization methods that are used for the normal linear model. The proposed methods work efficiently, and can be computed using existing packages.

Linear models are widely applied, and many methods have been proposed for estimation, prediction, and other purposes. For example, for estimation and variable selection in the normal linear model, the literature on sparse estimation includes the least absolute shrinkage and selection operator (LASSO) [1], smoothly clipped absolute deviation (SCAD) [2], Dantzig selector [3], and minimax concave penalty (MCP) [4]. The LASSO has been studied extensively and generalized to many models, including the generalized linear models [5]. As is well known, the regularization methods have many good properties. Many regularization methods are the penalized maximum likelihood estimators, that is, minimizing the sum of the negative log-likelihood and a penalty. The literature proposed various penalties. As described later, our regularization methods use another likelihood with existing penalties.

Because the regularization methods for the normal linear model are useful, they are sometimes used in linear models with non-normal errors. Here, popular errors include the Cauchy error and the *t*-distribution error, both of which are heavy-tailed errors. For example, Ref. [6] partly consider the Cauchy and *t*-distribution errors in their extensive experiments. These heavy-tailed distributions are known to be *q*-normal distributions, which are studied in the literature on statistical mechanics [7,8,9]. The *q*-normal model is also studied in the literature on the generalized Cauchy distribution. For example, see [10,11,12,13].

In this study, we consider the problem of a linear regression with a *q*-normal error. We propose sparse estimation methods based on the $L_{q}$-likelihood, which is a generalization of the log-likelihood using a power function. The maximizer of the $L_{q}$-likelihood, the maximum $L_{q}$-likelihood estimator (ML*q*E), is investigated by [14] as an extension of the ordinary maximum likelihood estimator (MLE). Ref. [14] studies the asymptotic properties of the ML*q*E. However, we are interested in the regularization, not in the ML*q*E, because regularization estimators can be better than the ML*q*E. We examine the proposed methods using numerical experiments. The experiments show that our methods perform well in model selection and generalization, even when the error is heavy-tailed. Moreover, we consider the effects of the sample size, dimension and sparseness of the parameter, and value of the nonzero elements in the numerical experiments.

We also find that the proposed methods for linear models with *q*-normal errors coincide with the ordinary regularization methods that are applied to the normal linear model. This finding partly justifies the use of the ordinary regularization methods for linear regressions with heavy-tailed errors. Moreover, the proposed methods are penalized least squares methods, and can be efficiently computed by existing packages.

The rest of the paper is organized as follows. In Section 2, we introduce several tools, including the normal linear model, regularization methods, $L_{q}$-likelihood, and *q*-normal models. In Section 3, we describe the problem under consideration, that is, estimations in linear models with *q*-normal errors. Moreover, we propose several regularization methods based on the $L_{q}$-likelihood. In Section 4, we evaluate the proposed methods using numerical experiments. Section 5 concludes the paper.

## 2. Preliminaries

### 2.1. Normal Linear Model and Sparse Estimation

First, we introduce the normal linear model, the estimation of which is a basic problem in statistics and machine learning [15]. Furthermore, we briefly describe some well-known regularization methods.

The normal linear model is defined as follows. A response is represented by a linear combination of explanatory variables $x_{1},x_{2},\cdots ,x_{d}$ as

(1) $$y^{a}=\theta^{0}+\sum_{i=1}^{d}x_{i}^{a}\theta^{i}+\varepsilon^{a}\;\left(a=1,2,\cdots ,n\right),$$

where $y^{a}$ is the response of the *a*-th sample, *n* is the sample size, *d* is the number of explanatory variables, $x_{i}^{a}$ is the *i*-th explanatory variable of the *a*-th sample, $\varepsilon^{a}$ is a normal error with mean zero and known variance, and the regression coefficient $\theta ={\left(\theta^{0},\theta^{1},\cdots ,\theta^{d}\right)}^{⊤}$ is the parameter to be estimated. The normal linear model is equivalently given by

μ=Xθ,

where $\mu^{a}=E\left[y^{a}\right]$ is the expectation of the response $y^{a}$, $\mu =(\mu^{a})$, and $X=(x_{i}^{a})$ is a design matrix of size n×(d+1), with $x_{0}^{a}=1\;\left(a=1,2,\cdots ,n\right)$. Moreover, we define a row vector $x^{a}\;\left(a=1,2,\cdots ,n\right)$ as $x^{a}=\left(1,x_{1}^{a},x_{2}^{a},\cdots ,x_{d}^{a}\right)$, and a column vector $x_{i}\;\left(i=0,1,2,\cdots ,d\right)$ as $x_{i}={\left(x_{i}^{1},x_{i}^{2},\cdots ,x_{i}^{n}\right)}^{⊤}$, which results in $X={\left(x^{1⊤},x^{2⊤},\cdots ,x^{n⊤}\right)}^{⊤}=\left(x_{0},x_{1},x_{2},\cdots ,x_{d}\right)$. Let $y=(y^{a})$ be the response vector of length *n*. We assume that each column vector $x_{i}\;\left(i=1,2,\cdots ,d\right)$ is standardized, as follows: $\sum_{a=1}^{n}x_{i}^{a}=0$ and $∥x_{i}∥=1$, for i=1,2,⋯,d.

As is well known, some regularization methods for the normal linear model are formulated as an optimization problem in the form of

(2) $$\min_{\theta \in R^{d+1}}\left\{\frac{1}{2n}{∥y-X\theta ∥}^{2}+\rho_{\lambda }\left(\theta \right)\right\},$$

where $\rho_{\lambda }\left(\theta \right)$ is a penalty term, and λ≥0 is a regularization parameter. The LASSO [1] uses $\rho_{\lambda }\left(\theta \right)={\lambda ∥\theta ∥}_{1}=\lambda \sum_{i=1}^{d}\left|\theta^{i}\right|$. The path of the LASSO estimator when λ varies can be made by the least angle regression (LARS) algorithm [16]. The SCAD [2] uses

(3) $$\begin{array}{c} \rho_{\lambda }\left(\theta \right)=\left\{\begin{array}{c} \sum_{i=1}^{d}\lambda |\theta^{i}\left|\;(\right|\theta^{i}\left|\leq \lambda \right),\\ -\sum_{i=1}^{d}\frac{|\theta^{i}{|}^{2}-2a\lambda \left|\theta^{i}\right|+\lambda^{2}}{2(a-1)}\;(\lambda <|\theta^{i}\left|\leq a\lambda \right),\\ \sum_{i=1}^{d}\frac{\left(a+1\right)\lambda^{2}}{2}\;(a\lambda <|\theta^{i}\left|\right), \end{array}\right. \end{array}$$

and the MCP [4] uses

(4) $$\begin{array}{c} \rho_{\lambda }\left(\theta \right)=\lambda \sum_{i=1}^{d}\int_{0}^{|\theta^{i}|}{\left(1-\frac{u}{\gamma \lambda }\right)}_{+}du, \end{array}$$

where a(>2) and γ(>0) are tuning parameters.

The regularization problem given in (2) can be represented by

(5) $$\min_{\theta \in R^{d+1}}\left\{-\frac{1}{n}\log f\left(y|\theta \right)+\rho_{\lambda }\left(\theta \right)\right\},$$

where f(y|θ) is the probability density function of the statistical model. Note that logf(y|θ) is the log-likelihood.

### 2.2. $L_{q}$-Likelihood

The $L_{q}$-likelihood is a generalization of the log-likelihood that uses a power function instead of the logarithmic function. Let $y={\left(y^{1},y^{2},\cdots ,y^{n}\right)}^{⊤}$ be a vector of independent and identically distributed (i.i.d.) observations, and let θ be a parameter of a statistical model. For q>0(q≠1), the $L_{q}$-likelihood function is defined as

(6) $$\begin{array}{c} L_{q}\left(\theta |y\right)=\sum_{a=1}^{n}\log_{q}f\left(y^{a}|\theta \right), \end{array}$$

where f(·|θ) is a probability density function of the statistical model, and

$$\begin{array}{c} \log_{q}\left(u\right)=\frac{1}{1-q}\left(u^{1-q}-1\right)\;\left(u>0\right) \end{array}$$

is the *q*-logarithmic function [9]. For q=1, we define

$$\begin{array}{c} \log_{1}\left(u\right)=\log u\;\left(u>0\right), \end{array}$$

which is the ordinary logarithmic function. When q=1, the $L_{q}$-likelihood is the log-likelihood.

The ML*q*E is defined as the estimator that maximizes the $L_{q}$-likelihood. [14] studied the asymptotic performance of the ML*q*E, showing that it enjoys good asymptotic properties (e.g., asymptotic normality).

### 2.3. q-Normal Model

Before defining the *q*-normal distribution [7,8,9], we introduce the *q*-exponential function. For q>0(q≠1), the *q*-exponential function is the inverse function of the *q*-logarithmic function, and is given by

$$\begin{array}{c} \exp_{q}\left(u\right)={\left\{1+\left(1-q\right)u\right\}}^{\frac{1}{1-q}}\;\left(u<-1/\left(1-q\right)\right). \end{array}$$

For q=1, the 1-exponential function is the ordinary exponential function

$$\begin{array}{c} \exp_{1}\left(u\right)=\exp u\;\left(u\in R\right). \end{array}$$

Using the *q*-exponential function, the *q*-normal model is given by

$$\begin{array}{cc} S_{q} & =\{f_{q}\left(y|\;\xi ,\sigma \right)|\xi \in \Xi ,\sigma >0\},\\ f_{q}\left(y|\;\xi ,\sigma \right) & =\frac{1}{Z_{q}}\exp_{q}\left\{-\frac{1}{3-q}{\left(\frac{y-\xi }{\sigma }\right)}^{2}\right\}\\ \; & =\frac{1}{Z_{q}}{\left\{1-\frac{1-q}{3-q}{\left(\frac{y-\xi }{\sigma }\right)}^{2}\right\}}^{\frac{1}{1-q}}, \end{array}$$

where ξ is a location parameter, Ξ⊂R is the parameter space, and σ is a dispersion parameter. The constant $Z_{q}$ is a normalizing constant.

We assume that 1≤q<3, which ensures that the sample space is the real line itself, not just part of it. Moreover, the parameter space is Ξ=R when 1≤q<3.

For example, the 1-normal model is the ordinary normal model. Another example is the Cauchy distribution for q=2:

$$\begin{array}{c} f_{2}\left(y|\mu ,\sigma \right)=\frac{1}{\sigma B(\frac{1}{2},\frac{1}{2})}{\left(1+\frac{{\left(y-\xi \right)}^{2}}{\sigma^{2}}\right)}^{-1}, \end{array}$$

where B(·,·) is the beta function. Furthermore, the *t*-distribution of the degree of freedom ν is obtained for q=1+2/(ν+1):

$$\begin{array}{c} f_{1+2/(\nu +1)}\left(y|\mu ,\sigma \right)=\frac{1}{\sqrt{\nu }\sigma B\left(\frac{\nu }{2},\frac{1}{2}\right)}{\left(1+\frac{{\left(y-\mu \right)}^{2}}{\nu \sigma^{2}}\right)}^{-\frac{\nu +1}{2}}. \end{array}$$

## 3. Problem and Estimation Method

### 3.1. Linear Model with *q*-Normal Error

In this subsection, we formulate our problem, that is, a linear regression with a heavy-tailed error. The errors of the Cauchy and *t*-distributions in linear models have been studied by researchers in the context of heavy-tailed errors [17,18,19,20]. However, they focused mainly on the least squares methods, whereas we are interested in sparse estimators. Moreover, our approach is based on the $L_{q}$-likelihood, not the ordinary log-likelihood.

We examine the problem of estimating the linear model given in (1) with i.i.d. errors from a *q*-normal distribution; henceforth, we refer to this as the *q*-normal linear model. In terms of probability distributions, we wish to estimate the parameter θ of the *q*-normal linear model $M_{q}$:    

(7) $$\begin{array}{cc} M_{q} & =\{f\left(\cdot |\;\theta \right)|\;\theta \in R^{d+1}\},\\ f(y|\;\theta ) & =\frac{1}{Z_{q}^{n}}\prod_{a=1}^{n}\exp_{q}\left\{-\frac{{\left(y^{a}-x^{a}\theta \right)}^{2}}{3-q}\right\}\\ \; & =\frac{1}{Z_{q}^{n}}\prod_{a=1}^{n}{\left\{1-\frac{1-q}{3-q}{\left(y^{a}-x^{a}\theta \right)}^{2}\right\}}^{\frac{1}{1-q}}, \end{array}$$

where the dispersion parameter is assumed to be known (σ=1). The 1-normal linear model is identical to the normal linear model, as described in Section 2.1.

### 3.2. $L_{q}$-Likelihood-Based Regularization Methods

We propose regularization methods based on the $L_{q}$-likelihood. For *q*-normal linear models, the proposed methods coincide with the original regularization methods for the normal linear model. In other words, we apply the ordinary regularization methods as if the error distribution were a normal distribution. The literature describes how to compute the proposed methods efficiently. Moreover, our method calculates the ML*q*E.

We define the $L_{q}$-likelihood for the *q*-normal linear model in (7) as (6), where θ is the regression coefficient. Note that the components of y are not assumed to be identically distributed because their distributions are dependent on the explanatory variables.

The $L_{q}$-likelihood for the *q*-normal linear model is

(8) $$\begin{array}{cc} L_{q}\left(\theta |y\right) & =\sum_{a=1}^{n}\log_{q}f\left(y^{a}|\theta \right)\\ \; & =\sum_{a=1}^{n}\log_{q}\left[\frac{1}{Z_{q}}\exp_{q}\left\{-\frac{{\left(y^{a}-x^{a}\theta \right)}^{2}}{3-q}\right\}\right]\\ \; & =-\frac{Z_{q}^{q-1}}{3-q}{∥y-X\theta ∥}^{2}-n\log_{q}\left(Z_{q}\right), \end{array}$$

where the second term is a constant. The ML*q*E of the parameter θ is defined as the maximizer of the $L_{q}$-likelihood. In the *q*-normal linear model, the ML*q*E is equal to the ordinary least square, the MLE for the normal linear model.

We propose a LASSO, SCAD, and MCP based on the $L_{q}$-likelihood by replacing the log-likelihood with the $L_{q}$-likelihood in the optimization problem in (5). That is, the $L_{q}$-likelihood-based regularization methods are given in the form of

(9) $$\min_{\theta \in R^{d+1}}\left\{-\frac{1}{n}L_{q}\left(\theta |y\right)+\rho_{\lambda }\left(\theta \right)\right\}.$$

The penalty $\rho_{\lambda }$ is ${\lambda ∥\theta ∥}_{1}$ for the LASSO, (3) for the SCAD, and (4) for the MCP. Note that the estimator for λ=0 is the ML*q*E. As a special case, the proposed methods are the ordinary regularization methods when q=1.

Because of (8) and (9), for the *q*-normal linear models, the $L_{q}$-likelihood-based regularization methods are essentially the same as the penalized least square (2). In other words, we implicitly use the $L_{q}$-likelihood-based regularization methods when we apply the ordinary LASSO, SCAD, and MCP to data with heavy-tailed errors.

## 4. Numerical Experiments

In this section, we describe the results of our numerical experiments and compare the proposed methods. Here, we focus on model selection and generalization.

Our methods do not require additional implementations because the LASSO, SCAD, and MCP are already implemented in software packages. In the experiments, we use the ncvreg package of the software R.

### 4.1. Setting

The procedure for the experiments is as follows. We fix the value *q* of the *q*-normal linear model and the $L_{q}$-likelihood, the dimension *d* of the parameter θ, the ratio of nonzero components $r_{\mathrm{nz}}$ of θ, the true value $\theta_{0}$ of the nonzero components of θ, and the sample size *n*. The value of *q* is selected from 1,13/11,3/2,5/3,2,2.01,2.1, and 2.5, where q=13/11 is the *t*-distribution with ν=10 degrees of freedom, q=3/2 is the *t*-distribution with ν=3 degrees of freedom, and q=5/3 is the *t*-distribution with ν=2 degrees of freedom. The sample size is n=100 or n=1000. The true parameter consists of $d\times r_{\mathrm{nz}}$$\theta_{0}$s and $d\times (1-r_{\mathrm{nz}})$ zeros. All cases are illustrated in Table 1.

For each of m=1000 trials, we create the design matrix *X* using the rnorm() function in R. The response y is generated as *q*-normal random variables using the qGaussian package. For the estimation, we apply the ncvreg() function to (y,X) with the default options; for example, the values of the tuning parameters are a=3.7 and γ=3.

To select one model and one estimate from a sequence of parameter estimates generated by a method, we use the AIC and BIC:

(10) $$\begin{array}{c} \mathrm{AIC}=-2\log p\left(y|\hat{\theta }\right)+2d^{\prime }, \end{array}$$

(11) $$\begin{array}{c} \mathrm{BIC}=-2\log p\left(y|\hat{\theta }\right)+d^{\prime }\log n, \end{array}$$

where $d^{\prime }$ is the dimension of parameters of the model under consideration. Moreover, we use other criteria based on the $L_{q}$-likelihood:

(12) $$\begin{array}{c} L_{q}\text{-AIC}=-2L_{q}p\left(y|\hat{\theta }\right)+2d^{\prime }, \end{array}$$

(13) $$\begin{array}{c} L_{q}\text{-BIC}=-2L_{q}p\left(y|\hat{\theta }\right)+d^{\prime }\log n. \end{array}$$

For a sequence $\left({\hat{\theta }}_{\left(k\right)}\right)$ made by each of the methods, let $I_{\left(k\right)}=\left\{i|\;{\hat{\theta }}_{\left(k\right)}^{i}\neq 0\right\}$ and ${\hat{\theta }}_{\mathrm{MLE}}^{\left(k\right)}$ the MLE of the model $M_{\left(k\right)}=\left\{p\left(\cdot |\theta \right)|\;\theta^{j}=0\;\;\left(j\notin I_{\left(k\right)}\right)\right\}$. We call (10) with $\hat{\theta }={\hat{\theta }}_{\mathrm{MLE}}^{\left(k\right)}$ AIC1, and (10) with $\hat{\theta }={\hat{\theta }}_{\left(k\right)}$ AIC2. Similarly, (11) with $\hat{\theta }={\hat{\theta }}_{\mathrm{MLE}}^{\left(k\right)}$ is BIC1, and (11) with $\hat{\theta }={\hat{\theta }}_{\left(k\right)}$ is BIC2. The $L_{q}$-AIC and $L_{q}$-BIC are referred to in the same manner; for example, (12) with $\hat{\theta }={\hat{\theta }}_{\mathrm{MLE}}^{\left(k\right)}$ is $L_{q}$-AIC1. Note that AIC1, BIC1, $L_{q}$-AIC1, and $L_{q}$-BIC1 are available only when the MLE exists; AIC2, BIC2, $L_{q}$-AIC2, and $L_{q}$-BIC2 are always applicable. Finally, we used cross-validation (CV) in addition to these information criteria.

### 4.2. Result

The results are presented in Figure 1, Figure 2, Figure 3, Figure 4, Figure 5, Figure 6, Figure 7, Figure 8, Figure 9, Figure 10, Figure 11, Figure 12, Figure 13, Figure 14, Figure 15, Figure 16, Figure 17, Figure 18, Figure 19, Figure 20, Figure 21 and Figure 22, which report the best result for each method based on the various information criteria. We present the tables of the results of the numerical experiments in the Supplementary Material. In the figures, white bars represent LASSO, gray bars represent SCAD, and black bars represent MCP.

The model selection results are reported in Figure 1, Figure 2, Figure 3, Figure 4, Figure 5, Figure 6, Figure 7, Figure 8, Figure 9, Figure 10, Figure 11, Figure 12, Figure 13 and Figure 14. The vertical axis indicates the number of trials (among m=1000 trials) where a method selects the true model. Here, a larger value is better. The horizontal axis shows the value of $\theta_{0}$.

**Figure 15:**
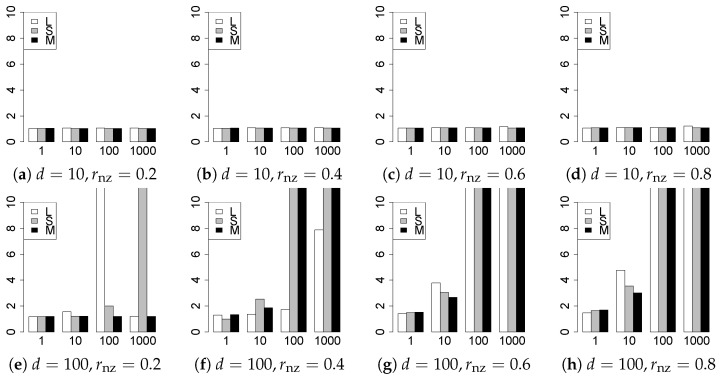
Generalization error for q=1,n=100.

**Figure 16:**
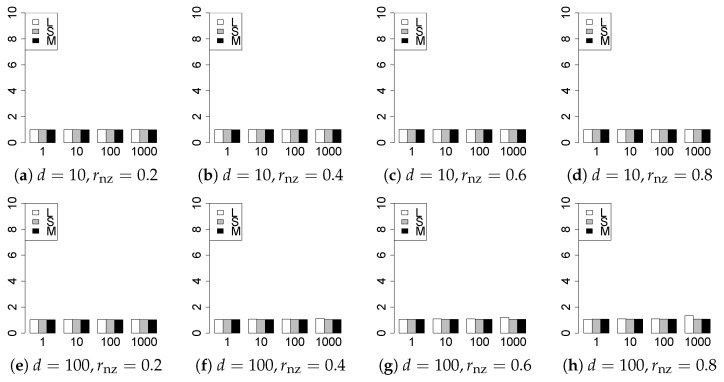
Generalization error for q=1,n=1000.

**Figure 17:**
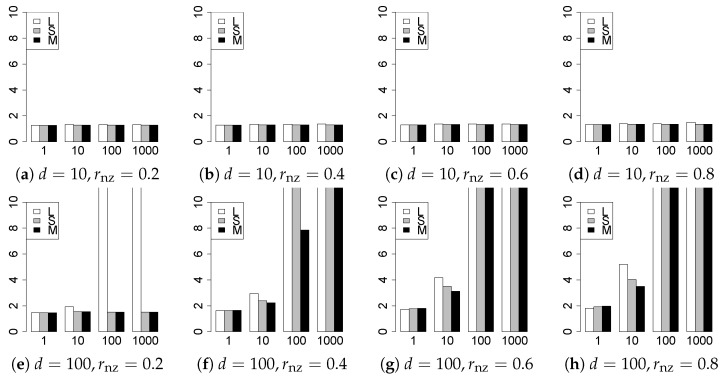
Generalization error for q=13/11,n=100.

**Figure 18:**
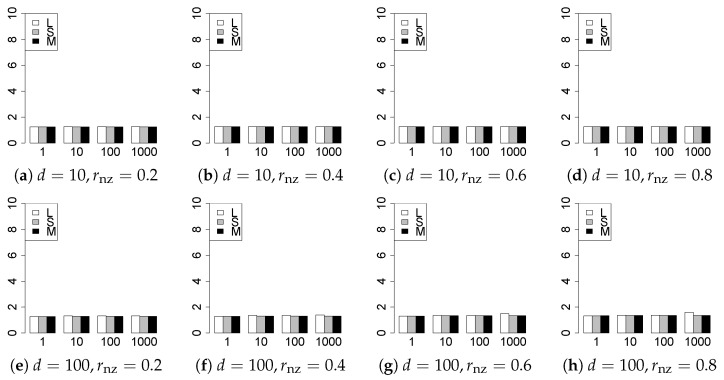
Generalization error for q=13/11,n=1000.

**Figure 19:**
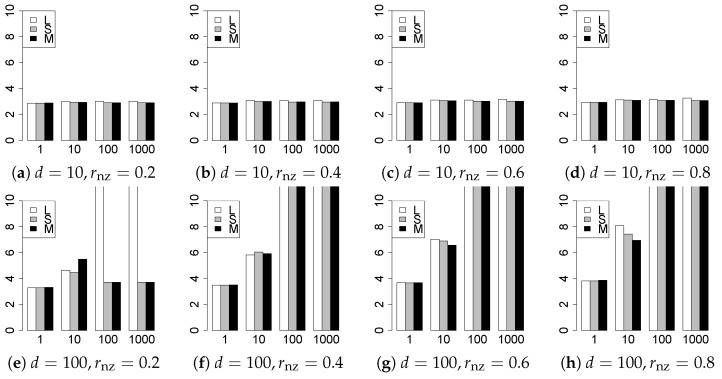
Generalization error for q=3/2,n=100.

**Figure 20:**
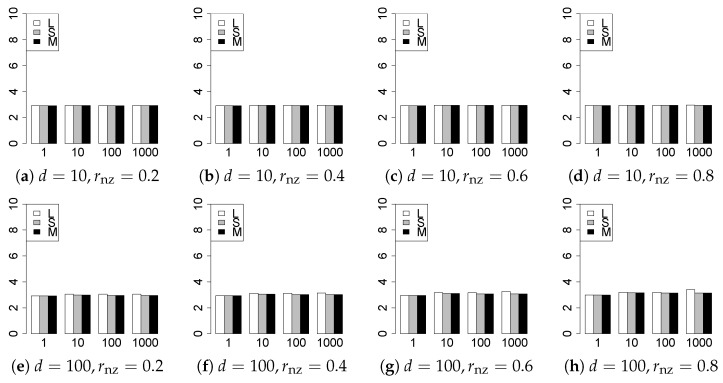
Generalization error for q=3/2,n=1000.

**Figure 21:**
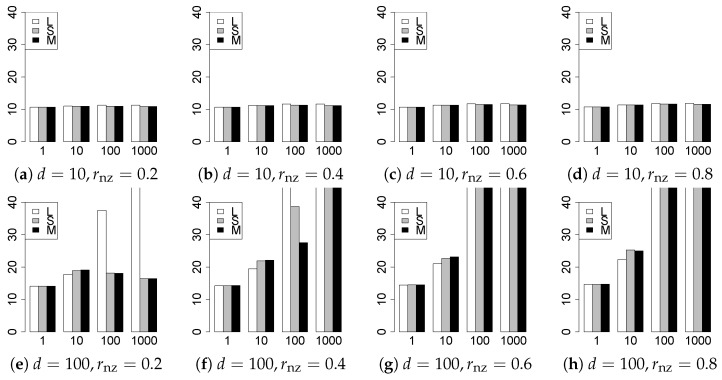
Generalization error for q=5/3,n=100.

**Figure 22:**
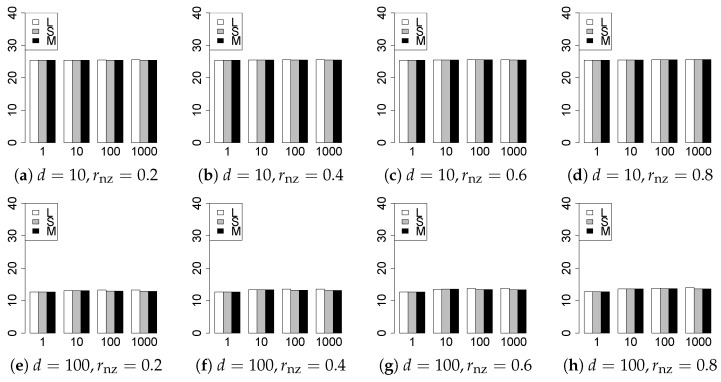
Generalization error for q=5/3,n=1000.

The generalization results are reported in Figure 15, Figure 16, Figure 17, Figure 18, Figure 19, Figure 20, Figure 21 and Figure 22. To evaluate the generalization error of the proposed methods, we newly make m=1000 independent copies $\left\{\left(y_{1}^{\prime },X_{1}^{\prime }\right),\cdots ,\left(y_{m}^{\prime },X_{m}^{\prime }\right)\right\}$ in each trial. We computed the difference between $\left(y_{1}^{\prime },\cdots ,y_{m}^{\prime }\right)$ and the *m* predictions using each of the methods. The vertical axis indicates the average prediction error over *m* trials. In this case, a smaller value is better. The scaling of Figure 21 and Figure 22 (q=5/3) is different from that of the other figures.

Our first concern is whether the proposed methods work well. The results for q=1 can be regarded as a reference for the other values of *q*. The figures show that the proposed methods work well in both model selection and generalization, especially for q<2. The methods also perform well in terms of model selection for q=2,2.01, and 2.1. However, they perform poorly for q=2.5 in terms of model selection and for q≥2 in terms of generalization. As anticipated, a large *q* makes the problem difficult.

Second, we evaluate the performance of the proposed methods, finding that the MCP performs best in most cases. In a few cases, the MCP performed similarly to or slightly worse than the other methods. For model selection, the cases with q=1,n=1000 and large $\theta_{0}$ are exceptions. Furthermore, the LASSO performed worse than the SCAD and MCP.

Third, we consider the effect of $r_{\mathrm{nz}}$, $\theta_{0}$, *d*, and *n*, in addition to *q*. The cases with large $r_{\mathrm{nz}}$ and/or small $\theta_{0}$ are difficult. Moreover, a large *d* makes the problems difficult. However, if we have a small *q* (1≤q<2), large $\theta_{0}$ ($\theta_{0}=10^{2},10^{3}$) and small $r_{\mathrm{nz}}$, the problems with large *d* can be easier than those with small *d*. Furthermore, a small *n* makes the problems difficult in a similar manner to a large *d*. These observations imply that, for 1≤q<2, small-sample problems can be easier than large-sample problems if $r_{\mathrm{nz}}$ is small and $\theta_{0}$ is large.

Fourth, the choice of information criterion changes the methods’ performance. In terms of model selection, BIC2 was mostly the best for many values of *q*. For 3/2≤q≤2.1, BIC1 was a little better than BIC2 if BIC1 was available. For q=1 and 13/11, BIC2 was better than BIC1. AIC1 and AIC2 were as good as BICs for 2≤q≤2.1. Moreover, the $L_{q}$-BIC1 and -BIC2 were best only for q=3/2, when BIC1 and BIC2 performed just as well. Overall, the $L_{q}$-information criteria performed poorly.

Furthermore, in terms of generalization, BIC2 was mostly the best. AIC2 was as good as BIC2, whereas AIC2 was sometimes a little worse than BIC2. The information criteria using the $L_{q}$-likelihood were poor for q=13/11. For q=1,3/2, and 5/3, the $L_{q}$-information criteria worked as well as the ordinary criteria and CV, except for some cases. The performance of CV was mostly good, but was occasionally very poor.

In summary, using an appropriate criterion, the proposed methods perform well for linear models with slightly heavy-tailed errors (1≤q<2). Moreover, the proposed methods work in terms of model selection, even if the error is heavy-tailed (2≤q<2.5). Overall, we recommend using the MCP and BIC2.

## 5. Conclusions

We proposed regularization methods for *q*-normal linear models based on the $L_{q}$-likelihood. The proposed methods coincide with the ordinary regularization methods. Our methods perform well for slightly heavy-tailed errors (1≤q<2) in terms of model selection and generalization. Moreover, they work well in terms of model selection for heavy-tailed errors (2≤q<2.5). A theoretical analysis of the proposed methods is left to future work.

## Figures and Tables

**Figure 1:**
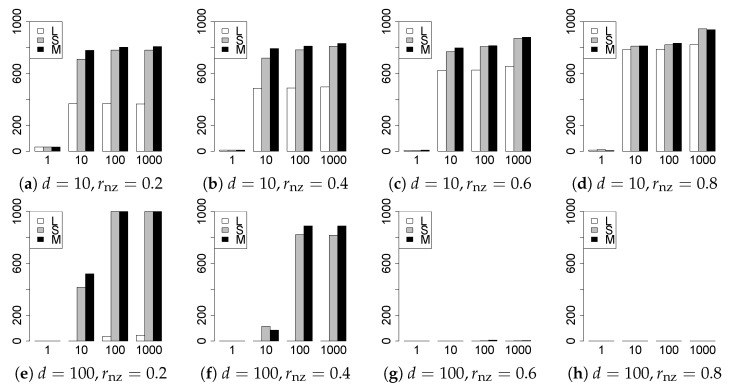
Model selection for q=1,n=100.

**Figure 2:**
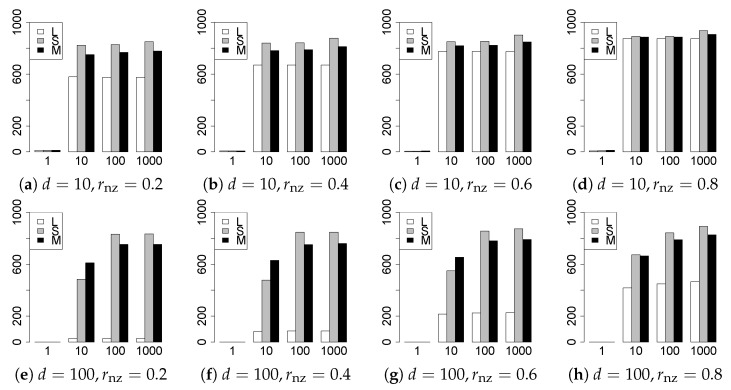
Model selection for q=1,n=1000.

**Figure 3:**
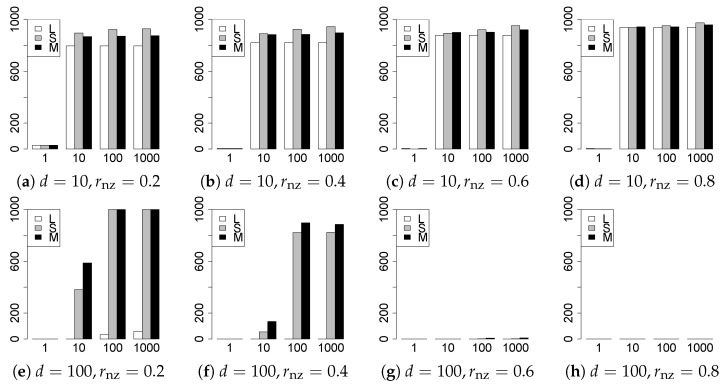
Model selection for q=13/11,n=100.

**Figure 4:**
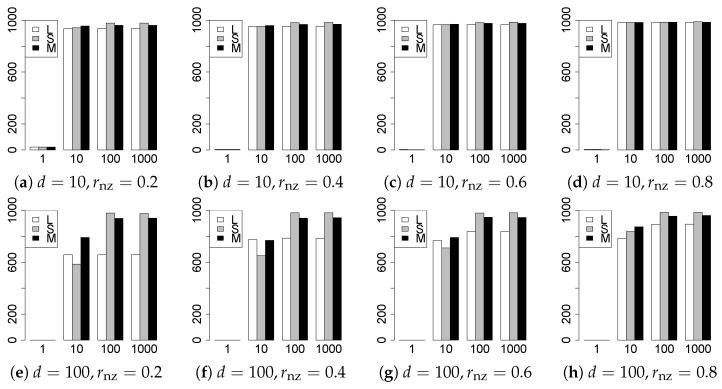
Model selection for q=13/11,n=1000.

**Figure 5:**
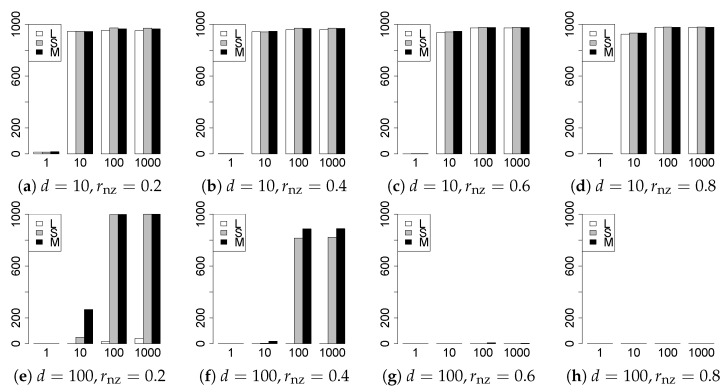
Model selection for q=3/2,n=100.

**Figure 6:**
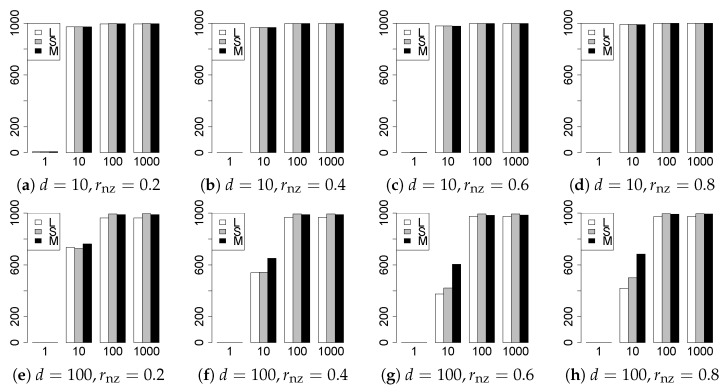
Model selection for q=3/2,n=1000.

**Figure 7:**
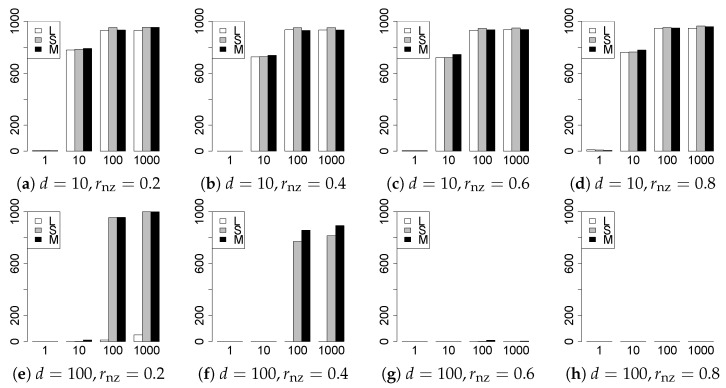
Model selection for q=5/3,n=100.

**Figure 8:**
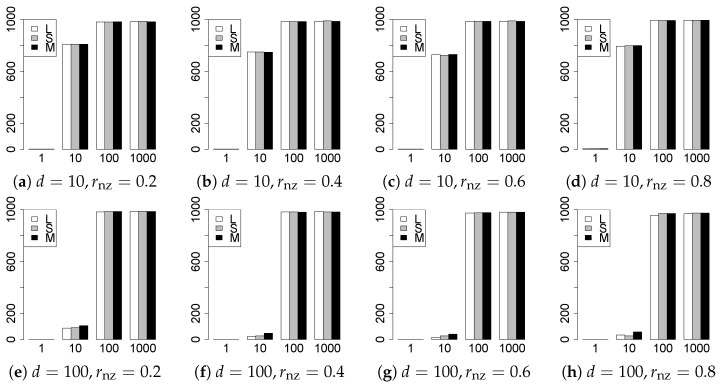
Model selection for q=5/3,n=1000.

**Figure 9:**
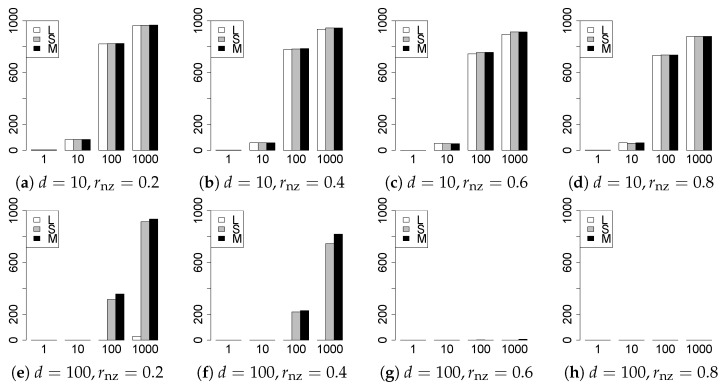
Model selection for q=2,n=100.

**Figure 10:**
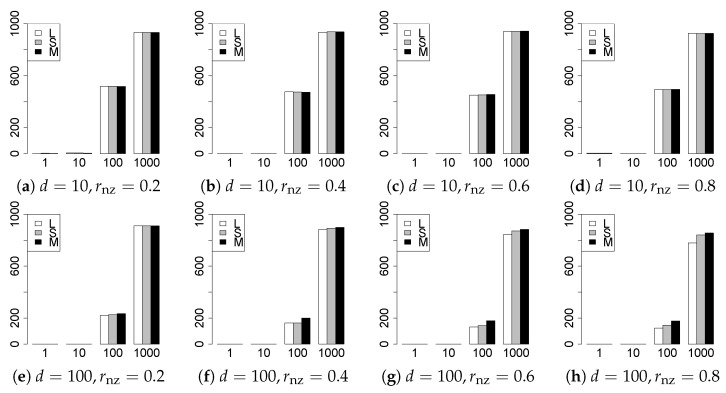
Model selection for q=2,n=1000.

**Figure 11:**
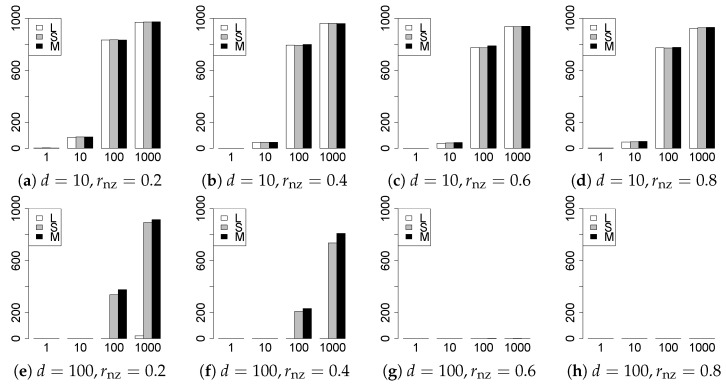
Model selection for q=2.01,n=100.

**Figure 12:**
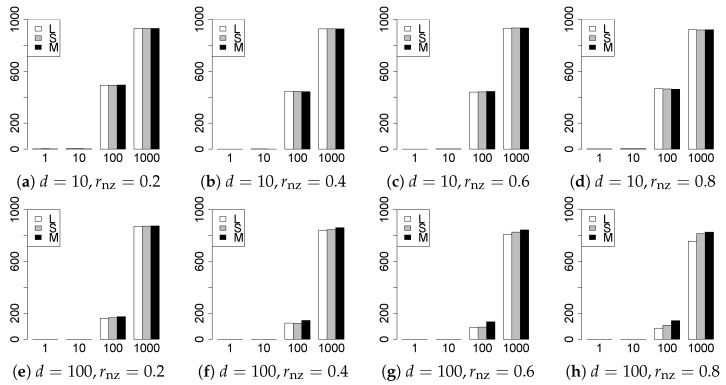
Model selection for q=2.01,n=1000.

**Figure 13:**
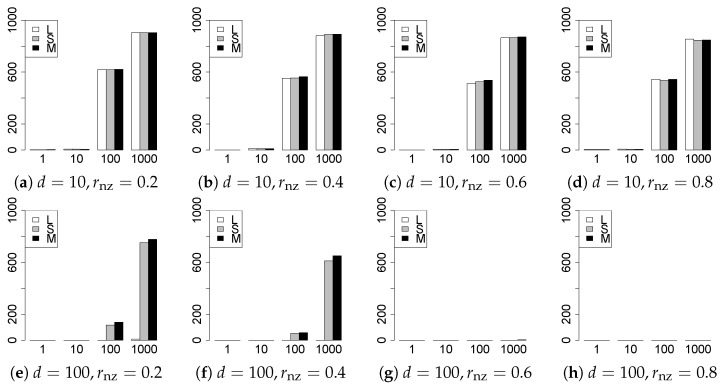
Model selection for q=2.1,n=100.

**Figure 14:**
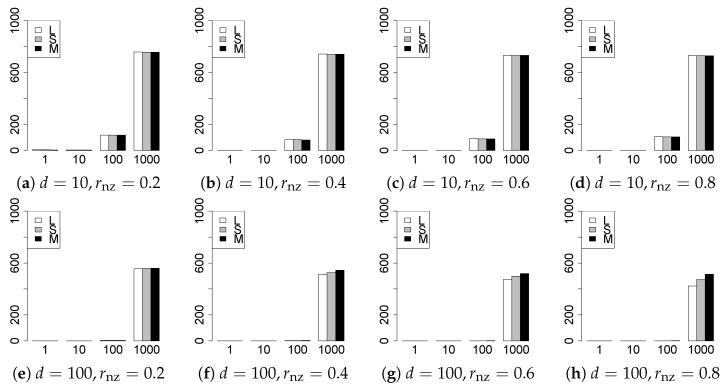
Model selection for q=2.1,n=1000.

**Table 1:** All cases in the experiments. Each case is studied for the values of *q* and *n*.

$\theta_{0}$	$r_{\mathrm{nz}}=0.2$		$r_{\mathrm{nz}}=0.4$		$r_{\mathrm{nz}}=0.6$		$r_{\mathrm{nz}}=0.8$	
	d=10	d=100	d=10	d=100	d=10	d=100	d=10	d=100
$10^{0}$	1	5	9	13	17	21	25	29
$10^{1}$	2	6	10	14	18	22	26	30
$10^{2}$	3	7	11	15	19	23	27	31
$10^{3}$	4	8	12	16	20	24	28	32

## Supplementary Materials

The following are available online at https://www.mdpi.com/1099-4300/22/9/1036/s1, Tables S1–S34: The results of the numerical experiments.
