# Supplementary material for: Regularization Methods Based on the Lq-Likelihood for Linear Models with Heavy-Tailed Errors
Source: Entropy (Basel). 2020 Sep 16;22(9):1036. doi: 10.3390/e22091036 (PMC7597096; doi:10.3390/e22091036)
Supplement: Supplementary file 1 [file entropy-22-01036-s001.zip › entropy-927958-supplementary.pdf]

Supplementary Materials for "Regularization Methods Based on the L_q-Likelihood for Linear Models With Heavy-Tailed Errors"

Yoshihiro Hirose*
Hokkaido University, Japan

A Tables

The results of the numerical experiments are presented as tables.

A.1 Best Performance

The partial results are presented in Tables S1–S22, which report the best result for each method based on the various information criteria. The figures in the manuscript are based on these tables.

The model selection results are reported in Tables S1–S14. Each number shows the number of trials (among $m = 1{,}000$ trials) where a method selects the true model. Here, a larger value is better.

The generalization results are reported in Tables S15–S22. To evaluate the generalization error of the proposed methods, we newly make $m = 1{,}000$ independent copies $\{(\boldsymbol{y}'_1, X'_1), \ldots, (\boldsymbol{y}'_m, X'_m)\}$ in each trial. We computed the difference between $(\boldsymbol{y}'_1, \ldots, \boldsymbol{y}'_m)$ and the m predictions using each of the methods. Each value shows the average prediction error over m trials. In this case, a smaller value is better.

A.2 Complete Results

The complete results of the experiments are presented as tables (Tables S23–S34). The case numbers in the tables are shown in Table 1 of the manuscript. In the tables, "a" after the case number indicates the sample size $n = 100$, and "b" means $n = 1{,}000$

The results on model selection are reported in Tables S23–S30. The "Seq" column indicates the number of trials (among $m = 1{,}000$ trials) when each sequence of estimates includes the true model. The other columns show the

*Faculty of Information Science and Technology, Hokkaido University. Kita 14, Nishi 9, Kita-ku, Sapporo, Hokkaido, 060-0814 Japan. hirose@ist.hokudai.ac.jp

Table S1: The result on model selection for $q = 1$ and $n = 100$. Each number indicates how many trials a method correctly selects the true model using an appropriate information criterion.

θ_0	Method	$r_{\mathrm{nz}} = 0.2$		$r_{\mathrm{nz}} = 0.4$		$r_{\mathrm{nz}} = 0.6$		$r_{\mathrm{nz}} = 0.8$	
		$d = 10$	$d = 100$	$d = 10$	$d = 100$	$d = 10$	$d = 100$	$d = 10$	$d = 100$
10^0	LASSO	32	0	9	0	5	0	8	0
	SCAD	32	0	9	0	5	0	12	0
	MCP	32	0	8	0	8	0	5	0
10^1	LASSO	366	0	486	0	625	0	784	0
	SCAD	710	415	719	114	768	0	812	0
	MCP	778	519	793	84	797	0	813	0
10^2	LASSO	366	35	487	0	627	0	788	0
	SCAD	781	1,000	782	822	808	2	821	0
	MCP	802	1,000	812	890	814	8	833	0
10^3	LASSO	365	46	496	0	655	0	823	0
	SCAD	781	1,000	809	817	871	2	945	0
	MCP	808	1,000	832	889	880	4	938	0

number of trials when a method selects the true model using each information criterion. A larger value is better.

The results on generalization are reported in Tables S31–S34. The "generalization" columns show the average prediction errors over $m = 1{,}000$ trials. Here, a smaller value is better.

The symbol "-" indicates that the information criterion is not available. However, in Tables S23 and S31, "-" in a column of a L_q-criterion means the value is the same as that of the corresponding non-L_q-criterion.

Table S2: The result on model selection for $q = 1$ and $n = 1{,}000$.

θ_0	Method	$r_{\mathrm{nz}} = 0.2$		$r_{\mathrm{nz}} = 0.4$		$r_{\mathrm{nz}} = 0.6$		$r_{\mathrm{nz}} = 0.8$	
		$d = 10$	$d = 100$	$d = 10$	$d = 100$	$d = 10$	$d = 100$	$d = 10$	$d = 100$
10^0	LASSO	13	0	7	0	5	0	8	0
	SCAD	13	0	7	0	5	0	10	0
	MCP	13	0	8	0	7	0	12	0
10^1	LASSO	579	28	670	81	774	216	875	417
	SCAD	823	485	840	478	851	549	892	673
	MCP	750	611	781	630	820	655	886	665
10^2	LASSO	577	28	670	85	774	224	875	449
	SCAD	828	833	842	847	853	856	891	842
	MCP	768	753	788	751	823	781	886	790
10^3	LASSO	577	28	670	85	774	226	875	466
	SCAD	851	834	878	848	902	873	935	892
	MCP	778	754	812	760	848	791	906	827

Table S3: The result on model selection for $q = 13/11$ and $n = 100$.

θ_0	Method	$r_{\mathrm{nz}} = 0.2$		$r_{\mathrm{nz}} = 0.4$		$r_{\mathrm{nz}} = 0.6$		$r_{\mathrm{nz}} = 0.8$	
		$d = 10$	$d = 100$	$d = 10$	$d = 100$	$d = 10$	$d = 100$	$d = 10$	$d = 100$
10^0	LASSO	29	0	4	0	3	0	3	0
	SCAD	29	0	4	0	1	0	2	0
	MCP	29	0	4	0	3	0	2	0
10^1	LASSO	796	0	821	0	877	0	939	0
	SCAD	896	382	890	55	895	0	939	0
	MCP	869	588	884	135	901	0	944	0
10^2	LASSO	797	32	821	0	878	0	939	0
	SCAD	924	1,000	925	822	921	1	953	0
	MCP	872	1,000	885	897	902	7	944	0
10^3	LASSO	797	59	821	0	879	0	940	0
	SCAD	929	1,000	946	823	953	1	975	0
	MCP	875	1,000	897	886	921	8	959	0

Table S4: The result on model selection for $q = 13/11$ and $n = 1{,}000$.

θ_0	Method	$r_{\mathrm{nz}} = 0.2$		$r_{\mathrm{nz}} = 0.4$		$r_{\mathrm{nz}} = 0.6$		$r_{\mathrm{nz}} = 0.8$	
		$d = 10$	$d = 100$	$d = 10$	$d = 100$	$d = 10$	$d = 100$	$d = 10$	$d = 100$
10^0	LASSO	21	0	3	0	2	0	3	0
	SCAD	21	0	2	0	1	0	4	0
	MCP	22	0	2	0	1	0	1	0
10^1	LASSO	936	658	951	774	965	768	982	785
	SCAD	941	585	951	651	965	711	982	838
	MCP	955	792	958	769	969	792	982	873
10^2	LASSO	936	657	952	786	965	837	982	893
	SCAD	978	980	981	981	981	980	985	984
	MCP	961	939	967	940	975	947	984	956
10^3	LASSO	936	660	952	784	965	838	982	894
	SCAD	977	977	983	981	984	982	988	984
	MCP	961	940	968	944	975	945	984	961

Table S5: The result on model selection for $q = 3/2$ and $n = 100$.

θ_0	Method	$r_{\mathrm{nz}} = 0.2$		$r_{\mathrm{nz}} = 0.4$		$r_{\mathrm{nz}} = 0.6$		$r_{\mathrm{nz}} = 0.8$	
		$d = 10$	$d = 100$	$d = 10$	$d = 100$	$d = 10$	$d = 100$	$d = 10$	$d = 100$
10^0	LASSO	13	0	1	0	0	0	2	0
	SCAD	13	0	1	0	2	0	1	0
	MCP	16	0	1	0	1	0	1	0
10^1	LASSO	946	0	943	0	936	0	924	0
	SCAD	947	51	941	4	941	0	933	0
	MCP	945	264	947	19	947	0	933	0
10^2	LASSO	952	16	958	0	973	0	977	0
	SCAD	972	997	971	816	977	0	979	0
	MCP	965	999	969	887	976	7	978	0
10^3	LASSO	951	41	958	0	973	0	978	0
	SCAD	971	1,000	971	822	977	1	979	0
	MCP	965	1,000	969	889	976	4	978	0

Table S6: The result on model selection for $q = 3/2$ and $n = 1{,}000$.

θ_0	Method	$r_{\mathrm{nz}} = 0.2$		$r_{\mathrm{nz}} = 0.4$		$r_{\mathrm{nz}} = 0.6$		$r_{\mathrm{nz}} = 0.8$	
		$d = 10$	$d = 100$	$d = 10$	$d = 100$	$d = 10$	$d = 100$	$d = 10$	$d = 100$
10^0	LASSO	7	0	1	0	1	0	1	0
	SCAD	8	0	1	0	2	0	0	0
	MCP	8	0	1	0	2	0	0	0
10^1	LASSO	971	735	967	539	978	376	990	419
	SCAD	971	726	966	543	978	421	990	501
	MCP	971	762	966	651	977	605	989	683
10^2	LASSO	994	963	996	968	996	975	999	973
	SCAD	997	994	998	993	998	994	999	996
	MCP	996	989	997	989	997	984	999	992
10^3	LASSO	994	963	996	967	996	975	999	974
	SCAD	998	995	998	993	998	994	999	996
	MCP	996	989	997	988	997	985	999	993

Table S7: The result on model selection for $q = 5/3$ and $n = 100$.

θ_0	Method	$r_{\mathrm{nz}} = 0.2$		$r_{\mathrm{nz}} = 0.4$		$r_{\mathrm{nz}} = 0.6$		$r_{\mathrm{nz}} = 0.8$	
		$d = 10$	$d = 100$	$d = 10$	$d = 100$	$d = 10$	$d = 100$	$d = 10$	$d = 100$
10^0	LASSO	4	0	1	0	3	0	11	0
	SCAD	5	0	1	0	3	0	9	0
	MCP	4	0	1	0	3	0	5	0
10^1	LASSO	779	0	727	0	722	0	762	0
	SCAD	785	1	728	0	722	0	764	0
	MCP	791	12	739	0	745	0	779	0
10^2	LASSO	929	11	935	0	932	0	947	0
	SCAD	953	955	950	770	945	1	952	0
	MCP	933	956	931	857	935	9	949	0
10^3	LASSO	930	50	933	0	935	0	948	0
	SCAD	954	1,000	951	815	949	0	964	0
	MCP	954	1,000	934	893	937	3	959	0

Table S8: The result on model selection for $q = 5/3$ and $n = 1{,}000$.

θ_0	Method	$r_{\text{nz}} = 0.2$		$r_{\text{nz}} = 0.4$		$r_{\text{nz}} = 0.6$		$r_{\text{nz}} = 0.8$	
		$d = 10$	$d = 100$	$d = 10$	$d = 100$	$d = 10$	$d = 100$	$d = 10$	$d = 100$
10^0	LASSO	2	0	2	0	2	0	4	0
	SCAD	2	0	2	0	2	0	5	0
	MCP	2	0	1	0	2	0	4	0
10^1	LASSO	810	87	750	22	729	18	794	35
	SCAD	810	90	750	27	725	27	798	28
	MCP	810	106	749	48	731	41	799	58
10^2	LASSO	981	984	984	984	986	975	994	956
	SCAD	981	987	984	983	986	977	994	971
	MCP	983	987	984	982	986	979	994	972
10^3	LASSO	983	988	986	987	986	981	995	969
	SCAD	986	989	989	983	990	981	995	974
	MCP	983	987	986	983	987	981	995	974

Table S9: The result on model selection for $q = 2$ and $n = 100$.

θ_0	Method	$r_{\text{nz}} = 0.2$		$r_{\text{nz}} = 0.4$		$r_{\text{nz}} = 0.6$		$r_{\text{nz}} = 0.8$	
		$d = 10$	$d = 100$	$d = 10$	$d = 100$	$d = 10$	$d = 100$	$d = 10$	$d = 100$
10^0	LASSO	3	0	2	0	0	0	2	0
	SCAD	3	0	2	0	0	0	2	0
	MCP	3	0	1	0	0	0	2	0
10^1	LASSO	81	0	57	0	53	0	58	0
	SCAD	83	0	58	0	50	0	54	0
	MCP	83	0	57	0	50	0	57	0
10^2	LASSO	821	0	777	0	745	0	732	0
	SCAD	823	314	782	219	755	2	735	0
	MCP	824	357	786	229	756	0	736	0
10^3	LASSO	961	29	935	0	895	0	877	0
	SCAD	966	915	944	746	913	1	879	0
	MCP	967	935	945	820	913	7	879	0

Table S10: The result on model selection for $q = 2$ and $n = 1{,}000$.

θ_0	Method	$r_{\text{nz}} = 0.2$		$r_{\text{nz}} = 0.4$		$r_{\text{nz}} = 0.6$		$r_{\text{nz}} = 0.8$	
		$d = 10$	$d = 100$	$d = 10$	$d = 100$	$d = 10$	$d = 100$	$d = 10$	$d = 100$
10^0	LASSO	1	0	1	0	0	0	3	0
	SCAD	3	0	1	0	0	0	3	0
	MCP	2	0	0	0	0	0	3	0
10^1	LASSO	5	0	1	0	1	0	1	0
	SCAD	5	0	1	0	1	0	1	0
	MCP	4	0	0	0	0	0	1	0
10^2	LASSO	518	221	476	162	451	132	494	123
	SCAD	519	226	475	162	453	142	494	145
	MCP	518	235	473	200	455	180	495	178
10^3	LASSO	934	910	936	883	941	843	926	779
	SCAD	933	910	938	892	942	871	925	840
	MCP	933	911	938	899	943	884	926	855

Table S11: The result on model selection for $q = 2.01$ and $n = 100$.

θ_0	Method	$r_{\text{nz}} = 0.2$		$r_{\text{nz}} = 0.4$		$r_{\text{nz}} = 0.6$		$r_{\text{nz}} = 0.8$	
		$d = 10$	$d = 100$	$d = 10$	$d = 100$	$d = 10$	$d = 100$	$d = 10$	$d = 100$
10^0	LASSO	1	0	0	0	0	0	1	0
	SCAD	2	0	0	0	0	0	1	0
	MCP	1	0	0	0	0	0	1	0
10^1	LASSO	84	0	44	0	37	0	48	0
	SCAD	86	0	45	0	40	0	49	0
	MCP	87	0	46	0	43	0	52	0
10^2	LASSO	834	1	793	0	775	0	773	0
	SCAD	835	338	792	210	774	0	772	0
	MCP	834	377	799	232	788	3	776	0
10^3	LASSO	971	22	961	0	937	0	921	0
	SCAD	972	894	961	737	936	1	927	0
	MCP	973	916	960	810	939	3	930	0

Table S12: The result on model selection for $q = 2.01$ and $n = 1{,}000$.

θ_0	Method	$r_{\text{nz}} = 0.2$		$r_{\text{nz}} = 0.4$		$r_{\text{nz}} = 0.6$		$r_{\text{nz}} = 0.8$	
		$d = 10$	$d = 100$	$d = 10$	$d = 100$	$d = 10$	$d = 100$	$d = 10$	$d = 100$
10^0	LASSO	2	0	0	0	0	0	1	0
	SCAD	3	0	0	0	0	0	1	0
	MCP	1	0	0	0	0	0	1	0
10^1	LASSO	6	0	1	0	1	0	3	0
	SCAD	6	0	1	0	1	0	3	0
	MCP	2	0	0	0	1	0	3	0
10^2	LASSO	493	161	447	124	441	92	466	85
	SCAD	493	165	445	123	442	94	464	107
	MCP	495	174	443	144	445	134	463	143
10^3	LASSO	931	871	928	839	933	808	922	757
	SCAD	931	872	929	846	935	826	920	815
	MCP	931	875	929	861	935	844	921	827

Table S13: The result on model selection for $q = 2.1$ and $n = 100$.

θ_0	Method	$r_{\text{nz}} = 0.2$		$r_{\text{nz}} = 0.4$		$r_{\text{nz}} = 0.6$		$r_{\text{nz}} = 0.8$	
		$d = 10$	$d = 100$	$d = 10$	$d = 100$	$d = 10$	$d = 100$	$d = 10$	$d = 100$
10^0	LASSO	2	0	0	0	0	0	3	0
	SCAD	2	0	0	0	0	0	3	0
	MCP	3	0	0	0	0	0	3	0
10^1	LASSO	6	0	9	0	4	0	6	0
	SCAD	6	0	9	0	4	0	4	0
	MCP	4	0	10	0	5	0	5	0
10^2	LASSO	619	0	553	0	512	0	545	0
	SCAD	618	117	554	53	525	0	535	0
	MCP	621	139	564	58	537	0	545	0
10^3	LASSO	903	9	881	0	864	0	854	0
	SCAD	903	753	890	612	866	1	846	0
	MCP	904	778	892	652	871	3	847	0

Table S14: The result on model selection for $q = 2.1$ and $n = 1{,}000$.

θ_0	Method	$r_{\mathrm{nz}} = 0.2$		$r_{\mathrm{nz}} = 0.4$		$r_{\mathrm{nz}} = 0.6$		$r_{\mathrm{nz}} = 0.8$	
		$d = 10$	$d = 100$	$d = 10$	$d = 100$	$d = 10$	$d = 100$	$d = 10$	$d = 100$
10^0	LASSO	6	0	1	0	0	0	0	0
	SCAD	6	0	1	0	0	0	0	0
	MCP	4	0	1	0	0	0	0	0
10^1	LASSO	4	0	0	0	0	0	0	0
	SCAD	4	0	0	0	0	0	0	0
	MCP	4	0	0	0	0	0	0	0
10^2	LASSO	118	3	82	1	92	0	106	0
	SCAD	118	3	84	2	89	0	105	0
	MCP	119	4	81	3	90	1	105	1
10^3	LASSO	759	556	744	514	734	474	732	422
	SCAD	758	558	741	526	732	498	730	472
	MCP	758	560	743	544	734	518	731	513

Table S15: The result on generalization for $q = 1$ and $n = 100$. Each value indicates the average generalization error among $m = 1{,}000$ trials.

θ_0	Method	$r_{\mathrm{nz}} = 0.2$		$r_{\mathrm{nz}} = 0.4$		$r_{\mathrm{nz}} = 0.6$		$r_{\mathrm{nz}} = 0.8$	
		$d = 10$	$d = 100$	$d = 10$	$d = 100$	$d = 10$	$d = 100$	$d = 10$	$d = 100$
10^0	LASSO	1.03	1.18	1.05	1.31	1.06	1.40	1.07	1.47
	SCAD	1.04	1.19	1.05	1.34	1.07	1.49	1.08	1.64
	MCP	1.05	1.20	1.06	1.37	1.07	1.53	1.09	1.70
10^1	LASSO	1.07	1.55	1.09	2.53	1.11	3.78	1.11	4.76
	SCAD	1.04	1.22	1.06	1.86	1.08	3.05	1.10	3.54
	MCP	1.04	1.22	1.06	1.73	1.08	2.68	1.10	3.02
10^2	LASSO	1.07	21.02	1.09	99.2	1.10	225.5	1.11	321.6
	SCAD	1.04	1.20	1.06	16.5	1.08	127.7	1.10	174.2
	MCP	1.04	1.20	1.06	7.88	1.08	85.2	1.10	119.1
10^3	LASSO	1.07	1984	1.11	9762	1.17	22405	1.23	32013
	SCAD	1.04	1.20	1.06	1557	1.07	12795	1.09	17287
	MCP	1.04	1.20	1.06	636	1.08	8537	1.09	11803

Table S16: The result on generalization for $q = 1$ and $n = 1{,}000$.

θ_0	Method	$r_{\mathrm{nz}} = 0.2$		$r_{\mathrm{nz}} = 0.4$		$r_{\mathrm{nz}} = 0.6$		$r_{\mathrm{nz}} = 0.8$	
		$d = 10$	$d = 100$	$d = 10$	$d = 100$	$d = 10$	$d = 100$	$d = 10$	$d = 100$
10^0	LASSO	1.00	1.02	1.00	1.04	1.01	1.05	1.01	1.06
	SCAD	1.00	1.02	1.00	1.04	1.01	1.05	1.01	1.07
	MCP	1.00	1.02	1.00	1.04	1.01	1.06	1.01	1.07
10^1	LASSO	1.01	1.05	1.01	1.08	1.01	1.09	1.01	1.10
	SCAD	1.00	1.02	1.00	1.04	1.01	1.06	1.01	1.08
	MCP	1.00	1.02	1.01	1.04	1.01	1.06	1.01	1.08
10^2	LASSO	1.01	1.05	1.01	1.08	1.01	1.09	1.01	1.10
	SCAD	1.00	1.02	1.00	1.04	1.01	1.06	1.01	1.08
	MCP	1.00	1.02	1.01	1.04	1.01	1.06	1.01	1.08
10^3	LASSO	1.01	1.06	1.01	1.13	1.01	1.22	1.02	1.33
	SCAD	1.00	1.02	1.00	1.04	1.01	1.06	1.01	1.08
	MCP	1.00	1.02	1.01	1.04	1.01	1.06	1.01	1.08

Table S17: The result on generalization for $q = 13/11$ and $n = 100$.

θ_0	Method	$r_{nz} = 0.2$		$r_{nz} = 0.4$		$r_{nz} = 0.6$		$r_{nz} = 0.8$	
		$d = 10$	$d = 100$	$d = 10$	$d = 100$	$d = 10$	$d = 100$	$d = 10$	$d = 100$
10^0	LASSO	1.27	1.46	1.29	1.63	1.31	1.73	1.32	1.81
	SCAD	1.27	1.47	1.29	1.63	1.31	1.78	1.33	1.92
	MCP	1.27	1.47	1.29	1.65	1.31	1.81	1.33	1.97
10^1	LASSO	1.33	1.93	1.36	2.95	1.37	4.17	1.38	5.19
	SCAD	1.29	1.55	1.31	2.39	1.34	3.51	1.36	4.03
	MCP	1.29	1.55	1.31	2.24	1.34	3.12	1.36	3.51
10^2	LASSO	1.33	21.20	1.36	100.23	1.37	224.32	1.38	325.87
	SCAD	1.28	1.52	1.31	17.21	1.33	127.43	1.36	177.62
	MCP	1.29	1.52	1.31	7.86	1.34	85.82	1.36	122.05
10^3	LASSO	1.32	1965	1.37	9830	1.43	22282	1.51	32492
	SCAD	1.28	1.52	1.31	1540	1.33	12921	1.36	18310
	MCP	1.29	1.52	1.31	728	1.33	8716	1.36	12433

Table S18: The result on generalization for $q = 13/11$ and $n = 1{,}000$.

θ_0	Method	$r_{nz} = 0.2$		$r_{nz} = 0.4$		$r_{nz} = 0.6$		$r_{nz} = 0.8$	
		$d = 10$	$d = 100$	$d = 10$	$d = 100$	$d = 10$	$d = 100$	$d = 10$	$d = 100$
10^0	LASSO	1.25	1.27	1.26	1.29	1.26	1.30	1.26	1.32
	SCAD	1.25	1.27	1.26	1.29	1.26	1.30	1.26	1.32
	MCP	1.25	1.27	1.26	1.29	1.26	1.31	1.26	1.33
10^1	LASSO	1.26	1.32	1.26	1.35	1.26	1.36	1.26	1.37
	SCAD	1.25	1.28	1.26	1.30	1.26	1.33	1.26	1.35
	MCP	1.25	1.28	1.26	1.30	1.26	1.33	1.26	1.35
10^2	LASSO	1.26	1.32	1.26	1.35	1.26	1.36	1.26	1.37
	SCAD	1.25	1.28	1.26	1.30	1.26	1.33	1.26	1.35
	MCP	1.25	1.28	1.26	1.30	1.26	1.33	1.26	1.35
10^3	LASSO	1.26	1.32	1.26	1.39	1.27	1.49	1.27	1.60
	SCAD	1.25	1.28	1.26	1.30	1.26	1.33	1.26	1.35
	MCP	1.25	1.28	1.26	1.30	1.26	1.33	1.26	1.35

Table S19: The result on generalization for $q = 3/2$ and $n = 100$.

θ_0	Method	$r_{nz} = 0.2$		$r_{nz} = 0.4$		$r_{nz} = 0.6$		$r_{nz} = 0.8$	
		$d = 10$	$d = 100$	$d = 10$	$d = 100$	$d = 10$	$d = 100$	$d = 10$	$d = 100$
10^0	LASSO	2.87	3.29	2.89	3.50	2.91	3.68	2.93	3.83
	SCAD	2.87	3.29	2.89	3.50	2.91	3.67	2.93	3.83
	MCP	2.88	3.32	2.89	3.51	2.91	3.69	2.93	3.87
10^1	LASSO	3.00	4.65	3.07	5.82	3.11	7.05	3.13	8.09
	SCAD	2.94	4.47	3.01	6.04	3.07	6.90	3.11	7.44
	MCP	2.93	5.50	3.01	5.93	3.06	6.58	3.10	6.95
10^2	LASSO	3.01	23.33	3.08	103.52	3.11	229.03	3.14	331.08
	SCAD	2.91	3.72	2.96	46.11	3.02	133.22	3.08	180.93
	MCP	2.91	3.72	2.97	11.23	3.02	91.05	3.09	125.76
10^3	LASSO	3.00	1973	3.08	9900	3.16	22441	3.26	32653
	SCAD	2.91	3.72	2.96	1523	3.02	12689	3.08	18575
	MCP	2.91	3.72	2.97	677	3.02	8584	3.08	12104

Table S20: The result on generalization for $q = 3/2$ and $n = 1{,}000$.

θ_0	Method	$r_{\mathrm{nz}} = 0.2$		$r_{\mathrm{nz}} = 0.4$		$r_{\mathrm{nz}} = 0.6$		$r_{\mathrm{nz}} = 0.8$	
		$d = 10$	$d = 100$	$d = 10$	$d = 100$	$d = 10$	$d = 100$	$d = 10$	$d = 100$
10^0	LASSO	2.92	2.92	2.92	2.94	2.92	2.96	2.93	2.98
	SCAD	2.92	2.92	2.92	2.94	2.92	2.96	2.93	2.98
	MCP	2.92	2.92	2.92	2.94	2.92	2.96	2.93	2.98
10^1	LASSO	2.93	3.05	2.94	3.12	2.95	3.17	2.95	3.19
	SCAD	2.93	2.99	2.94	3.05	2.94	3.11	2.95	3.16
	MCP	2.93	2.99	2.94	3.05	2.94	3.11	2.95	3.16
10^2	LASSO	2.93	3.05	2.94	3.12	2.95	3.17	2.95	3.19
	SCAD	2.92	2.96	2.93	3.02	2.94	3.08	2.94	3.14
	MCP	2.92	2.96	2.93	3.02	2.94	3.08	2.94	3.14
10^3	LASSO	2.93	3.05	2.94	3.14	2.95	3.26	2.96	3.40
	SCAD	2.92	2.96	2.93	3.02	2.94	3.08	2.94	3.14
	MCP	2.93	2.96	2.93	3.02	2.94	3.08	2.94	3.14

Table S21: The result on generalization for $q = 5/3$ and $n = 100$.

θ_0	Method	$r_{\mathrm{nz}} = 0.2$		$r_{\mathrm{nz}} = 0.4$		$r_{\mathrm{nz}} = 0.6$		$r_{\mathrm{nz}} = 0.8$	
		$d = 10$	$d = 100$	$d = 10$	$d = 100$	$d = 10$	$d = 100$	$d = 10$	$d = 100$
10^0	LASSO	10.68	14.07	10.71	14.26	10.72	14.46	10.74	14.65
	SCAD	10.68	14.07	10.71	14.26	10.72	14.47	10.74	14.66
	MCP	10.69	14.09	10.71	14.29	10.72	14.49	10.74	14.69
10^1	LASSO	11.04	17.72	11.23	19.45	11.32	21.00	11.40	22.28
	SCAD	10.97	18.85	11.18	21.89	11.31	22.62	11.41	25.29
	MCP	10.97	19.12	11.19	22.11	11.33	23.14	11.42	24.98
10^2	LASSO	11.32	37.51	11.64	120.93	11.74	245.48	11.78	346.97
	SCAD	11.00	18.13	11.31	38.66	11.51	149.72	11.65	199.80
	MCP	11.00	18.05	11.30	27.55	11.51	107.95	11.66	144.35
10^3	LASSO	11.31	1978.49	11.65	10079	11.77	22466	11.89	32608
	SCAD	10.89	16.41	11.18	1627	11.39	12520	11.60	17490
	MCP	10.90	16.41	11.18	664	11.39	8513	11.61	12066

Table S22: The result on generalization for $q = 5/3$ and $n = 1{,}000$.

θ_0	Method	$r_{\mathrm{nz}} = 0.2$		$r_{\mathrm{nz}} = 0.4$		$r_{\mathrm{nz}} = 0.6$		$r_{\mathrm{nz}} = 0.8$	
		$d = 10$	$d = 100$	$d = 10$	$d = 100$	$d = 10$	$d = 100$	$d = 10$	$d = 100$
10^0	LASSO	25.40	12.63	25.41	12.65	25.41	12.67	25.41	12.69
	SCAD	25.40	12.63	25.41	12.65	25.41	12.67	25.41	12.69
	MCP	25.40	12.63	25.41	12.65	25.41	12.67	25.41	12.69
10^1	LASSO	25.45	13.08	25.48	13.32	25.49	13.49	25.50	13.59
	SCAD	25.45	13.07	25.48	13.34	25.50	13.52	25.50	13.63
	MCP	25.45	13.08	25.47	13.35	25.50	13.53	25.51	13.64
10^2	LASSO	25.48	13.25	25.53	13.57	25.57	13.75	25.58	13.84
	SCAD	25.45	12.90	25.49	13.17	25.54	13.42	25.57	13.66
	MCP	25.45	12.90	25.50	13.18	25.54	13.41	25.57	13.66
10^3	LASSO	25.52	13.25	25.56	13.57	25.58	13.79	25.65	14.00
	SCAD	25.44	12.85	25.47	13.11	25.50	13.37	25.59	13.63
	MCP	25.43	12.85	25.47	13.11	25.50	13.37	25.59	13.63

Table S23: The results on model selection for $q = 1$ (the normal error). Note that, when $q = 1$, L_q-criteria are equivalent to the original criteria, respectively.

Case	Method	Seq	AIC1	AIC2	BIC1	BIC2	L_q-A1	L_q-A2	L_q-B1	L_q-B2	CV
1a	LASSO	71	10	4	32	12	-	-	-	-	7
	SCAD	69	10	7	32	16	-	-	-	-	8
	MCP	67	10	7	32	16	-	-	-	-	11
1b	LASSO	67	8	4	13	3	-	-	-	-	7
	SCAD	68	8	4	13	5	-	-	-	-	5
	MCP	67	9	6	13	8	-	-	-	-	9
2a	LASSO	1000	53	37	366	219	-	-	-	-	125
	SCAD	1000	45	186	340	676	-	-	-	-	710
	MCP	1000	44	121	340	546	-	-	-	-	778
2b	LASSO	1000	39	28	579	401	-	-	-	-	155
	SCAD	1000	38	184	578	823	-	-	-	-	689
	MCP	1000	38	119	578	738	-	-	-	-	750
3a	LASSO	1000	54	34	366	220	-	-	-	-	126
	SCAD	1000	46	185	340	675	-	-	-	-	781
	MCP	1000	46	122	339	544	-	-	-	-	802
3b	LASSO	1000	39	27	577	405	-	-	-	-	155
	SCAD	1000	39	183	577	828	-	-	-	-	733
	MCP	1000	39	121	577	740	-	-	-	-	768
4a	LASSO	1000	75	123	365	293	-	-	-	-	140
	SCAD	1000	57	364	340	738	-	-	-	-	781
	MCP	1000	58	250	339	581	-	-	-	-	808
4b	LASSO	1000	45	119	577	440	-	-	-	-	162
	SCAD	1000	41	356	577	851	-	-	-	-	747
	MCP	1000	41	249	577	756	-	-	-	-	778
5a	LASSO	0	-	0	-	0	-	-	-	-	0
	SCAD	0	-	0	-	0	-	-	-	-	0
	MCP	0	-	0	-	0	-	-	-	-	0
5b	LASSO	0	0	0	0	0	-	-	-	-	0
	SCAD	0	0	0	0	0	-	-	-	-	0
	MCP	0	0	0	0	0	-	-	-	-	0
6a	LASSO	20	-	0	-	0	-	-	-	-	0
	SCAD	752	-	23	-	415	-	-	-	-	171
	MCP	985	-	0	-	79	-	-	-	-	519
6b	LASSO	1000	0	0	28	0	-	-	-	-	0
	SCAD	1000	0	3	6	485	-	-	-	-	107
	MCP	1000	0	0	6	611	-	-	-	-	479
7a	LASSO	58	-	35	-	35	-	-	-	-	35
	SCAD	1000	-	1000	-	1000	-	-	-	-	1000
	MCP	1000	-	1000	-	1000	-	-	-	-	1000
7b	LASSO	1000	0	0	28	0	-	-	-	-	0
	SCAD	1000	0	3	8	833	-	-	-	-	643
	MCP	1000	0	0	8	626	-	-	-	-	753
8a	LASSO	46	-	46	-	46	-	-	-	-	46
	SCAD	1000	-	1000	-	1000	-	-	-	-	1000
	MCP	1000	-	1000	-	1000	-	-	-	-	1000
8b	LASSO	1000	0	0	28	0	-	-	-	-	0
	SCAD	1000	0	2	7	834	-	-	-	-	651
	MCP	1000	0	4	7	631	-	-	-	-	754
9a	LASSO	22	9	2	7	3	-	-	-	-	3
	SCAD	22	9	2	7	5	-	-	-	-	4
	MCP	21	8	4	6	5	-	-	-	-	3
9b	LASSO	15	7	4	1	2	-	-	-	-	2
	SCAD	16	7	3	1	4	-	-	-	-	1
	MCP	18	8	5	1	4	-	-	-	-	1

Case	Method	Seq	AIC1	AIC2	BIC1	BIC2	L_q-A1	L_q-A2	L_q-B1	L_q-B2	CV
10a	LASSO	999	112	24	486	93	-	-	-	-	39
	SCAD	1000	100	239	448	689	-	-	-	-	719
	MCP	1000	100	179	448	593	-	-	-	-	793
10b	LASSO	1000	90	19	670	240	-	-	-	-	52
	SCAD	1000	89	226	672	840	-	-	-	-	713
	MCP	1000	89	178	672	781	-	-	-	-	771
11a	LASSO	1000	111	21	487	92	-	-	-	-	38
	SCAD	1000	100	236	449	691	-	-	-	-	782
	MCP	1000	100	178	449	593	-	-	-	-	812
11b	LASSO	1000	90	20	670	246	-	-	-	-	54
	SCAD	1000	89	227	672	842	-	-	-	-	758
	MCP	1000	89	177	672	778	-	-	-	-	788
12a	LASSO	1000	204	221	496	310	-	-	-	-	170
	SCAD	1000	202	504	452	784	-	-	-	-	809
	MCP	1000	202	412	452	674	-	-	-	-	832
12b	LASSO	1000	125	159	670	345	-	-	-	-	122
	SCAD	1000	122	452	672	878	-	-	-	-	777
	MCP	1000	122	352	672	794	-	-	-	-	812
13a	LASSO	0	-	0	-	0	-	-	-	-	0
	SCAD	0	-	0	-	0	-	-	-	-	0
	MCP	0	-	0	-	0	-	-	-	-	0
13b	LASSO	0	0	0	0	0	-	-	-	-	0
	SCAD	0	0	0	0	0	-	-	-	-	0
	MCP	0	0	0	0	0	-	-	-	-	0
14a	LASSO	0	-	0	-	0	-	-	-	-	0
	SCAD	129	-	61	-	114	-	-	-	-	3
	MCP	311	-	20	-	84	-	-	-	-	18
14b	LASSO	988	0	0	81	0	-	-	-	-	0
	SCAD	995	0	3	16	478	-	-	-	-	98
	MCP	999	0	0	16	630	-	-	-	-	473
15a	LASSO	0	-	0	-	0	-	-	-	-	0
	SCAD	822	-	822	-	822	-	-	-	-	732
	MCP	890	-	890	-	890	-	-	-	-	851
15b	LASSO	1000	0	0	85	0	-	-	-	-	0
	SCAD	1000	0	5	16	847	-	-	-	-	684
	MCP	1000	0	0	16	646	-	-	-	-	751
16a	LASSO	0	-	0	-	0	-	-	-	-	0
	SCAD	817	-	817	-	817	-	-	-	-	719
	MCP	889	-	889	-	889	-	-	-	-	846
16b	LASSO	1000	0	0	85	0	-	-	-	-	0
	SCAD	1000	0	70	17	848	-	-	-	-	691
	MCP	1000	0	18	17	658	-	-	-	-	760
17a	LASSO	20	5	1	2	3	-	-	-	-	1
	SCAD	22	5	5	2	5	-	-	-	-	2
	MCP	23	8	5	1	3	-	-	-	-	1
17b	LASSO	17	5	4	0	1	-	-	-	-	1
	SCAD	15	5	5	0	1	-	-	-	-	1
	MCP	14	5	7	0	1	-	-	-	-	1
18a	LASSO	996	252	38	625	116	-	-	-	-	48
	SCAD	998	232	346	597	735	-	-	-	-	768
	MCP	1000	232	305	596	676	-	-	-	-	797
18b	LASSO	1000	189	27	774	176	-	-	-	-	31
	SCAD	1000	189	301	774	851	-	-	-	-	739
	MCP	1000	189	264	774	820	-	-	-	-	795
19a	LASSO	1000	252	38	627	116	-	-	-	-	47
	SCAD	1000	231	344	599	737	-	-	-	-	808
	MCP	1000	231	306	597	673	-	-	-	-	814
19b	LASSO	1000	188	24	774	173	-	-	-	-	30
	SCAD	1000	187	300	774	853	-	-	-	-	777
	MCP	1000	187	263	774	823	-	-	-	-	803

Case	Method	Seq	AIC1	AIC2	BIC1	BIC2	L_q-A1	L_q-A2	L_q-B1	L_q-B2	CV
20a	LASSO	1000	441	403	655	469	-	-	-	-	401
	SCAD	1000	434	689	604	871	-	-	-	-	862
	MCP	1000	434	623	603	796	-	-	-	-	880
20b	LASSO	1000	239	242	774	384	-	-	-	-	238
	SCAD	1000	231	573	774	902	-	-	-	-	812
	MCP	1000	231	491	774	848	-	-	-	-	835
21a	LASSO	0	-	0	-	0	-	-	-	-	0
	SCAD	0	-	0	-	0	-	-	-	-	0
	MCP	0	-	0	-	0	-	-	-	-	0
21b	LASSO	0	0	0	0	0	-	-	-	-	0
	SCAD	0	0	0	0	0	-	-	-	-	0
	MCP	0	0	0	0	0	-	-	-	-	0
22a	LASSO	0	-	0	-	0	-	-	-	-	0
	SCAD	0	-	0	-	0	-	-	-	-	0
	MCP	0	-	0	-	0	-	-	-	-	0
22b	LASSO	953	0	0	216	0	-	-	-	-	0
	SCAD	989	0	16	79	549	-	-	-	-	171
	MCP	1000	0	5	79	655	-	-	-	-	542
23a	LASSO	0	-	0	-	0	-	-	-	-	0
	SCAD	2	-	2	-	2	-	-	-	-	0
	MCP	8	-	8	-	8	-	-	-	-	0
23b	LASSO	1000	0	0	224	0	-	-	-	-	0
	SCAD	1000	0	17	76	856	-	-	-	-	706
	MCP	1000	0	4	75	654	-	-	-	-	781
24a	LASSO	0	-	0	-	0	-	-	-	-	0
	SCAD	2	-	2	-	2	-	-	-	-	1
	MCP	4	-	4	-	4	-	-	-	-	0
24b	LASSO	1000	6	3	226	3	-	-	-	-	3
	SCAD	1000	16	194	77	873	-	-	-	-	730
	MCP	1000	16	104	77	685	-	-	-	-	791
25a	LASSO	46	2	8	3	2	-	-	-	-	3
	SCAD	50	4	12	2	5	-	-	-	-	1
	MCP	49	3	5	1	2	-	-	-	-	2
25b	LASSO	53	2	8	0	1	-	-	-	-	7
	SCAD	52	2	10	0	1	-	-	-	-	5
	MCP	50	2	12	0	1	-	-	-	-	3
26a	LASSO	993	488	86	784	199	-	-	-	-	66
	SCAD	1000	475	527	775	812	-	-	-	-	796
	MCP	1000	475	506	775	798	-	-	-	-	813
26b	LASSO	1000	462	79	875	263	-	-	-	-	63
	SCAD	1000	461	493	875	892	-	-	-	-	782
	MCP	1000	461	479	875	886	-	-	-	-	803
27a	LASSO	1000	489	97	788	205	-	-	-	-	68
	SCAD	1000	475	526	775	813	-	-	-	-	821
	MCP	1000	475	505	775	797	-	-	-	-	833
27b	LASSO	1000	461	83	875	263	-	-	-	-	63
	SCAD	1000	461	492	875	891	-	-	-	-	802
	MCP	1000	461	479	875	886	-	-	-	-	810
28a	LASSO	1000	684	648	823	669	-	-	-	-	648
	SCAD	1000	699	850	787	945	-	-	-	-	921
	MCP	1000	699	813	787	906	-	-	-	-	938
28b	LASSO	1000	522	518	875	603	-	-	-	-	515
	SCAD	1000	531	756	875	935	-	-	-	-	862
	MCP	1000	531	698	875	906	-	-	-	-	885
29a	LASSO	0	-	0	-	0	-	-	-	-	0
	SCAD	0	-	0	-	0	-	-	-	-	0
	MCP	0	-	0	-	0	-	-	-	-	0
29b	LASSO	0	0	0	0	0	-	-	-	-	0
	SCAD	0	0	0	0	0	-	-	-	-	0
	MCP	0	0	0	0	0	-	-	-	-	0

Case	Method	Seq	AIC1	AIC2	BIC1	BIC2	L_q-A1	L_q-A2	L_q-B1	L_q-B2	CV
30a	LASSO	0	-	0	-	0	-	-	-	-	0
	SCAD	0	-	0	-	0	-	-	-	-	0
	MCP	0	-	0	-	0	-	-	-	-	0
30b	LASSO	900	4	0	417	0	-	-	-	-	0
	SCAD	985	0	71	265	673	-	-	-	-	343
	MCP	999	0	22	266	665	-	-	-	-	654
31a	LASSO	0	-	0	-	0	-	-	-	-	0
	SCAD	0	-	0	-	0	-	-	-	-	0
	MCP	0	-	0	-	0	-	-	-	-	0
31b	LASSO	994	4	0	449	0	-	-	-	-	0
	SCAD	1000	0	72	268	842	-	-	-	-	725
	MCP	1000	0	23	267	668	-	-	-	-	790
32a	LASSO	0	-	0	-	0	-	-	-	-	0
	SCAD	0	-	0	-	0	-	-	-	-	0
	MCP	0	-	0	-	0	-	-	-	-	0
32b	LASSO	995	100	76	466	76	-	-	-	-	76
	SCAD	1000	185	470	285	892	-	-	-	-	774
	MCP	1000	186	374	285	776	-	-	-	-	827

Table S24: The results on model selection for $q = 13/11$ (the t-error with $\nu = 10$).

Case	Method	Seq	AIC1	AIC2	BIC1	BIC2	L_q-A1	L_q-A2	L_q-B1	L_q-B2	CV
1a	LASSO	70	29	16	11	2	0	0	0	0	15
	SCAD	69	29	13	11	3	0	0	0	0	11
	MCP	70	29	17	10	4	0	0	0	0	13
1b	LASSO	71	21	9	2	1	0	0	0	0	9
	SCAD	71	21	9	2	2	0	0	0	0	9
	MCP	72	22	9	2	1	0	0	0	0	11
2a	LASSO	1000	320	175	796	518	0	0	0	0	147
	SCAD	1000	303	580	782	896	0	0	0	0	638
	MCP	1000	303	470	782	869	0	0	0	0	758
2b	LASSO	1000	290	160	936	717	0	0	0	0	152
	SCAD	1000	299	590	935	941	0	0	0	0	630
	MCP	1000	299	495	935	955	0	0	0	0	753
3a	LASSO	1000	323	169	797	518	0	0	0	0	148
	SCAD	1000	303	581	783	924	0	0	0	0	768
	MCP	1000	303	465	783	872	0	0	0	0	808
3b	LASSO	1000	295	159	936	720	0	0	0	0	150
	SCAD	1000	300	587	936	978	0	0	0	0	736
	MCP	1000	300	495	936	961	0	0	0	0	783
4a	LASSO	1000	323	225	797	534	0	0	0	0	147
	SCAD	1000	304	638	783	929	0	0	0	0	777
	MCP	1000	303	495	783	875	0	0	0	0	816
4b	LASSO	1000	295	218	936	723	0	0	0	0	152
	SCAD	1000	296	631	936	977	0	0	0	0	749
	MCP	1000	296	511	936	961	0	0	0	0	797
5a	LASSO	0	-	0	-	0	-	0	-	0	0
	SCAD	0	-	0	-	0	-	0	-	0	0
	MCP	0	-	0	-	0	-	0	-	0	0
5b	LASSO	0	0	0	0	0	0	0	0	0	0
	SCAD	0	0	0	0	0	0	0	0	0	0
	MCP	0	0	0	0	0	0	0	0	0	0
6a	LASSO	17	-	0	-	0	-	0	-	0	0
	SCAD	541	-	173	-	382	-	0	-	0	61
	MCP	914	-	13	-	588	-	0	-	0	333
6b	LASSO	1000	0	0	658	0	0	0	0	0	0
	SCAD	1000	0	126	585	394	0	0	0	0	17
	MCP	1000	0	133	590	792	0	0	0	0	292
7a	LASSO	67	-	32	-	32	-	0	-	0	32
	SCAD	1000	-	1000	-	1000	-	0	-	0	1000
	MCP	1000	-	1000	-	1000	-	0	-	0	1000
7b	LASSO	1000	0	0	657	11	0	0	0	0	0
	SCAD	1000	0	464	590	980	0	0	0	0	694
	MCP	1000	0	139	589	939	0	0	0	0	774
8a	LASSO	60	-	59	-	59	-	0	-	0	59
	SCAD	1000	-	1000	-	1000	-	0	-	0	1000
	MCP	1000	-	1000	-	1000	-	0	-	0	1000
8b	LASSO	1000	0	0	660	10	0	0	0	0	0
	SCAD	1000	0	482	590	977	0	0	0	0	697
	MCP	1000	0	151	590	940	0	0	0	0	779
9a	LASSO	15	4	1	0	0	0	0	0	0	0
	SCAD	17	4	0	0	0	0	0	0	0	3
	MCP	20	2	4	0	0	0	0	0	0	0
9b	LASSO	13	2	2	0	0	0	0	0	0	3
	SCAD	13	2	2	0	0	0	0	0	0	2
	MCP	14	2	1	0	0	0	0	0	0	2

Case	Method	Seq	AIC1	AIC2	BIC1	BIC2	L_q-A1	L_q-A2	L_q-B1	L_q-B2	CV
10a	LASSO	999	413	90	821	308	0	0	0	0	50
	SCAD	1000	399	600	808	890	0	0	0	0	637
	MCP	1000	399	509	808	884	0	0	0	0	749
10b	LASSO	1000	396	96	951	541	0	0	0	0	50
	SCAD	1000	390	622	951	937	0	0	0	0	649
	MCP	1000	391	543	951	958	0	0	0	0	761
11a	LASSO	1000	416	97	821	311	0	0	0	0	54
	SCAD	1000	402	597	809	925	0	0	0	0	779
	MCP	1000	401	507	808	885	0	0	0	0	796
11b	LASSO	1000	397	96	952	533	0	0	0	0	53
	SCAD	1000	393	622	952	981	0	0	0	0	749
	MCP	1000	393	530	952	967	0	0	0	0	786
12a	LASSO	1000	428	240	821	402	0	0	0	0	140
	SCAD	1000	409	692	808	946	0	0	0	0	792
	MCP	1000	408	576	808	897	0	0	0	0	817
12b	LASSO	1000	396	185	952	566	0	0	0	0	86
	SCAD	1000	393	679	952	983	0	0	0	0	774
	MCP	1000	392	584	952	968	0	0	0	0	806
13a	LASSO	0	-	0	-	0	-	0	-	0	0
	SCAD	0	-	0	-	0	-	0	-	0	0
	MCP	0	-	0	-	0	-	0	-	0	0
13b	LASSO	0	0	0	0	0	0	0	0	0	0
	SCAD	0	0	0	0	0	0	0	0	0	0
	MCP	0	0	0	0	0	0	0	0	0	0
14a	LASSO	0	-	0	-	0	-	0	-	0	0
	SCAD	55	-	48	-	55	-	0	-	0	2
	MCP	177	-	23	-	135	-	0	-	0	2
14b	LASSO	983	1	0	774	0	0	0	0	0	0
	SCAD	992	0	122	651	337	0	0	0	0	16
	MCP	999	0	143	649	769	0	0	0	0	254
15a	LASSO	0	-	0	-	0	-	0	-	0	0
	SCAD	822	-	822	-	822	-	0	-	0	727
	MCP	897	-	897	-	897	-	0	-	0	833
15b	LASSO	1000	1	0	786	0	0	0	0	0	0
	SCAD	1000	0	433	653	981	0	0	0	0	700
	MCP	1000	0	154	653	940	0	0	0	0	761
16a	LASSO	0	-	0	-	0	-	0	-	0	0
	SCAD	823	-	823	-	823	-	0	-	0	713
	MCP	886	-	886	-	886	-	0	-	0	834
16b	LASSO	1000	2	0	784	0	0	0	0	0	0
	SCAD	1000	0	474	651	981	0	0	0	0	709
	MCP	1000	0	194	651	944	0	0	0	0	771
17a	LASSO	14	1	1	0	1	0	0	0	0	3
	SCAD	16	1	1	0	1	0	0	0	0	0
	MCP	17	1	3	0	0	0	0	0	0	2
17b	LASSO	14	0	1	0	0	0	0	0	0	2
	SCAD	14	0	1	0	0	0	0	0	0	1
	MCP	15	0	1	0	0	0	0	0	0	1
18a	LASSO	997	552	82	877	242	0	0	0	0	37
	SCAD	998	529	649	873	895	0	0	0	0	694
	MCP	1000	530	591	873	901	0	0	0	0	767
18b	LASSO	1000	529	84	965	402	0	0	0	0	37
	SCAD	1000	529	662	965	952	0	0	0	0	695
	MCP	1000	529	618	965	969	0	0	0	0	772
19a	LASSO	1000	552	83	878	247	0	0	0	0	37
	SCAD	1000	529	655	874	921	0	0	0	0	785
	MCP	1000	529	594	874	902	0	0	0	0	798
19b	LASSO	1000	528	86	965	398	0	0	0	0	38
	SCAD	1000	529	662	965	981	0	0	0	0	771
	MCP	1000	529	613	965	975	0	0	0	0	793

Case	Method	Seq	AIC1	AIC2	BIC1	BIC2	L_q-A1	L_q-A2	L_q-B1	L_q-B2	CV
20a	LASSO	1000	576	361	879	461	0	0	0	0	304
	SCAD	1000	535	783	873	953	0	0	0	0	835
	MCP	1000	536	699	873	921	0	0	0	0	852
20b	LASSO	1000	528	272	965	508	0	0	0	0	199
	SCAD	1000	530	744	965	984	0	0	0	0	804
	MCP	1000	530	661	965	975	0	0	0	0	827
21a	LASSO	0	-	0	-	0	-	0	-	0	0
	SCAD	0	-	0	-	0	-	0	-	0	0
	MCP	0	-	0	-	0	-	0	-	0	0
21b	LASSO	0	0	0	0	0	0	0	0	0	0
	SCAD	0	0	0	0	0	0	0	0	0	0
	MCP	0	0	0	0	0	0	0	0	0	0
22a	LASSO	0	-	0	-	0	-	0	-	0	0
	SCAD	0	-	0	-	0	-	0	-	0	0
	MCP	0	-	0	-	0	-	0	-	0	0
22b	LASSO	920	26	0	768	0	0	0	0	0	0
	SCAD	964	3	206	711	420	0	0	0	0	46
	MCP	998	3	205	720	792	0	0	0	0	335
23a	LASSO	0	-	0	-	0	-	0	-	0	0
	SCAD	1	-	1	-	1	-	0	-	0	0
	MCP	7	-	7	-	7	-	0	-	0	2
23b	LASSO	999	30	0	837	0	0	0	0	0	0
	SCAD	1000	3	451	724	980	0	0	0	0	709
	MCP	1000	3	212	724	947	0	0	0	0	744
24a	LASSO	0	-	0	-	0	-	0	-	0	0
	SCAD	1	-	1	-	1	-	0	-	0	0
	MCP	8	-	8	-	8	-	0	-	0	0
24b	LASSO	999	35	0	838	0	0	0	0	0	0
	SCAD	1000	11	527	725	982	0	0	0	0	726
	MCP	1000	10	303	724	945	0	0	0	0	754
25a	LASSO	38	0	3	0	0	0	0	0	0	3
	SCAD	48	0	2	0	0	0	0	0	0	1
	MCP	49	0	2	0	0	0	0	0	0	1
25b	LASSO	32	0	1	0	0	0	0	0	0	3
	SCAD	32	0	1	0	0	0	0	0	0	4
	MCP	32	0	0	0	0	0	0	0	0	1
26a	LASSO	998	724	168	939	320	0	0	0	0	79
	SCAD	998	717	753	939	939	0	0	0	0	772
	MCP	999	717	742	939	944	0	0	0	0	792
26b	LASSO	1000	723	163	982	445	0	0	0	0	62
	SCAD	1000	724	760	982	963	0	0	0	0	764
	MCP	1000	724	743	982	977	0	0	0	0	806
27a	LASSO	1000	724	168	939	324	0	0	0	0	78
	SCAD	1000	716	756	939	953	0	0	0	0	819
	MCP	1000	716	739	939	944	0	0	0	0	823
27b	LASSO	1000	724	166	982	442	0	0	0	0	61
	SCAD	1000	724	760	982	985	0	0	0	0	794
	MCP	1000	724	743	982	984	0	0	0	0	819
28a	LASSO	1000	768	601	940	657	0	0	0	0	582
	SCAD	1000	738	901	939	975	0	0	0	0	905
	MCP	1000	738	859	939	959	0	0	0	0	921
28b	LASSO	1000	726	515	982	650	0	0	0	0	463
	SCAD	1000	726	860	982	988	0	0	0	0	866
	MCP	1000	726	816	982	984	0	0	0	0	892
29a	LASSO	0	-	0	-	0	-	0	-	0	0
	SCAD	0	-	0	-	0	-	0	-	0	0
	MCP	0	-	0	-	0	-	0	-	0	0
29b	LASSO	0	0	0	0	0	0	0	0	0	0
	SCAD	0	0	0	0	0	0	0	0	0	0
	MCP	0	0	0	0	0	0	0	0	0	0

Case	Method	Seq	AIC1	AIC2	BIC1	BIC2	L_q-A1	L_q-A2	L_q-B1	L_q-B2	CV
30a	LASSO	0	-	0	-	0	-	0	-	0	0
	SCAD	0	-	0	-	0	-	0	-	0	0
	MCP	0	-	0	-	0	-	0	-	0	0
30b	LASSO	849	124	0	785	0	0	0	0	0	0
	SCAD	962	47	357	838	607	0	0	0	0	155
	MCP	997	46	297	846	873	0	0	0	0	482
31a	LASSO	0	-	0	-	0	-	0	-	0	0
	SCAD	0	-	0	-	0	-	0	-	0	0
	MCP	0	-	0	-	0	-	0	-	0	0
31b	LASSO	990	138	0	893	0	0	0	0	0	0
	SCAD	1000	52	492	845	984	0	0	0	0	738
	MCP	1000	50	304	845	956	0	0	0	0	778
32a	LASSO	0	-	0	-	0	-	0	-	0	0
	SCAD	0	-	0	-	0	-	0	-	0	0
	MCP	0	-	0	-	0	-	0	-	0	0
32b	LASSO	990	179	32	894	32	0	0	0	0	32
	SCAD	1000	117	672	845	984	0	0	0	0	764
	MCP	1000	117	496	845	961	0	0	0	0	795

Table S25: The results on model selection for $q = 3/2$ (the t-error with $\nu = 3$).

Case	Method	Seq	AIC1	AIC2	BIC1	BIC2	L_q-A1	L_q-A2	L_q-B1	L_q-B2	CV
1a	LASSO	45	13	13	5	4	3	4	1	0	5
	SCAD	44	13	12	5	5	3	4	1	0	8
	MCP	40	12	16	5	5	3	6	1	0	7
1b	LASSO	34	7	5	1	0	6	1	0	0	4
	SCAD	34	8	4	1	0	6	2	0	0	2
	MCP	34	8	6	1	0	6	2	0	0	2
2a	LASSO	983	556	143	848	405	702	495	946	794	133
	SCAD	983	544	459	845	651	697	681	947	845	344
	MCP	985	545	509	845	783	693	761	945	920	562
2b	LASSO	995	653	109	955	545	631	429	971	895	139
	SCAD	995	649	432	955	686	630	640	971	896	254
	MCP	995	650	529	956	846	630	731	971	954	529
3a	LASSO	1000	572	139	866	412	703	498	952	802	133
	SCAD	1000	560	672	858	942	694	855	948	972	783
	MCP	1000	560	600	858	908	694	794	948	965	794
3b	LASSO	1000	661	106	959	541	631	432	994	907	139
	SCAD	1000	651	703	960	986	631	855	994	997	747
	MCP	1000	651	643	960	975	631	786	994	996	786
4a	LASSO	1000	573	155	865	413	704	496	951	800	138
	SCAD	1000	558	681	859	936	693	859	948	971	782
	MCP	1000	558	596	859	904	693	792	948	965	801
4b	LASSO	1000	652	104	960	543	631	431	994	908	134
	SCAD	1000	652	700	960	984	630	856	994	998	755
	MCP	1000	653	632	960	974	630	783	994	996	798
5a	LASSO	0	-	0	-	0	-	0	-	0	0
	SCAD	0	-	0	-	0	-	0	-	0	0
	MCP	0	-	0	-	0	-	0	-	0	0
5b	LASSO	0	0	0	0	0	0	0	0	0	0
	SCAD	0	0	0	0	0	0	0	0	0	0
	MCP	0	0	0	0	0	0	0	0	0	0
6a	LASSO	4	-	0	-	0	-	0	-	0	0
	SCAD	59	-	11	-	31	-	40	-	51	2
	MCP	325	-	1	-	123	-	200	-	264	29
6b	LASSO	822	330	0	735	0	85	0	718	80	0
	SCAD	826	325	0	726	8	49	10	719	86	0
	MCP	880	347	10	762	105	37	120	738	392	8
7a	LASSO	48	-	16	-	16	-	16	-	16	16
	SCAD	1000	-	990	-	997	-	990	-	994	994
	MCP	1000	-	988	-	994	-	989	-	990	999
7b	LASSO	1000	432	0	883	1	82	1	963	84	0
	SCAD	1000	410	553	854	978	37	846	958	994	686
	MCP	1000	412	361	853	948	37	671	958	989	736
8a	LASSO	42	-	41	-	41	-	41	-	41	41
	SCAD	1000	-	1000	-	1000	-	1000	-	1000	1000
	MCP	1000	-	1000	-	1000	-	1000	-	1000	1000
8b	LASSO	1000	432	0	884	1	82	1	963	78	0
	SCAD	1000	407	559	860	980	38	839	958	995	695
	MCP	1000	406	363	860	953	38	666	958	989	741
9a	LASSO	12	1	0	0	0	0	0	0	0	0
	SCAD	9	1	0	0	0	0	0	0	0	1
	MCP	10	1	0	0	0	0	0	0	0	0
9b	LASSO	6	1	0	0	0	0	0	0	0	0
	SCAD	5	1	0	0	0	0	0	0	0	1
	MCP	5	1	0	0	0	0	0	0	0	0

Case	Method	Seq	AIC1	AIC2	BIC1	BIC2	L_q-A1	L_q-A2	L_q-B1	L_q-B2	CV
10a	LASSO	965	574	56	868	220	753	291	943	617	44
	SCAD	965	559	446	853	614	740	662	941	775	322
	MCP	972	560	497	854	747	739	763	947	908	512
10b	LASSO	997	680	54	967	320	707	257	945	781	51
	SCAD	997	677	393	966	631	705	640	945	861	245
	MCP	997	676	519	966	818	704	746	945	941	481
11a	LASSO	1000	602	53	897	220	752	293	958	618	41
	SCAD	1000	589	658	880	948	737	859	957	971	791
	MCP	1000	588	602	879	918	737	817	957	969	814
11b	LASSO	1000	683	56	970	326	708	255	996	779	54
	SCAD	1000	684	725	969	984	704	877	996	998	782
	MCP	1000	684	673	969	978	704	818	996	997	801
12a	LASSO	1000	607	94	898	251	754	320	958	621	57
	SCAD	1000	592	685	878	949	737	864	957	971	794
	MCP	1000	594	627	878	916	737	816	957	969	824
12b	LASSO	1000	680	69	970	325	708	273	996	778	53
	SCAD	1000	682	727	969	984	704	876	996	998	794
	MCP	1000	683	674	969	977	704	816	996	997	825
13a	LASSO	0	-	0	-	0	-	0	-	0	0
	SCAD	0	-	0	-	0	-	0	-	0	0
	MCP	0	-	0	-	0	-	0	-	0	0
13b	LASSO	0	0	0	0	0	0	0	0	0	0
	SCAD	0	0	0	0	0	0	0	0	0	0
	MCP	0	0	0	0	0	0	0	0	0	0
14a	LASSO	0	-	0	-	0	-	0	-	0	0
	SCAD	4	-	2	-	4	-	4	-	3	0
	MCP	19	-	0	-	9	-	17	-	19	0
14b	LASSO	588	230	0	539	0	165	0	492	14	0
	SCAD	619	217	0	543	4	78	6	513	33	0
	MCP	774	265	7	651	56	71	93	588	276	5
15a	LASSO	0	-	0	-	0	-	0	-	0	0
	SCAD	817	-	816	-	816	-	816	-	816	725
	MCP	888	-	887	-	887	-	887	-	887	828
15b	LASSO	1000	408	0	899	0	191	0	968	18	0
	SCAD	1000	393	532	849	977	66	833	958	993	682
	MCP	1000	390	352	848	939	66	646	958	989	736
16a	LASSO	0	-	0	-	0	-	0	-	0	0
	SCAD	822	-	822	-	822	-	822	-	822	734
	MCP	889	-	889	-	889	-	889	-	889	832
16b	LASSO	1000	410	0	896	0	196	0	967	14	0
	SCAD	1000	395	541	852	976	68	835	958	993	688
	MCP	1000	392	360	851	939	68	658	958	988	744
17a	LASSO	11	0	0	0	0	0	0	0	0	0
	SCAD	12	0	0	0	0	0	0	0	0	2
	MCP	13	0	1	0	0	0	0	0	0	0
17b	LASSO	7	1	0	0	0	0	0	0	0	1
	SCAD	9	2	0	0	0	0	0	0	0	0
	MCP	9	2	0	0	0	0	0	0	0	1
18a	LASSO	956	645	60	890	177	828	255	936	531	32
	SCAD	960	638	512	876	654	816	714	941	806	405
	MCP	967	639	577	878	765	817	809	947	912	585
18b	LASSO	997	734	60	978	265	794	223	921	675	35
	SCAD	997	731	466	978	636	793	684	921	854	316
	MCP	996	732	561	977	812	793	786	919	941	524
19a	LASSO	1000	679	64	926	180	837	259	973	527	35
	SCAD	1000	664	714	909	953	819	900	973	977	805
	MCP	1000	664	683	908	928	819	868	973	976	829
19b	LASSO	1000	737	58	981	257	795	221	996	677	36
	SCAD	1000	735	764	981	985	793	885	996	998	794
	MCP	1000	736	720	981	982	793	854	996	997	801

Case	Method	Seq	AIC1	AIC2	BIC1	BIC2	L_q-A1	L_q-A2	L_q-B1	L_q-B2	CV
20a	LASSO	1000	691	191	926	286	837	336	973	557	129
	SCAD	1000	673	760	909	960	819	905	973	977	836
	MCP	1000	673	711	908	931	819	869	973	976	860
20b	LASSO	1000	738	109	981	296	794	264	996	690	62
	SCAD	1000	737	773	981	985	794	885	996	998	812
	MCP	1000	738	732	981	982	794	855	996	997	834
21a	LASSO	0	-	0	-	0	-	0	-	0	0
	SCAD	0	-	0	-	0	-	0	-	0	0
	MCP	0	-	0	-	0	-	0	-	0	0
21b	LASSO	0	0	0	0	0	0	0	0	0	0
	SCAD	0	0	0	0	0	0	0	0	0	0
	MCP	0	0	0	0	0	0	0	0	0	0
22a	LASSO	0	-	0	-	0	-	0	-	0	0
	SCAD	0	-	0	-	0	-	0	-	0	0
	MCP	0	-	0	-	0	-	0	-	0	0
22b	LASSO	409	177	0	376	0	201	0	337	2	0
	SCAD	467	187	0	421	5	147	11	367	45	0
	MCP	683	265	12	605	57	147	101	440	284	4
23a	LASSO	0	-	0	-	0	-	0	-	0	0
	SCAD	0	-	0	-	0	-	0	-	0	0
	MCP	7	-	7	-	7	-	7	-	7	0
23b	LASSO	999	428	0	909	0	310	0	975	3	0
	SCAD	1000	405	546	861	975	143	838	969	994	702
	MCP	1000	404	387	861	949	143	676	969	984	756
24a	LASSO	0	-	0	-	0	-	0	-	0	0
	SCAD	1	-	1	-	1	-	1	-	1	0
	MCP	4	-	4	-	4	-	4	-	4	1
24b	LASSO	999	432	0	910	0	306	0	975	3	0
	SCAD	1000	410	561	859	978	143	838	969	994	710
	MCP	1000	407	416	859	947	143	694	969	985	764
25a	LASSO	28	0	2	0	0	0	0	0	0	2
	SCAD	26	0	1	0	0	0	0	0	0	0
	MCP	28	0	1	0	0	0	0	0	0	0
25b	LASSO	29	0	0	0	0	0	0	0	0	1
	SCAD	26	0	0	0	0	0	0	0	0	0
	MCP	24	0	0	0	0	0	0	0	0	0
26a	LASSO	949	735	130	891	240	864	289	924	530	76
	SCAD	960	739	619	902	735	874	798	933	853	547
	MCP	967	745	673	908	823	878	861	933	925	649
26b	LASSO	998	810	121	990	325	895	299	905	654	71
	SCAD	998	810	615	990	761	895	788	905	893	512
	MCP	997	809	689	989	875	895	865	903	964	651
27a	LASSO	998	778	135	937	242	883	292	977	532	76
	SCAD	1000	773	771	940	945	880	902	978	979	804
	MCP	1000	773	758	940	938	880	887	978	978	819
27b	LASSO	1000	814	123	992	329	895	298	999	657	72
	SCAD	1000	812	817	992	991	895	913	999	999	808
	MCP	1000	812	805	992	991	895	903	999	999	818
28a	LASSO	999	796	385	939	445	883	494	978	635	352
	SCAD	1000	795	840	940	957	880	910	978	979	872
	MCP	1000	795	811	940	947	880	891	978	978	885
28b	LASSO	1000	819	300	992	450	895	427	999	696	247
	SCAD	1000	817	860	992	991	895	914	999	999	863
	MCP	1000	817	835	992	991	895	903	999	999	860
29a	LASSO	0	-	0	-	0	-	0	-	0	0
	SCAD	0	-	0	-	0	-	0	-	0	0
	MCP	0	-	0	-	0	-	0	-	0	0
29b	LASSO	0	0	0	0	0	0	0	0	0	0
	SCAD	0	0	0	0	0	0	0	0	0	0
	MCP	0	0	0	0	0	0	0	0	0	0

Case	Method	Seq	AIC1	AIC2	BIC1	BIC2	L_q-A1	L_q-A2	L_q-B1	L_q-B2	CV
30a	LASSO	0	-	0	-	0	-	0	-	0	0
	SCAD	0	-	0	-	0	-	0	-	0	0
	MCP	0	-	0	-	0	-	0	-	0	0
30b	LASSO	433	225	0	419	0	308	0	310	2	0
	SCAD	531	255	8	501	32	332	60	357	129	1
	MCP	732	338	56	683	147	371	230	391	426	26
31a	LASSO	0	-	0	-	0	-	0	-	0	0
	SCAD	0	-	0	-	0	-	0	-	0	0
	MCP	0	-	0	-	0	-	0	-	0	0
31b	LASSO	990	497	0	932	0	541	0	973	3	0
	SCAD	1000	452	595	911	970	366	846	980	996	751
	MCP	1000	453	465	911	955	366	728	980	992	806
32a	LASSO	0	-	0	-	0	-	0	-	0	0
	SCAD	0	-	0	-	0	-	0	-	0	0
	MCP	0	-	0	-	0	-	0	-	0	0
32b	LASSO	991	505	0	933	0	544	0	974	2	0
	SCAD	1000	457	624	912	975	365	852	980	996	763
	MCP	1000	459	492	912	956	365	741	980	993	814

Table S26: The results on model selection for $q = 5/3$ (the t-error with $\nu = 2$).

Case	Method	Seq	AIC1	AIC2	BIC1	BIC2	L_q-A1	L_q-A2	L_q-B1	L_q-B2	CV
1a	LASSO	27	4	4	2	1	0	0	4	3	4
	SCAD	27	4	4	2	1	0	0	4	2	5
	MCP	27	4	4	2	2	0	0	4	3	1
1b	LASSO	20	1	2	0	0	0	0	2	2	1
	SCAD	20	1	2	0	0	0	0	2	1	1
	MCP	20	1	1	0	0	0	0	2	2	0
2a	LASSO	852	642	93	779	254	3	2	55	37	104
	SCAD	855	646	236	785	369	3	17	51	114	131
	MCP	865	656	358	791	541	3	10	50	91	316
2b	LASSO	830	747	58	810	293	0	0	25	20	106
	SCAD	830	746	135	810	292	0	2	25	66	101
	MCP	831	748	259	810	479	0	0	25	66	270
3a	LASSO	999	785	91	929	254	3	3	54	37	105
	SCAD	999	788	753	926	953	3	17	50	132	779
	MCP	999	788	719	926	933	3	9	50	92	819
3b	LASSO	998	910	54	981	289	0	0	24	20	110
	SCAD	998	913	822	980	981	0	2	25	105	790
	MCP	998	913	802	980	983	0	0	25	67	825
4a	LASSO	1000	786	96	930	267	3	4	53	42	104
	SCAD	1000	786	750	926	954	3	40	50	149	790
	MCP	1000	787	710	926	929	3	16	50	98	830
4b	LASSO	1000	910	60	983	289	0	0	24	22	111
	SCAD	1000	914	800	981	986	0	3	24	111	799
	MCP	1000	914	779	981	983	0	1	24	70	834
5a	LASSO	0	-	0	-	0	-	0	-	0	0
	SCAD	0	-	0	-	0	-	0	-	0	0
	MCP	0	-	0	-	0	-	0	-	0	0
5b	LASSO	0	0	0	0	0	0	0	0	0	0
	SCAD	0	0	0	0	0	0	0	0	0	0
	MCP	0	0	0	0	0	0	0	0	0	0
6a	LASSO	1	-	0	-	0	-	0	-	0	0
	SCAD	6	-	0	-	1	-	0	-	0	0
	MCP	63	-	0	-	12	-	0	-	1	2
6b	LASSO	87	80	0	87	0	0	0	0	0	0
	SCAD	90	82	0	90	0	0	0	0	0	0
	MCP	107	94	0	106	0	0	0	0	0	0
7a	LASSO	64	-	11	-	11	-	11	-	11	11
	SCAD	992	-	915	-	955	-	859	-	866	939
	MCP	996	-	905	-	946	-	859	-	859	956
7b	LASSO	998	935	0	984	0	0	0	0	0	0
	SCAD	999	949	687	987	969	0	0	0	0	655
	MCP	999	948	631	987	962	0	0	0	0	748
8a	LASSO	57	-	50	-	50	-	50	-	50	50
	SCAD	1000	-	1000	-	1000	-	999	-	999	1000
	MCP	1000	-	1000	-	1000	-	999	-	999	1000
8b	LASSO	1000	937	0	988	0	0	0	0	0	0
	SCAD	1000	948	704	987	989	0	0	0	8	675
	MCP	1000	948	630	987	975	0	0	0	0	762
9a	LASSO	5	0	1	0	0	1	0	0	0	0
	SCAD	6	0	1	0	0	0	0	0	0	1
	MCP	5	0	1	0	0	0	0	0	0	0
9b	LASSO	7	0	0	0	0	1	0	2	1	0
	SCAD	7	0	0	0	0	1	0	2	1	0
	MCP	6	0	0	0	0	1	1	1	1	0

Case	Method	Seq	AIC1	AIC2	BIC1	BIC2	L_q-A1	L_q-A2	L_q-B1	L_q-B2	CV
10a	LASSO	787	597	37	727	129	10	4	81	11	32
	SCAD	785	583	204	728	315	8	26	74	140	106
	MCP	799	595	324	739	455	8	20	74	128	252
10b	LASSO	766	668	28	750	143	3	0	62	10	38
	SCAD	765	673	81	750	187	3	8	61	77	56
	MCP	764	672	183	749	332	3	5	61	93	141
11a	LASSO	999	789	35	935	124	10	4	82	10	28
	SCAD	999	780	751	931	950	8	26	73	176	776
	MCP	999	780	715	931	929	8	20	73	129	822
11b	LASSO	998	901	27	984	143	3	0	63	10	38
	SCAD	998	905	807	984	981	3	8	61	139	793
	MCP	998	905	802	984	982	3	5	61	104	824
12a	LASSO	1000	791	44	933	130	10	9	81	23	29
	SCAD	1000	780	754	933	951	8	61	73	217	798
	MCP	1000	781	707	934	931	8	37	73	143	836
12b	LASSO	1000	901	25	986	147	3	2	62	15	34
	SCAD	1000	906	802	986	989	3	16	61	156	811
	MCP	1000	906	792	986	984	3	9	61	109	835
13a	LASSO	0	-	0	-	0	-	0	-	0	0
	SCAD	0	-	0	-	0	-	0	-	0	0
	MCP	0	-	0	-	0	-	0	-	0	0
13b	LASSO	0	0	0	0	0	0	0	0	0	0
	SCAD	0	0	0	0	0	0	0	0	0	0
	MCP	0	0	0	0	0	0	0	0	0	0
14a	LASSO	0	-	0	-	0	-	0	-	0	0
	SCAD	0	-	0	-	0	-	0	-	0	0
	MCP	0	-	0	-	0	-	0	-	0	0
14b	LASSO	24	19	0	22	0	0	0	0	0	0
	SCAD	28	23	0	27	0	0	0	0	0	0
	MCP	50	41	0	48	0	0	0	0	0	0
15a	LASSO	0	-	0	-	0	-	0	-	0	0
	SCAD	776	-	760	-	770	-	758	-	758	670
	MCP	880	-	854	-	857	-	852	-	852	807
15b	LASSO	997	912	0	984	0	0	0	0	0	0
	SCAD	997	914	640	983	963	0	0	0	0	665
	MCP	997	915	590	982	949	0	0	0	0	745
16a	LASSO	0	-	0	-	0	-	0	-	0	0
	SCAD	815	-	815	-	815	-	815	-	815	708
	MCP	893	-	893	-	893	-	893	-	893	837
16b	LASSO	1000	916	0	987	0	0	0	0	0	0
	SCAD	1000	918	658	983	981	0	0	0	0	684
	MCP	1000	918	598	983	960	0	0	0	0	756
17a	LASSO	6	0	1	0	0	3	2	0	2	1
	SCAD	7	0	1	0	0	3	2	0	2	0
	MCP	8	0	1	0	0	2	3	0	2	0
17b	LASSO	3	0	0	0	0	2	0	0	1	0
	SCAD	4	0	0	0	0	2	1	0	1	0
	MCP	5	0	0	0	0	2	1	0	1	0
18a	LASSO	769	586	42	722	107	50	3	169	19	28
	SCAD	772	587	240	722	327	44	70	160	211	150
	MCP	796	606	337	745	478	44	62	159	210	277
18b	LASSO	743	659	29	729	98	19	1	139	19	27
	SCAD	739	659	97	725	195	19	30	137	134	64
	MCP	744	664	198	731	339	19	27	137	158	164
19a	LASSO	997	778	44	932	109	47	3	170	20	28
	SCAD	998	783	743	934	945	44	69	161	244	782
	MCP	998	783	716	935	931	44	61	161	212	825
19b	LASSO	999	911	27	986	99	19	1	141	21	30
	SCAD	999	916	820	986	985	19	33	139	210	792
	MCP	999	917	815	986	981	19	27	139	182	822

Case	Method	Seq	AIC1	AIC2	BIC1	BIC2	L_q-A1	L_q-A2	L_q-B1	L_q-B2	CV
20a	LASSO	999	782	86	935	147	60	45	171	68	46
	SCAD	1000	785	762	936	949	51	145	160	302	816
	MCP	1000	786	729	937	934	51	107	160	241	848
20b	LASSO	1000	913	34	986	111	19	15	141	31	30
	SCAD	1000	919	825	987	990	19	45	139	225	813
	MCP	1000	919	815	987	984	19	38	139	189	833
21a	LASSO	0	-	0	-	0	-	0	-	0	0
	SCAD	0	-	0	-	0	-	0	-	0	0
	MCP	0	-	0	-	0	-	0	-	0	0
21b	LASSO	0	0	0	0	0	0	0	0	0	0
	SCAD	0	0	0	0	0	0	0	0	0	0
	MCP	0	0	0	0	0	0	0	0	0	0
22a	LASSO	0	-	0	-	0	-	0	-	0	0
	SCAD	0	-	0	-	0	-	0	-	0	0
	MCP	0	-	0	-	0	-	0	-	0	0
22b	LASSO	18	17	0	18	0	0	0	0	0	0
	SCAD	27	19	0	27	0	0	0	0	0	0
	MCP	42	33	0	41	0	0	0	0	0	0
23a	LASSO	0	-	0	-	0	-	0	-	0	0
	SCAD	1	-	1	-	1	-	1	-	1	0
	MCP	9	-	9	-	9	-	9	-	9	0
23b	LASSO	994	867	0	975	0	0	0	0	0	0
	SCAD	995	877	642	977	960	0	0	0	0	685
	MCP	997	880	569	979	944	0	0	0	0	754
24a	LASSO	0	-	0	-	0	-	0	-	0	0
	SCAD	0	-	0	-	0	-	0	-	0	0
	MCP	3	-	3	-	3	-	3	-	3	1
24b	LASSO	1000	876	0	981	0	0	0	0	0	0
	SCAD	1000	887	665	981	977	0	0	0	23	705
	MCP	1000	889	586	981	959	0	0	0	4	770
25a	LASSO	25	0	0	0	0	4	11	1	6	0
	SCAD	28	0	0	0	0	3	9	1	4	1
	MCP	28	0	0	0	0	3	5	1	2	0
25b	LASSO	22	0	0	0	0	4	4	4	4	2
	SCAD	21	0	0	0	0	4	5	4	3	0
	MCP	21	0	0	0	0	4	4	4	2	0
26a	LASSO	796	659	112	762	175	199	37	373	81	67
	SCAD	800	654	377	764	467	198	214	370	377	284
	MCP	821	668	458	779	562	198	205	370	384	399
26b	LASSO	801	721	87	794	182	126	14	333	72	63
	SCAD	805	725	233	798	309	126	140	333	285	160
	MCP	806	726	310	799	452	126	142	332	320	254
27a	LASSO	999	829	116	947	178	204	41	383	80	70
	SCAD	999	827	769	946	952	201	221	378	416	785
	MCP	999	827	763	946	949	201	209	378	400	805
27b	LASSO	999	917	88	994	188	128	15	351	72	64
	SCAD	999	915	849	994	987	128	150	349	382	787
	MCP	999	915	844	994	990	128	146	349	369	825
28a	LASSO	1000	840	263	948	314	255	219	390	251	217
	SCAD	1000	838	825	947	964	225	361	378	501	851
	MCP	1000	838	800	947	959	225	318	378	455	857
28b	LASSO	1000	919	156	995	229	128	122	351	146	111
	SCAD	1000	918	860	995	993	128	201	349	412	832
	MCP	1000	918	849	995	993	128	175	349	375	851
29a	LASSO	0	-	0	-	0	-	0	-	0	0
	SCAD	0	-	0	-	0	-	0	-	0	0
	MCP	0	-	0	-	0	-	0	-	0	0
29b	LASSO	0	0	0	0	0	0	0	0	0	0
	SCAD	0	0	0	0	0	0	0	0	0	0
	MCP	0	0	0	0	0	0	0	0	0	0

Case	Method	Seq	AIC1	AIC2	BIC1	BIC2	L_q-A1	L_q-A2	L_q-B1	L_q-B2	CV
30a	LASSO	0	-	0	-	0	-	0	-	0	0
	SCAD	0	-	0	-	0	-	0	-	0	0
	MCP	0	-	0	-	0	-	0	-	0	0
30b	LASSO	35	22	0	35	0	0	0	1	0	0
	SCAD	30	16	0	28	0	0	0	2	0	0
	MCP	59	41	0	58	1	0	0	1	1	0
31a	LASSO	0	-	0	-	0	-	0	-	0	0
	SCAD	0	-	0	-	0	-	0	-	0	0
	MCP	0	-	0	-	0	-	0	-	0	0
31b	LASSO	980	816	0	956	0	0	0	4	0	0
	SCAD	997	846	624	971	954	0	0	0	33	715
	MCP	998	847	560	972	951	0	0	0	13	783
32a	LASSO	0	-	0	-	0	-	0	-	0	0
	SCAD	0	-	0	-	0	-	0	-	0	0
	MCP	0	-	0	-	0	-	0	-	0	0
32b	LASSO	993	826	0	969	0	0	0	4	0	0
	SCAD	1000	848	656	974	973	0	0	0	56	735
	MCP	1000	847	598	974	959	0	0	0	14	793

Table S27: The results on model selection for $q = 2$ (the Cauchy error).

Case	Method	Seq	AIC1	AIC2	BIC1	BIC2	L_q-A1	L_q-A2	L_q-B1	L_q-B2	CV
1a	LASSO	24	1	3	0	0	0	0	0	0	2
	SCAD	24	1	3	0	0	0	0	0	0	1
	MCP	27	1	3	0	1	0	0	0	0	0
1b	LASSO	22	0	1	0	0	0	0	0	0	0
	SCAD	22	0	1	0	0	0	0	0	0	3
	MCP	22	0	2	0	0	0	0	0	0	1
2a	LASSO	117	81	22	80	34	0	0	0	0	21
	SCAD	117	83	28	81	36	0	0	0	0	28
	MCP	118	83	37	81	52	0	0	0	0	43
2b	LASSO	27	3	5	2	4	0	0	0	0	1
	SCAD	27	3	5	2	4	0	0	0	0	3
	MCP	26	3	3	2	4	0	0	0	0	1
3a	LASSO	845	803	32	821	59	0	0	0	0	39
	SCAD	844	806	491	823	561	0	0	0	0	431
	MCP	844	807	526	824	608	0	0	0	0	546
3b	LASSO	530	518	19	517	26	0	0	0	0	29
	SCAD	531	519	131	518	140	0	0	0	0	89
	MCP	530	518	197	517	214	0	0	0	0	173
4a	LASSO	986	942	37	961	66	0	0	0	0	43
	SCAD	986	948	675	966	807	0	0	0	0	771
	MCP	986	950	675	967	793	0	0	0	0	862
4b	LASSO	941	933	24	934	28	0	0	0	0	28
	SCAD	941	932	728	933	817	0	0	0	0	718
	MCP	941	932	759	933	836	0	0	0	0	774
5a	LASSO	0	-	0	-	0	-	0	-	0	0
	SCAD	0	-	0	-	0	-	0	-	0	0
	MCP	0	-	0	-	0	-	0	-	0	0
5b	LASSO	0	0	0	0	0	0	0	0	0	0
	SCAD	0	0	0	0	0	0	0	0	0	0
	MCP	0	0	0	0	0	0	0	0	0	0
6a	LASSO	0	-	0	-	0	-	0	-	0	0
	SCAD	0	-	0	-	0	-	0	-	0	0
	MCP	0	-	0	-	0	-	0	-	0	0
6b	LASSO	0	0	0	0	0	0	0	0	0	0
	SCAD	0	0	0	0	0	0	0	0	0	0
	MCP	0	0	0	0	0	0	0	0	0	0
7a	LASSO	18	-	0	-	0	-	0	-	0	0
	SCAD	477	-	184	-	314	-	59	-	59	279
	MCP	582	-	157	-	305	-	58	-	59	357
7b	LASSO	221	221	0	221	0	0	0	0	0	0
	SCAD	226	226	1	226	2	0	0	0	0	1
	MCP	235	235	14	235	17	0	0	0	0	20
8a	LASSO	54	-	29	-	29	-	29	-	29	29
	SCAD	949	-	870	-	890	-	839	-	839	915
	MCP	958	-	865	-	878	-	839	-	839	935
8b	LASSO	910	910	0	910	0	0	0	0	0	0
	SCAD	910	909	534	910	689	0	0	0	0	593
	MCP	911	910	564	911	710	0	0	0	0	688
9a	LASSO	8	0	2	0	2	0	0	0	0	2
	SCAD	8	0	2	0	2	0	0	0	0	1
	MCP	7	0	1	0	1	0	0	0	0	0
9b	LASSO	6	0	1	0	1	0	0	0	0	0
	SCAD	6	0	1	0	1	0	0	0	0	0
	MCP	5	0	0	0	0	0	0	0	0	0

Case	Method	Seq	AIC1	AIC2	BIC1	BIC2	L_q-A1	L_q-A2	L_q-B1	L_q-B2	CV
10a	LASSO	67	57	9	57	17	0	0	0	0	4
	SCAD	69	58	12	58	15	0	0	0	0	8
	MCP	69	57	16	57	24	0	0	0	0	12
10b	LASSO	6	0	1	0	0	0	0	0	0	0
	SCAD	6	0	1	0	0	0	0	0	0	0
	MCP	6	0	0	0	0	0	0	0	0	0
11a	LASSO	810	753	12	777	29	0	0	0	0	19
	SCAD	810	762	454	782	543	0	0	0	0	431
	MCP	815	766	493	786	593	0	0	0	0	532
11b	LASSO	478	476	7	476	9	0	0	0	0	13
	SCAD	478	475	85	475	103	0	0	0	0	75
	MCP	476	473	135	473	154	0	0	0	0	144
12a	LASSO	982	909	14	935	32	0	0	0	0	19
	SCAD	983	923	657	944	801	0	0	0	0	777
	MCP	984	924	655	945	796	0	0	0	0	851
12b	LASSO	939	936	7	936	9	0	0	0	0	13
	SCAD	940	938	698	938	815	0	0	0	0	722
	MCP	940	938	721	938	831	0	0	0	0	787
13a	LASSO	0	-	0	-	0	-	0	-	0	0
	SCAD	0	-	0	-	0	-	0	-	0	0
	MCP	0	-	0	-	0	-	0	-	0	0
13b	LASSO	0	0	0	0	0	0	0	0	0	0
	SCAD	0	0	0	0	0	0	0	0	0	0
	MCP	0	0	0	0	0	0	0	0	0	0
14a	LASSO	0	-	0	-	0	-	0	-	0	0
	SCAD	0	-	0	-	0	-	0	-	0	0
	MCP	0	-	0	-	0	-	0	-	0	0
14b	LASSO	0	0	0	0	0	0	0	0	0	0
	SCAD	0	0	0	0	0	0	0	0	0	0
	MCP	0	0	0	0	0	0	0	0	0	0
15a	LASSO	0	-	0	-	0	-	0	-	0	0
	SCAD	255	-	183	-	219	-	150	-	150	136
	MCP	333	-	202	-	229	-	176	-	176	177
15b	LASSO	162	162	0	162	0	0	0	0	0	0
	SCAD	164	162	0	162	2	0	0	0	0	1
	MCP	200	200	15	200	19	0	0	0	0	15
16a	LASSO	0	-	0	-	0	-	0	-	0	0
	SCAD	753	-	742	-	746	-	734	-	734	657
	MCP	836	-	817	-	820	-	811	-	811	761
16b	LASSO	892	881	0	883	0	0	0	0	0	0
	SCAD	895	891	502	892	697	0	0	0	0	608
	MCP	903	898	519	899	685	0	0	0	0	689
17a	LASSO	7	0	0	0	0	0	0	0	0	0
	SCAD	7	0	0	0	0	0	0	0	0	0
	MCP	7	0	0	0	0	0	0	0	0	0
17b	LASSO	4	0	0	0	0	0	0	0	0	0
	SCAD	4	0	0	0	0	0	0	0	0	0
	MCP	4	0	0	0	0	0	0	0	0	0
18a	LASSO	72	53	6	53	9	0	0	0	0	5
	SCAD	68	50	8	49	13	0	0	0	0	3
	MCP	67	50	12	49	21	0	0	0	0	7
18b	LASSO	3	0	1	0	1	0	0	0	0	0
	SCAD	3	0	1	0	0	0	0	0	0	0
	MCP	4	0	0	0	0	0	0	0	0	0
19a	LASSO	807	712	13	745	22	0	0	0	0	14
	SCAD	810	719	440	755	546	0	0	0	0	482
	MCP	811	719	472	756	584	0	0	0	0	547
19b	LASSO	452	450	8	451	11	0	0	0	0	12
	SCAD	455	452	103	453	116	0	0	0	0	102
	MCP	456	454	141	455	167	0	0	0	0	161

Case	Method	Seq	AIC1	AIC2	BIC1	BIC2	L_q-A1	L_q-A2	L_q-B1	L_q-B2	CV
	LASSO	981	858	13	895	28	0	0	0	0	14
20a	SCAD	983	869	641	913	789	0	0	0	0	805
	MCP	983	869	646	913	784	0	0	0	0	853
	LASSO	944	940	10	941	16	0	0	0	0	16
20b	SCAD	944	942	703	942	806	0	0	0	0	738
	MCP	945	943	718	943	817	0	0	0	0	779
	LASSO	0	-	0	-	0	-	0	-	0	0
21a	SCAD	0	-	0	-	0	-	0	-	0	0
	MCP	0	-	0	-	0	-	0	-	0	0
	LASSO	0	0	0	0	0	0	0	0	0	0
21b	SCAD	0	0	0	0	0	0	0	0	0	0
	MCP	0	0	0	0	0	0	0	0	0	0
	LASSO	0	-	0	-	0	-	0	-	0	0
22a	SCAD	0	-	0	-	0	-	0	-	0	0
	MCP	0	-	0	-	0	-	0	-	0	0
	LASSO	0	0	0	0	0	0	0	0	0	0
22b	SCAD	0	0	0	0	0	0	0	0	0	0
	MCP	0	0	0	0	0	0	0	0	0	0
	LASSO	0	-	0	-	0	-	0	-	0	0
23a	SCAD	2	-	2	-	2	-	2	-	2	0
	MCP	0	-	0	-	0	-	0	-	0	0
	LASSO	133	129	0	132	0	0	0	0	0	0
23b	SCAD	143	141	2	142	2	0	0	0	0	2
	MCP	180	180	18	180	23	0	0	0	0	22
	LASSO	0	-	0	-	0	-	0	-	0	0
24a	SCAD	1	-	1	-	1	-	1	-	1	0
	MCP	7	-	7	-	7	-	7	-	7	1
	LASSO	871	835	0	843	0	0	0	0	0	0
24b	SCAD	886	870	460	871	648	0	0	0	0	612
	MCP	897	882	483	884	648	0	0	0	0	694
	LASSO	26	0	0	0	0	1	1	2	0	0
25a	SCAD	23	0	0	0	0	1	2	1	1	0
	MCP	22	0	0	0	0	1	2	1	1	0
	LASSO	34	0	0	0	0	0	0	3	0	0
25b	SCAD	30	0	0	0	0	0	0	3	3	0
	MCP	31	0	0	0	0	0	0	3	3	0
	LASSO	94	58	11	57	16	3	1	10	2	9
26a	SCAD	86	54	13	52	15	3	3	9	8	7
	MCP	88	57	16	54	21	3	3	9	8	11
	LASSO	30	1	0	1	0	0	0	0	0	0
26b	SCAD	30	1	0	1	0	0	0	0	0	0
	MCP	32	1	0	1	0	0	0	0	0	0
	LASSO	804	692	45	732	63	4	1	12	2	40
27a	SCAD	812	696	495	735	585	4	4	11	11	539
	MCP	813	697	525	736	613	4	4	11	11	586
	LASSO	507	483	29	494	34	0	0	3	0	29
27b	SCAD	506	482	184	494	209	0	0	3	3	179
	MCP	508	483	224	495	253	0	0	3	3	234
	LASSO	981	830	58	877	79	20	20	21	20	48
28a	SCAD	984	833	664	879	795	19	22	19	26	830
	MCP	984	833	669	879	789	19	21	19	23	840
	LASSO	947	912	39	926	47	3	3	3	3	32
28b	SCAD	947	910	676	925	795	3	3	3	3	741
	MCP	948	911	682	926	807	3	3	3	3	793
	LASSO	0	-	0	-	0	-	0	-	0	0
29a	SCAD	0	-	0	-	0	-	0	-	0	0
	MCP	0	-	0	-	0	-	0	-	0	0
	LASSO	0	0	0	0	0	0	0	0	0	0
29b	SCAD	0	0	0	0	0	0	0	0	0	0
	MCP	0	0	0	0	0	0	0	0	0	0

Case	Method	Seq	AIC1	AIC2	BIC1	BIC2	L_q-A1	L_q-A2	L_q-B1	L_q-B2	CV
30a	LASSO	0	-	0	-	0	-	0	-	0	0
	SCAD	0	-	0	-	0	-	0	-	0	0
	MCP	0	-	0	-	0	-	0	-	0	0
30b	LASSO	0	0	0	0	0	0	0	0	0	0
	SCAD	0	0	0	0	0	0	0	0	0	0
	MCP	0	0	0	0	0	0	0	0	0	0
31a	LASSO	0	-	0	-	0	-	0	-	0	0
	SCAD	0	-	0	-	0	-	0	-	0	0
	MCP	0	-	0	-	0	-	0	-	0	0
31b	LASSO	129	120	0	123	0	0	0	0	0	0
	SCAD	153	141	10	145	13	0	0	0	0	11
	MCP	187	174	25	178	40	0	0	0	0	33
32a	LASSO	0	-	0	-	0	-	0	-	0	0
	SCAD	0	-	0	-	0	-	0	-	0	0
	MCP	0	-	0	-	0	-	0	-	0	0
32b	LASSO	863	756	0	779	0	0	0	0	0	0
	SCAD	891	822	421	840	611	0	0	0	0	631
	MCP	904	839	437	855	606	0	0	0	0	719

Table S28: The results on model selection for $q = 2.01$.

Case	Method	Seq	AIC1	AIC2	BIC1	BIC2	L_q-A1	L_q-A2	L_q-B1	L_q-B2	CV
1a	LASSO	20	0	1	0	1	0	0	0	0	1
	SCAD	20	0	1	0	1	0	0	0	0	2
	MCP	19	0	1	0	1	0	0	0	0	0
1b	LASSO	14	0	2	0	2	0	0	0	0	1
	SCAD	14	0	2	0	2	0	0	0	0	3
	MCP	14	0	0	0	0	0	0	0	0	1
2a	LASSO	112	84	33	82	45	0	0	0	0	17
	SCAD	113	86	33	81	44	0	0	0	0	24
	MCP	112	87	47	81	52	0	0	0	0	32
2b	LASSO	20	1	6	1	6	0	0	0	0	3
	SCAD	20	1	6	1	6	0	0	0	0	2
	MCP	22	1	1	1	2	0	0	0	0	2
3a	LASSO	846	826	57	834	124	0	0	0	0	51
	SCAD	845	828	556	835	604	0	0	0	0	450
	MCP	846	827	616	834	677	0	0	0	0	546
3b	LASSO	502	493	17	492	30	0	0	0	0	41
	SCAD	502	493	135	492	143	0	0	0	0	98
	MCP	504	495	205	494	212	0	0	0	0	182
4a	LASSO	985	962	62	971	122	0	0	0	0	51
	SCAD	985	964	821	972	913	0	0	0	0	816
	MCP	984	965	819	973	904	0	0	0	0	845
4b	LASSO	932	931	24	931	36	0	0	0	0	46
	SCAD	932	931	775	931	838	0	0	0	0	686
	MCP	932	931	792	931	855	0	0	0	0	766
5a	LASSO	0	-	0	-	0	-	0	-	0	0
	SCAD	0	-	0	-	0	-	0	-	0	0
	MCP	0	-	0	-	0	-	0	-	0	0
5b	LASSO	0	0	0	0	0	0	0	0	0	0
	SCAD	0	0	0	0	0	0	0	0	0	0
	MCP	0	0	0	0	0	0	0	0	0	0
6a	LASSO	0	-	0	-	0	-	0	-	0	0
	SCAD	0	-	0	-	0	-	0	-	0	0
	MCP	0	-	0	-	0	-	0	-	0	0
6b	LASSO	0	0	0	0	0	0	0	0	0	0
	SCAD	0	0	0	0	0	0	0	0	0	0
	MCP	0	0	0	0	0	0	0	0	0	0
7a	LASSO	14	-	0	-	1	-	0	-	0	0
	SCAD	421	-	256	-	338	-	38	-	38	229
	MCP	521	-	242	-	377	-	38	-	38	315
7b	LASSO	161	161	0	161	0	0	0	0	0	0
	SCAD	165	165	2	165	3	0	0	0	0	1
	MCP	174	174	10	174	10	0	0	0	0	12
8a	LASSO	49	-	22	-	22	-	22	-	22	22
	SCAD	943	-	870	-	902	-	825	-	825	894
	MCP	960	-	869	-	901	-	825	-	825	916
8b	LASSO	871	871	0	871	0	0	0	0	0	0
	SCAD	872	872	541	872	674	0	0	0	0	551
	MCP	875	875	565	875	708	0	0	0	0	642
9a	LASSO	5	0	0	0	0	0	0	0	0	0
	SCAD	3	0	0	0	0	0	0	0	0	0
	MCP	3	0	0	0	0	0	0	0	0	0
9b	LASSO	1	0	0	0	0	0	0	0	0	0
	SCAD	1	0	0	0	0	0	0	0	0	0
	MCP	1	0	0	0	0	0	0	0	0	0

Case	Method	Seq	AIC1	AIC2	BIC1	BIC2	L_q-A1	L_q-A2	L_q-B1	L_q-B2	CV
10a	LASSO	56	44	7	43	24	0	0	0	0	9
	SCAD	55	45	17	43	22	0	0	0	0	9
	MCP	54	46	20	44	24	0	0	0	0	9
10b	LASSO	6	0	1	0	1	0	0	0	0	0
	SCAD	6	0	1	0	1	0	0	0	0	1
	MCP	6	0	0	0	0	0	0	0	0	0
11a	LASSO	809	769	27	793	72	0	0	0	0	14
	SCAD	809	771	517	792	566	0	0	0	0	433
	MCP	815	779	574	799	641	0	0	0	0	528
11b	LASSO	453	447	8	447	10	0	0	0	0	9
	SCAD	451	445	102	445	107	0	0	0	0	79
	MCP	448	443	141	443	155	0	0	0	0	141
12a	LASSO	984	937	29	961	74	0	0	0	0	14
	SCAD	983	941	783	961	889	0	0	0	0	805
	MCP	983	940	782	960	890	0	0	0	0	849
12b	LASSO	928	928	8	928	9	0	0	0	0	12
	SCAD	929	929	759	929	818	0	0	0	0	691
	MCP	929	929	778	929	837	0	0	0	0	745
13a	LASSO	0	-	0	-	0	-	0	-	0	0
	SCAD	0	-	0	-	0	-	0	-	0	0
	MCP	0	-	0	-	0	-	0	-	0	0
13b	LASSO	0	0	0	0	0	0	0	0	0	0
	SCAD	0	0	0	0	0	0	0	0	0	0
	MCP	0	0	0	0	0	0	0	0	0	0
14a	LASSO	0	-	0	-	0	-	0	-	0	0
	SCAD	0	-	0	-	0	-	0	-	0	0
	MCP	0	-	0	-	0	-	0	-	0	0
14b	LASSO	0	0	0	0	0	0	0	0	0	0
	SCAD	0	0	0	0	0	0	0	0	0	0
	MCP	0	0	0	0	0	0	0	0	0	0
15a	LASSO	0	-	0	-	0	-	0	-	0	0
	SCAD	222	-	177	-	210	-	112	-	113	96
	MCP	275	-	176	-	232	-	124	-	126	125
15b	LASSO	125	124	0	124	0	0	0	0	0	0
	SCAD	123	123	0	123	2	0	0	0	0	1
	MCP	144	144	9	144	12	0	0	0	0	11
16a	LASSO	0	-	0	-	0	-	0	-	0	0
	SCAD	739	-	735	-	737	-	725	-	725	640
	MCP	818	-	807	-	810	-	798	-	798	757
16b	LASSO	842	838	0	839	0	0	0	0	0	0
	SCAD	848	845	526	846	668	0	0	0	0	556
	MCP	863	860	540	861	691	0	0	0	0	645
17a	LASSO	4	0	0	0	0	0	0	0	0	0
	SCAD	5	0	0	0	0	0	0	0	0	0
	MCP	5	0	0	0	0	0	0	0	0	0
17b	LASSO	3	0	0	0	0	0	0	0	0	0
	SCAD	2	0	0	0	0	0	0	0	0	0
	MCP	1	0	0	0	0	0	0	0	0	0
18a	LASSO	51	36	6	37	10	0	0	0	0	10
	SCAD	56	39	5	40	10	0	0	0	0	5
	MCP	60	43	10	43	17	0	0	0	0	7
18b	LASSO	4	1	0	1	0	0	0	0	0	0
	SCAD	4	1	0	1	0	0	0	0	0	0
	MCP	5	1	0	1	0	0	0	0	0	0
19a	LASSO	798	732	31	775	57	0	0	0	0	17
	SCAD	797	740	515	774	584	0	0	0	0	475
	MCP	810	754	565	788	651	0	0	0	0	556
19b	LASSO	448	441	12	441	19	0	0	0	0	12
	SCAD	449	442	123	442	133	0	0	0	0	113
	MCP	453	445	163	445	179	0	0	0	0	154

Case	Method	Seq	AIC1	AIC2	BIC1	BIC2	L_q-A1	L_q-A2	L_q-B1	L_q-B2	CV
20a	LASSO	977	891	31	937	60	0	0	0	0	17
	SCAD	975	896	766	936	883	1	1	1	2	808
	MCP	979	898	747	939	886	1	1	1	2	859
20b	LASSO	934	931	16	933	25	0	0	0	0	11
	SCAD	936	932	730	935	828	0	0	0	0	702
	MCP	936	932	748	935	843	0	0	0	0	766
21a	LASSO	0	-	0	-	0	-	0	-	0	0
	SCAD	0	-	0	-	0	-	0	-	0	0
	MCP	0	-	0	-	0	-	0	-	0	0
21b	LASSO	0	0	0	0	0	0	0	0	0	0
	SCAD	0	0	0	0	0	0	0	0	0	0
	MCP	0	0	0	0	0	0	0	0	0	0
22a	LASSO	0	-	0	-	0	-	0	-	0	0
	SCAD	0	-	0	-	0	-	0	-	0	0
	MCP	0	-	0	-	0	-	0	-	0	0
22b	LASSO	0	0	0	0	0	0	0	0	0	0
	SCAD	0	0	0	0	0	0	0	0	0	0
	MCP	0	0	0	0	0	0	0	0	0	0
23a	LASSO	0	-	0	-	0	-	0	-	0	0
	SCAD	0	-	0	-	0	-	0	-	0	0
	MCP	3	-	3	-	3	-	3	-	3	0
23b	LASSO	93	89	0	92	0	0	0	0	0	0
	SCAD	94	92	3	94	5	0	0	0	0	2
	MCP	134	131	13	134	15	0	0	0	0	12
24a	LASSO	0	-	0	-	0	-	0	-	0	0
	SCAD	1	-	1	-	1	-	1	-	1	0
	MCP	3	-	3	-	3	-	3	-	3	0
24b	LASSO	833	790	0	808	0	0	0	0	0	0
	SCAD	838	819	494	826	668	0	0	0	0	554
	MCP	856	838	506	844	671	0	0	0	0	651
25a	LASSO	18	0	0	0	0	0	0	1	0	0
	SCAD	20	0	0	0	0	0	0	1	0	0
	MCP	22	0	0	0	0	0	0	1	0	0
25b	LASSO	16	0	0	0	0	0	0	1	0	0
	SCAD	15	0	0	0	0	0	0	1	1	0
	MCP	15	0	0	0	0	0	0	1	1	0
26a	LASSO	83	48	15	44	17	4	1	7	2	10
	SCAD	80	49	16	42	17	4	5	7	7	10
	MCP	81	52	21	43	24	4	4	7	7	9
26b	LASSO	27	1	1	1	0	0	0	3	0	1
	SCAD	25	1	0	1	0	0	0	3	2	1
	MCP	25	1	0	1	1	0	0	3	2	1
27a	LASSO	811	716	76	773	116	4	1	7	2	38
	SCAD	811	716	550	772	637	4	5	7	7	524
	MCP	815	717	581	776	687	4	4	7	7	577
27b	LASSO	479	461	35	466	46	0	0	3	0	24
	SCAD	479	459	178	464	197	0	0	3	3	165
	MCP	478	458	220	463	244	0	0	3	3	204
28a	LASSO	976	848	88	921	132	10	10	10	10	45
	SCAD	981	857	755	927	873	10	17	10	23	834
	MCP	984	860	744	930	872	10	17	10	20	852
28b	LASSO	940	905	50	922	65	2	2	3	2	27
	SCAD	938	902	718	920	827	1	2	3	3	735
	MCP	939	903	724	921	840	1	2	3	3	763
29a	LASSO	0	-	0	-	0	-	0	-	0	0
	SCAD	0	-	0	-	0	-	0	-	0	0
	MCP	0	-	0	-	0	-	0	-	0	0
29b	LASSO	0	0	0	0	0	0	0	0	0	0
	SCAD	0	0	0	0	0	0	0	0	0	0
	MCP	0	0	0	0	0	0	0	0	0	0

Case	Method	Seq	AIC1	AIC2	BIC1	BIC2	L_q-A1	L_q-A2	L_q-B1	L_q-B2	CV
30a	LASSO	0	-	0	-	0	-	0	-	0	0
	SCAD	0	-	0	-	0	-	0	-	0	0
	MCP	0	-	0	-	0	-	0	-	0	0
30b	LASSO	0	0	0	0	0	0	0	0	0	0
	SCAD	0	0	0	0	0	0	0	0	0	0
	MCP	0	0	0	0	0	0	0	0	0	0
31a	LASSO	0	-	0	-	0	-	0	-	0	0
	SCAD	0	-	0	-	0	-	0	-	0	0
	MCP	0	-	0	-	0	-	0	-	0	0
31b	LASSO	88	78	0	85	0	0	0	0	0	0
	SCAD	110	102	3	107	7	0	0	0	0	3
	MCP	148	138	19	143	26	0	0	0	0	16
32a	LASSO	0	-	0	-	0	-	0	-	0	0
	SCAD	0	-	0	-	0	-	0	-	0	0
	MCP	0	-	0	-	0	-	0	-	0	0
32b	LASSO	825	719	0	757	0	0	0	0	0	0
	SCAD	850	784	489	815	669	0	0	0	0	564
	MCP	864	797	495	827	679	0	0	0	0	673

Table S29: The results on model selection for $q = 2.1$.

Case	Method	Seq	AIC1	AIC2	BIC1	BIC2	L_q-A1	L_q-A2	L_q-B1	L_q-B2	CV
1a	LASSO	24	0	2	0	2	0	0	0	0	0
	SCAD	24	0	2	0	2	0	0	0	0	2
	MCP	24	0	3	0	2	0	0	0	0	0
1b	LASSO	16	0	6	0	5	0	0	0	0	1
	SCAD	16	0	6	0	5	0	0	0	0	2
	MCP	17	0	4	0	3	0	0	0	0	1
2a	LASSO	47	17	14	13	15	0	0	0	0	9
	SCAD	45	17	13	13	12	0	0	0	0	13
	MCP	44	15	15	11	13	0	0	0	0	6
2b	LASSO	17	0	4	0	4	0	0	0	0	2
	SCAD	17	0	4	0	4	0	0	0	0	2
	MCP	17	0	4	0	3	0	0	0	0	1
3a	LASSO	636	608	46	619	81	0	0	0	0	51
	SCAD	637	608	271	618	299	0	0	0	0	201
	MCP	639	611	343	621	385	0	0	0	0	285
3b	LASSO	142	118	11	118	13	0	0	0	0	16
	SCAD	142	118	24	118	25	0	0	0	0	16
	MCP	143	119	34	119	39	0	0	0	0	27
4a	LASSO	930	891	51	903	97	0	0	0	0	52
	SCAD	929	891	676	903	786	0	0	0	0	723
	MCP	930	892	696	904	789	0	0	0	0	783
4b	LASSO	772	759	22	759	27	0	0	0	0	29
	SCAD	771	758	427	758	476	0	0	0	0	382
	MCP	771	758	482	758	531	0	0	0	0	472
5a	LASSO	0	-	0	-	0	-	0	-	0	0
	SCAD	0	-	0	-	0	-	0	-	0	0
	MCP	0	-	0	-	0	-	0	-	0	0
5b	LASSO	0	0	0	0	0	0	0	0	0	0
	SCAD	0	0	0	0	0	0	0	0	0	0
	MCP	0	0	0	0	0	0	0	0	0	0
6a	LASSO	0	-	0	-	0	-	0	-	0	0
	SCAD	0	-	0	-	0	-	0	-	0	0
	MCP	0	-	0	-	0	-	0	-	0	0
6b	LASSO	0	0	0	0	0	0	0	0	0	0
	SCAD	0	0	0	0	0	0	0	0	0	0
	MCP	0	0	0	0	0	0	0	0	0	0
7a	LASSO	3	-	0	-	0	-	0	-	0	0
	SCAD	154	-	83	-	117	-	1	-	1	64
	MCP	222	-	75	-	139	-	1	-	1	114
7b	LASSO	3	3	0	3	0	0	0	0	0	0
	SCAD	3	3	0	3	0	0	0	0	0	0
	MCP	4	4	0	4	0	0	0	0	0	0
8a	LASSO	42	-	9	-	9	-	9	-	9	9
	SCAD	818	-	691	-	753	-	563	-	563	721
	MCP	850	-	671	-	736	-	563	-	563	778
8b	LASSO	556	556	0	556	0	0	0	0	0	0
	SCAD	558	558	205	558	264	0	0	0	0	219
	MCP	560	560	239	560	309	0	0	0	0	300
9a	LASSO	2	0	0	0	0	0	0	0	0	0
	SCAD	2	0	0	0	0	0	0	0	0	0
	MCP	2	0	0	0	0	0	0	0	0	0
9b	LASSO	3	0	1	0	0	0	0	0	0	0
	SCAD	3	0	1	0	0	0	0	0	0	0
	MCP	3	0	1	0	0	0	0	0	0	0

Case	Method	Seq	AIC1	AIC2	BIC1	BIC2	L_q-A1	L_q-A2	L_q-B1	L_q-B2	CV
10a	LASSO	19	9	5	7	5	0	0	0	0	2
	SCAD	20	9	7	7	4	0	0	0	0	3
	MCP	21	10	6	8	2	0	0	0	0	4
10b	LASSO	2	0	0	0	0	0	0	0	0	0
	SCAD	3	0	0	0	0	0	0	0	0	0
	MCP	3	0	0	0	0	0	0	0	0	0
11a	LASSO	570	528	17	553	44	0	0	0	0	14
	SCAD	569	531	248	554	282	0	0	0	0	192
	MCP	579	541	298	564	351	0	0	0	0	270
11b	LASSO	85	82	0	82	3	0	0	0	0	4
	SCAD	87	84	15	84	15	0	0	0	0	8
	MCP	84	81	21	81	21	0	0	0	0	14
12a	LASSO	922	852	21	881	47	0	0	0	0	14
	SCAD	920	861	649	890	758	0	0	0	0	720
	MCP	923	862	640	892	769	0	0	0	0	783
12b	LASSO	746	743	6	744	11	0	0	0	0	16
	SCAD	743	741	405	741	458	0	0	0	0	394
	MCP	744	743	446	743	501	0	0	0	0	461
13a	LASSO	0	-	0	-	0	-	0	-	0	0
	SCAD	0	-	0	-	0	-	0	-	0	0
	MCP	0	-	0	-	0	-	0	-	0	0
13b	LASSO	0	0	0	0	0	0	0	0	0	0
	SCAD	0	0	0	0	0	0	0	0	0	0
	MCP	0	0	0	0	0	0	0	0	0	0
14a	LASSO	0	-	0	-	0	-	0	-	0	0
	SCAD	0	-	0	-	0	-	0	-	0	0
	MCP	0	-	0	-	0	-	0	-	0	0
14b	LASSO	0	0	0	0	0	0	0	0	0	0
	SCAD	0	0	0	0	0	0	0	0	0	0
	MCP	0	0	0	0	0	0	0	0	0	0
15a	LASSO	0	-	0	-	0	-	0	-	0	0
	SCAD	55	-	46	-	53	-	23	-	23	20
	MCP	75	-	40	-	58	-	26	-	26	27
15b	LASSO	1	1	0	1	0	0	0	0	0	0
	SCAD	2	2	0	2	0	0	0	0	0	0
	MCP	3	3	0	3	0	0	0	0	0	0
16a	LASSO	0	-	0	-	0	-	0	-	0	0
	SCAD	622	-	598	-	612	-	558	-	558	498
	MCP	695	-	637	-	652	-	608	-	608	587
16b	LASSO	522	513	0	514	0	0	0	0	0	0
	SCAD	529	525	196	526	274	0	0	0	0	231
	MCP	545	543	228	544	311	0	0	0	0	295
17a	LASSO	4	0	0	0	0	0	0	0	0	0
	SCAD	4	0	0	0	0	0	0	0	0	0
	MCP	5	0	0	0	0	0	0	0	0	0
17b	LASSO	6	0	0	0	0	0	0	0	0	0
	SCAD	6	0	0	0	0	0	0	0	0	0
	MCP	5	0	0	0	0	0	0	0	0	0
18a	LASSO	12	3	0	4	0	0	0	0	0	1
	SCAD	11	3	0	4	0	0	0	0	0	0
	MCP	13	4	0	5	2	0	0	0	0	2
18b	LASSO	6	0	0	0	0	0	0	0	0	0
	SCAD	5	0	0	0	0	0	0	0	0	0
	MCP	5	0	0	0	0	0	0	0	0	0
19a	LASSO	548	490	21	519	38	0	0	0	0	10
	SCAD	557	500	247	525	292	0	0	0	0	221
	MCP	565	511	293	537	357	0	0	0	0	284
19b	LASSO	95	91	4	92	7	0	0	0	0	5
	SCAD	94	88	7	89	9	0	0	0	0	11
	MCP	95	89	15	90	17	0	0	0	0	14

Case	Method	Seq	AIC1	AIC2	BIC1	BIC2	L_q-A1	L_q-A2	L_q-B1	L_q-B2	CV
20a	LASSO	921	815	26	864	46	0	0	0	0	14
	SCAD	918	827	629	866	752	0	0	0	0	720
	MCP	922	833	621	871	757	0	0	0	0	776
20b	LASSO	745	728	11	734	14	0	0	0	0	13
	SCAD	743	728	384	732	471	0	0	0	0	429
	MCP	745	730	423	734	518	0	0	0	0	480
21a	LASSO	0	-	0	-	0	-	0	-	0	0
	SCAD	0	-	0	-	0	-	0	-	0	0
	MCP	0	-	0	-	0	-	0	-	0	0
21b	LASSO	0	0	0	0	0	0	0	0	0	0
	SCAD	0	0	0	0	0	0	0	0	0	0
	MCP	0	0	0	0	0	0	0	0	0	0
22a	LASSO	0	-	0	-	0	-	0	-	0	0
	SCAD	0	-	0	-	0	-	0	-	0	0
	MCP	0	-	0	-	0	-	0	-	0	0
22b	LASSO	0	0	0	0	0	0	0	0	0	0
	SCAD	0	0	0	0	0	0	0	0	0	0
	MCP	0	0	0	0	0	0	0	0	0	0
23a	LASSO	0	-	0	-	0	-	0	-	0	0
	SCAD	0	-	0	-	0	-	0	-	0	0
	MCP	0	-	0	-	0	-	0	-	0	0
23b	LASSO	0	0	0	0	0	0	0	0	0	0
	SCAD	0	0	0	0	0	0	0	0	0	0
	MCP	1	1	0	1	0	0	0	0	0	0
24a	LASSO	0	-	0	-	0	-	0	-	0	0
	SCAD	1	-	1	-	1	-	1	-	1	0
	MCP	3	-	3	-	3	-	3	-	3	0
24b	LASSO	493	464	0	474	0	0	0	0	0	0
	SCAD	510	489	172	498	272	0	0	0	0	224
	MCP	536	510	195	518	300	0	0	0	0	295
25a	LASSO	20	0	0	0	0	1	0	3	0	0
	SCAD	20	0	0	0	0	0	1	3	2	0
	MCP	20	0	0	0	0	0	1	3	2	0
25b	LASSO	14	0	0	0	0	0	0	0	0	0
	SCAD	13	0	0	0	0	0	0	0	0	0
	MCP	13	0	0	0	0	0	0	0	0	0
26a	LASSO	32	6	2	4	3	0	0	3	0	4
	SCAD	32	4	3	3	2	0	0	3	2	0
	MCP	29	5	3	4	4	0	0	3	2	0
26b	LASSO	17	0	0	0	0	0	0	0	0	0
	SCAD	15	0	0	0	0	0	0	0	0	0
	MCP	15	0	0	0	0	0	0	0	0	0
27a	LASSO	582	502	52	545	79	1	0	5	0	39
	SCAD	581	498	298	535	360	1	2	5	5	288
	MCP	592	507	333	545	413	1	2	5	5	339
27b	LASSO	132	103	11	106	15	0	0	0	0	16
	SCAD	132	102	15	105	17	0	0	0	0	19
	MCP	132	102	24	105	27	0	0	0	0	22
28a	LASSO	924	780	72	854	108	8	8	9	8	41
	SCAD	925	777	594	846	753	8	8	8	9	741
	MCP	926	778	597	847	757	8	8	8	9	786
28b	LASSO	758	721	33	732	39	0	0	0	0	19
	SCAD	759	720	404	730	500	0	0	0	0	462
	MCP	761	721	433	731	536	0	0	0	0	519
29a	LASSO	0	-	0	-	0	-	0	-	0	0
	SCAD	0	-	0	-	0	-	0	-	0	0
	MCP	0	-	0	-	0	-	0	-	0	0
29b	LASSO	0	0	0	0	0	0	0	0	0	0
	SCAD	0	0	0	0	0	0	0	0	0	0
	MCP	0	0	0	0	0	0	0	0	0	0

Case	Method	Seq	AIC1	AIC2	BIC1	BIC2	L_q-A1	L_q-A2	L_q-B1	L_q-B2	CV
30a	LASSO	0	-	0	-	0	-	0	-	0	0
	SCAD	0	-	0	-	0	-	0	-	0	0
	MCP	0	-	0	-	0	-	0	-	0	0
30b	LASSO	0	0	0	0	0	0	0	0	0	0
	SCAD	0	0	0	0	0	0	0	0	0	0
	MCP	0	0	0	0	0	0	0	0	0	0
31a	LASSO	0	-	0	-	0	-	0	-	0	0
	SCAD	0	-	0	-	0	-	0	-	0	0
	MCP	0	-	0	-	0	-	0	-	0	0
31b	LASSO	0	0	0	0	0	0	0	0	0	0
	SCAD	0	0	0	0	0	0	0	0	0	0
	MCP	1	1	0	1	0	0	0	0	0	0
32a	LASSO	0	-	0	-	0	-	0	-	0	0
	SCAD	0	-	0	-	0	-	0	-	0	0
	MCP	0	-	0	-	0	-	0	-	0	0
32b	LASSO	477	391	0	422	0	0	0	0	0	0
	SCAD	513	462	195	472	299	0	0	0	0	262
	MCP	551	504	220	513	319	0	0	0	0	322

Table S30: The results on model selection for $q = 2.5$.

Case	Method	Seq	AIC1	AIC2	BIC1	BIC2	L_q-A1	L_q-A2	L_q-B1	L_q-B2	CV
1a	LASSO	25	1	8	0	8	0	0	0	0	0
	SCAD	25	1	8	0	8	0	0	0	0	0
	MCP	25	1	6	0	6	0	0	0	0	0
1b	LASSO	20	1	7	1	8	0	0	0	0	1
	SCAD	20	1	8	1	9	0	0	0	0	0
	MCP	21	1	12	1	11	0	0	0	0	0
2a	LASSO	25	1	7	0	7	0	0	0	0	0
	SCAD	25	1	7	0	7	0	0	0	0	0
	MCP	25	1	6	0	6	0	0	0	0	0
2b	LASSO	20	1	7	1	8	0	0	0	0	1
	SCAD	20	1	8	1	9	0	0	0	0	0
	MCP	21	1	12	1	11	0	0	0	0	0
3a	LASSO	25	1	7	0	7	0	0	0	0	0
	SCAD	25	1	7	0	7	0	0	0	0	0
	MCP	26	1	6	0	6	0	0	0	0	0
3b	LASSO	20	1	7	1	8	0	0	0	0	1
	SCAD	20	1	8	1	9	0	0	0	0	0
	MCP	21	1	12	1	11	0	0	0	0	0
4a	LASSO	32	10	7	9	7	0	0	0	0	3
	SCAD	32	10	7	9	7	0	0	0	0	1
	MCP	32	9	9	8	10	0	0	0	0	4
4b	LASSO	20	1	7	1	8	0	0	0	0	1
	SCAD	20	1	8	1	9	0	0	0	0	0
	MCP	21	1	12	1	12	0	0	0	0	0
5a	LASSO	0	-	0	-	0	-	0	-	0	0
	SCAD	0	-	0	-	0	-	0	-	0	0
	MCP	0	-	0	-	0	-	0	-	0	0
5b	LASSO	0	0	0	0	0	0	0	0	0	0
	SCAD	0	0	0	0	0	0	0	0	0	0
	MCP	0	0	0	0	0	0	0	0	0	0
6a	LASSO	0	-	0	-	0	-	0	-	0	0
	SCAD	0	-	0	-	0	-	0	-	0	0
	MCP	0	-	0	-	0	-	0	-	0	0
6b	LASSO	0	0	0	0	0	0	0	0	0	0
	SCAD	0	0	0	0	0	0	0	0	0	0
	MCP	0	0	0	0	0	0	0	0	0	0
7a	LASSO	0	-	0	-	0	-	0	-	0	0
	SCAD	0	-	0	-	0	-	0	-	0	0
	MCP	0	-	0	-	0	-	0	-	0	0
7b	LASSO	0	0	0	0	0	0	0	0	0	0
	SCAD	0	0	0	0	0	0	0	0	0	0
	MCP	0	0	0	0	0	0	0	0	0	0
8a	LASSO	0	-	0	-	0	-	0	-	0	0
	SCAD	0	-	0	-	0	-	0	-	0	0
	MCP	0	-	0	-	0	-	0	-	0	0
8b	LASSO	0	0	0	0	0	0	0	0	0	0
	SCAD	0	0	0	0	0	0	0	0	0	0
	MCP	0	0	0	0	0	0	0	0	0	0
9a	LASSO	3	0	0	0	0	0	0	0	0	0
	SCAD	3	0	0	0	0	0	0	0	0	0
	MCP	3	0	0	0	0	0	0	0	0	0
9b	LASSO	3	0	0	0	0	0	0	0	0	0
	SCAD	3	0	0	0	0	0	0	0	0	0
	MCP	3	0	0	0	0	0	0	0	0	0

Case	Method	Seq	AIC1	AIC2	BIC1	BIC2	L_q-A1	L_q-A2	L_q-B1	L_q-B2	CV
10a	LASSO	3	0	0	0	0	0	0	0	0	0
	SCAD	3	0	0	0	0	0	0	0	0	0
	MCP	3	0	0	0	0	0	0	0	0	0
10b	LASSO	3	0	0	0	0	0	0	0	0	0
	SCAD	3	0	0	0	0	0	0	0	0	0
	MCP	3	0	0	0	0	0	0	0	0	0
11a	LASSO	3	0	0	0	0	0	0	0	0	0
	SCAD	3	0	0	0	0	0	0	0	0	0
	MCP	3	0	0	0	0	0	0	0	0	0
11b	LASSO	3	0	0	0	0	0	0	0	0	0
	SCAD	3	0	0	0	0	0	0	0	0	0
	MCP	3	0	0	0	0	0	0	0	0	0
12a	LASSO	10	7	0	7	0	0	0	0	0	0
	SCAD	10	7	1	7	1	0	0	0	0	0
	MCP	11	7	1	7	2	0	0	0	0	0
12b	LASSO	3	0	0	0	0	0	0	0	0	0
	SCAD	3	0	0	0	0	0	0	0	0	0
	MCP	3	0	0	0	0	0	0	0	0	0
13a	LASSO	0	-	0	-	0	-	0	-	0	0
	SCAD	0	-	0	-	0	-	0	-	0	0
	MCP	0	-	0	-	0	-	0	-	0	0
13b	LASSO	0	0	0	0	0	0	0	0	0	0
	SCAD	0	0	0	0	0	0	0	0	0	0
	MCP	0	0	0	0	0	0	0	0	0	0
14a	LASSO	0	-	0	-	0	-	0	-	0	0
	SCAD	0	-	0	-	0	-	0	-	0	0
	MCP	0	-	0	-	0	-	0	-	0	0
14b	LASSO	0	0	0	0	0	0	0	0	0	0
	SCAD	0	0	0	0	0	0	0	0	0	0
	MCP	0	0	0	0	0	0	0	0	0	0
15a	LASSO	0	-	0	-	0	-	0	-	0	0
	SCAD	0	-	0	-	0	-	0	-	0	0
	MCP	0	-	0	-	0	-	0	-	0	0
15b	LASSO	0	0	0	0	0	0	0	0	0	0
	SCAD	0	0	0	0	0	0	0	0	0	0
	MCP	0	0	0	0	0	0	0	0	0	0
16a	LASSO	0	-	0	-	0	-	0	-	0	0
	SCAD	0	-	0	-	0	-	0	-	0	0
	MCP	0	-	0	-	0	-	0	-	0	0
16b	LASSO	0	0	0	0	0	0	0	0	0	0
	SCAD	0	0	0	0	0	0	0	0	0	0
	MCP	0	0	0	0	0	0	0	0	0	0
17a	LASSO	3	0	0	0	0	0	0	0	0	0
	SCAD	4	0	0	0	0	0	0	0	0	0
	MCP	4	0	0	0	0	0	0	0	0	0
17b	LASSO	5	0	1	0	1	0	0	0	0	0
	SCAD	5	0	1	0	1	0	0	0	0	0
	MCP	5	0	0	0	0	0	0	0	0	0
18a	LASSO	3	0	0	0	0	0	0	0	0	0
	SCAD	4	0	0	0	0	0	0	0	0	0
	MCP	4	0	0	0	0	0	0	0	0	0
18b	LASSO	5	0	1	0	1	0	0	0	0	0
	SCAD	5	0	1	0	1	0	0	0	0	0
	MCP	5	0	0	0	0	0	0	0	0	0
19a	LASSO	3	0	0	0	0	0	0	0	0	0
	SCAD	4	0	0	0	0	0	0	0	0	0
	MCP	4	0	0	0	0	0	0	0	0	0
19b	LASSO	5	0	1	0	1	0	0	0	0	0
	SCAD	5	0	1	0	1	0	0	0	0	0
	MCP	5	0	0	0	0	0	0	0	0	0

Case	Method	Seq	AIC1	AIC2	BIC1	BIC2	L_q-A1	L_q-A2	L_q-B1	L_q-B2	CV
20a	LASSO	9	4	0	4	0	0	0	0	0	0
	SCAD	9	4	0	4	0	0	0	0	0	0
	MCP	9	4	0	4	0	0	0	0	0	0
20b	LASSO	5	0	1	0	1	0	0	0	0	0
	SCAD	5	0	1	0	1	0	0	0	0	0
	MCP	5	0	0	0	0	0	0	0	0	0
21a	LASSO	0	-	0	-	0	-	0	-	0	0
	SCAD	0	-	0	-	0	-	0	-	0	0
	MCP	0	-	0	-	0	-	0	-	0	0
21b	LASSO	0	0	0	0	0	0	0	0	0	0
	SCAD	0	0	0	0	0	0	0	0	0	0
	MCP	0	0	0	0	0	0	0	0	0	0
22a	LASSO	0	-	0	-	0	-	0	-	0	0
	SCAD	0	-	0	-	0	-	0	-	0	0
	MCP	0	-	0	-	0	-	0	-	0	0
22b	LASSO	0	0	0	0	0	0	0	0	0	0
	SCAD	0	0	0	0	0	0	0	0	0	0
	MCP	0	0	0	0	0	0	0	0	0	0
23a	LASSO	0	-	0	-	0	-	0	-	0	0
	SCAD	0	-	0	-	0	-	0	-	0	0
	MCP	0	-	0	-	0	-	0	-	0	0
23b	LASSO	0	0	0	0	0	0	0	0	0	0
	SCAD	0	0	0	0	0	0	0	0	0	0
	MCP	0	0	0	0	0	0	0	0	0	0
24a	LASSO	0	-	0	-	0	-	0	-	0	0
	SCAD	0	-	0	-	0	-	0	-	0	0
	MCP	0	-	0	-	0	-	0	-	0	0
24b	LASSO	0	0	0	0	0	0	0	0	0	0
	SCAD	0	0	0	0	0	0	0	0	0	0
	MCP	0	0	0	0	0	0	0	0	0	0
25a	LASSO	17	0	0	0	0	0	0	0	0	0
	SCAD	17	0	0	0	0	0	0	0	0	0
	MCP	14	0	0	0	0	0	0	0	0	0
25b	LASSO	20	0	0	0	0	0	0	0	0	0
	SCAD	23	0	0	0	0	0	0	0	0	0
	MCP	23	0	0	0	0	0	0	0	0	0
26a	LASSO	17	0	0	0	0	0	0	0	0	0
	SCAD	17	0	0	0	0	0	0	0	0	0
	MCP	14	0	0	0	0	0	0	0	0	0
26b	LASSO	20	0	0	0	0	0	0	0	0	0
	SCAD	23	0	0	0	0	0	0	0	0	0
	MCP	23	0	0	0	0	0	0	0	0	0
27a	LASSO	17	0	0	0	0	0	0	0	0	0
	SCAD	17	0	0	0	0	0	0	0	0	0
	MCP	14	0	0	0	0	0	0	0	0	0
27b	LASSO	20	0	0	0	0	0	0	0	0	0
	SCAD	23	0	0	0	0	0	0	0	0	0
	MCP	23	0	0	0	0	0	0	0	0	0
28a	LASSO	23	3	0	4	0	0	0	0	0	0
	SCAD	21	3	0	4	0	0	0	0	0	1
	MCP	22	3	0	4	1	0	0	0	0	0
28b	LASSO	20	0	0	0	0	0	0	0	0	0
	SCAD	23	0	0	0	0	0	0	0	0	0
	MCP	23	0	0	0	0	0	0	0	0	0
29a	LASSO	0	-	0	-	0	-	0	-	0	0
	SCAD	0	-	0	-	0	-	0	-	0	0
	MCP	0	-	0	-	0	-	0	-	0	0
29b	LASSO	0	0	0	0	0	0	0	0	0	0
	SCAD	0	0	0	0	0	0	0	0	0	0
	MCP	0	0	0	0	0	0	0	0	0	0

Case	Method	Seq	AIC1	AIC2	BIC1	BIC2	L_q-A1	L_q-A2	L_q-B1	L_q-B2	CV
30a	LASSO	0	-	0	-	0	-	0	-	0	0
	SCAD	0	-	0	-	0	-	0	-	0	0
	MCP	0	-	0	-	0	-	0	-	0	0
30b	LASSO	0	0	0	0	0	0	0	0	0	0
	SCAD	0	0	0	0	0	0	0	0	0	0
	MCP	0	0	0	0	0	0	0	0	0	0
31a	LASSO	0	-	0	-	0	-	0	-	0	0
	SCAD	0	-	0	-	0	-	0	-	0	0
	MCP	0	-	0	-	0	-	0	-	0	0
31b	LASSO	0	0	0	0	0	0	0	0	0	0
	SCAD	0	0	0	0	0	0	0	0	0	0
	MCP	0	0	0	0	0	0	0	0	0	0
32a	LASSO	0	-	0	-	0	-	0	-	0	0
	SCAD	0	-	0	-	0	-	0	-	0	0
	MCP	0	-	0	-	0	-	0	-	0	0
32b	LASSO	0	0	0	0	0	0	0	0	0	0
	SCAD	0	0	0	0	0	0	0	0	0	0
	MCP	0	0	0	0	0	0	0	0	0	0

Table S31: The results on generalization for $q = 1$ (the normal error). Note that, when $q = 1$, L_q-criteria are equivalent to the original criteria, respectively.

Case	Method	AIC1	AIC2	BIC1	BIC2	L_q-A1	L_q-A2	L_q-B1	L_q-B2	CV
1a	LASSO	1.04	1.07	1.03	1.04	-	-	-	-	1.04
	SCAD	1.06	1.08	1.04	1.05	-	-	-	-	1.04
	MCP	1.07	1.09	1.05	1.05	-	-	-	-	1.05
1b	LASSO	1.00	1.01	1.00	1.00	-	-	-	-	1.00
	SCAD	1.00	1.01	1.00	1.00	-	-	-	-	1.00
	MCP	1.01	1.01	1.00	1.00	-	-	-	-	1.00
2a	LASSO	1.07	1.08	1.08	1.07	-	-	-	-	1.07
	SCAD	1.06	1.08	1.04	1.05	-	-	-	-	1.04
	MCP	1.07	1.09	1.05	1.05	-	-	-	-	1.04
2b	LASSO	1.01	1.01	1.01	1.01	-	-	-	-	1.01
	SCAD	1.01	1.01	1.00	1.00	-	-	-	-	1.00
	MCP	1.01	1.01	1.00	1.00	-	-	-	-	1.00
3a	LASSO	1.07	1.08	1.08	1.07	-	-	-	-	1.07
	SCAD	1.06	1.08	1.04	1.05	-	-	-	-	1.04
	MCP	1.07	1.09	1.05	1.05	-	-	-	-	1.04
3b	LASSO	1.01	1.01	1.01	1.01	-	-	-	-	1.01
	SCAD	1.01	1.01	1.00	1.00	-	-	-	-	1.00
	MCP	1.01	1.01	1.00	1.00	-	-	-	-	1.00
4a	LASSO	1.07	1.07	1.08	1.07	-	-	-	-	1.07
	SCAD	1.05	1.04	1.04	1.04	-	-	-	-	1.04
	MCP	1.06	1.06	1.05	1.05	-	-	-	-	1.04
4b	LASSO	1.01	1.01	1.01	1.01	-	-	-	-	1.01
	SCAD	1.00	1.00	1.00	1.00	-	-	-	-	1.00
	MCP	1.01	1.00	1.00	1.00	-	-	-	-	1.00
5a	LASSO	-	1.44	-	1.18	-	-	-	-	1.20
	SCAD	-	1.79	-	1.42	-	-	-	-	1.19
	MCP	-	1.76	-	1.49	-	-	-	-	1.20
5b	LASSO	1.03	1.05	1.02	1.02	-	-	-	-	1.02
	SCAD	1.03	1.07	1.02	1.02	-	-	-	-	1.02
	MCP	1.05	1.07	1.02	1.02	-	-	-	-	1.02
6a	LASSO	-	1.55	-	1.56	-	-	-	-	1.55
	SCAD	-	1.36	-	1.26	-	-	-	-	1.22
	MCP	-	1.50	-	1.43	-	-	-	-	1.22
6b	LASSO	1.05	1.07	1.10	1.06	-	-	-	-	1.05
	SCAD	1.04	1.07	1.02	1.03	-	-	-	-	1.02
	MCP	1.05	1.07	1.02	1.02	-	-	-	-	1.02
7a	LASSO	-	21.02	-	21.02	-	-	-	-	21.02
	SCAD	-	1.20	-	1.20	-	-	-	-	1.20
	MCP	-	1.20	-	1.20	-	-	-	-	1.20
7b	LASSO	1.05	1.07	1.10	1.06	-	-	-	-	1.05
	SCAD	1.04	1.07	1.02	1.02	-	-	-	-	1.02
	MCP	1.05	1.07	1.02	1.02	-	-	-	-	1.02
8a	LASSO	-	1984	-	1984	-	-	-	-	1984
	SCAD	-	1.20	-	1.20	-	-	-	-	1.20
	MCP	-	1.20	-	1.20	-	-	-	-	1.20
8b	LASSO	1.06	1.06	1.10	1.07	-	-	-	-	1.06
	SCAD	1.03	1.03	1.02	1.02	-	-	-	-	1.02
	MCP	1.04	1.04	1.02	1.02	-	-	-	-	1.02
9a	LASSO	1.05	1.08	1.05	1.06	-	-	-	-	1.06
	SCAD	1.07	1.09	1.05	1.07	-	-	-	-	1.06
	MCP	1.08	1.09	1.06	1.07	-	-	-	-	1.07
9b	LASSO	1.00	1.01	1.00	1.00	-	-	-	-	1.00
	SCAD	1.01	1.01	1.00	1.01	-	-	-	-	1.01
	MCP	1.01	1.01	1.00	1.01	-	-	-	-	1.01

Case	Method	AIC1	AIC2	BIC1	BIC2	L_q-A1	L_q-A2	L_q-B1	L_q-B2	CV
10a	LASSO	1.10	1.10	1.13	1.10	-	-	-	-	1.09
	SCAD	1.08	1.09	1.06	1.07	-	-	-	-	1.06
	MCP	1.08	1.09	1.07	1.07	-	-	-	-	1.06
10b	LASSO	1.01	1.01	1.01	1.01	-	-	-	-	1.01
	SCAD	1.01	1.01	1.00	1.01	-	-	-	-	1.01
	MCP	1.01	1.01	1.01	1.01	-	-	-	-	1.01
11a	LASSO	1.10	1.10	1.13	1.10	-	-	-	-	1.09
	SCAD	1.07	1.09	1.06	1.07	-	-	-	-	1.06
	MCP	1.08	1.09	1.07	1.07	-	-	-	-	1.06
11b	LASSO	1.01	1.01	1.01	1.01	-	-	-	-	1.01
	SCAD	1.01	1.01	1.00	1.01	-	-	-	-	1.01
	MCP	1.01	1.01	1.01	1.01	-	-	-	-	1.01
12a	LASSO	1.12	1.11	1.14	1.12	-	-	-	-	1.11
	SCAD	1.06	1.06	1.06	1.06	-	-	-	-	1.06
	MCP	1.07	1.07	1.06	1.06	-	-	-	-	1.06
12b	LASSO	1.01	1.01	1.01	1.01	-	-	-	-	1.01
	SCAD	1.01	1.01	1.00	1.00	-	-	-	-	1.00
	MCP	1.01	1.01	1.01	1.01	-	-	-	-	1.01
13a	LASSO	-	1.49	-	1.31	-	-	-	-	1.34
	SCAD	-	1.80	-	1.59	-	-	-	-	1.34
	MCP	-	1.78	-	1.59	-	-	-	-	1.37
13b	LASSO	1.04	1.06	1.04	1.04	-	-	-	-	1.04
	SCAD	1.05	1.08	1.04	1.04	-	-	-	-	1.04
	MCP	1.06	1.08	1.04	1.04	-	-	-	-	1.04
14a	LASSO	-	2.53	-	2.53	-	-	-	-	2.53
	SCAD	-	1.87	-	1.86	-	-	-	-	4.67
	MCP	-	1.74	-	1.73	-	-	-	-	3.51
14b	LASSO	1.09	1.08	1.22	1.09	-	-	-	-	1.08
	SCAD	1.05	1.08	1.04	1.05	-	-	-	-	1.04
	MCP	1.06	1.08	1.04	1.04	-	-	-	-	1.04
15a	LASSO	-	99.2	-	99.2	-	-	-	-	99.2
	SCAD	-	16.5	-	16.5	-	-	-	-	103.6
	MCP	-	7.88	-	7.88	-	-	-	-	30.3
15b	LASSO	1.09	1.08	1.22	1.09	-	-	-	-	1.08
	SCAD	1.05	1.08	1.04	1.04	-	-	-	-	1.04
	MCP	1.06	1.08	1.04	1.04	-	-	-	-	1.04
16a	LASSO	-	9762	-	9762	-	-	-	-	9762
	SCAD	-	1557	-	1557	-	-	-	-	10511
	MCP	-	636	-	636	-	-	-	-	3473
16b	LASSO	1.13	1.13	1.22	1.13	-	-	-	-	1.13
	SCAD	1.04	1.04	1.04	1.04	-	-	-	-	1.04
	MCP	1.05	1.05	1.04	1.04	-	-	-	-	1.14
17a	LASSO	1.06	1.08	1.06	1.07	-	-	-	-	1.07
	SCAD	1.08	1.10	1.07	1.08	-	-	-	-	1.08
	MCP	1.09	1.10	1.07	1.08	-	-	-	-	1.08
17b	LASSO	1.01	1.01	1.01	1.01	-	-	-	-	1.01
	SCAD	1.01	1.01	1.01	1.01	-	-	-	-	1.01
	MCP	1.01	1.01	1.01	1.01	-	-	-	-	1.01
18a	LASSO	1.13	1.11	1.18	1.11	-	-	-	-	1.11
	SCAD	1.09	1.10	1.08	1.08	-	-	-	-	1.08
	MCP	1.09	1.10	1.08	1.09	-	-	-	-	1.08
18b	LASSO	1.01	1.01	1.02	1.01	-	-	-	-	1.01
	SCAD	1.01	1.01	1.01	1.01	-	-	-	-	1.01
	MCP	1.01	1.01	1.01	1.01	-	-	-	-	1.01
19a	LASSO	1.13	1.10	1.18	1.11	-	-	-	-	1.11
	SCAD	1.09	1.10	1.08	1.08	-	-	-	-	1.08
	MCP	1.09	1.10	1.08	1.09	-	-	-	-	1.08
19b	LASSO	1.01	1.01	1.02	1.01	-	-	-	-	1.01
	SCAD	1.01	1.01	1.01	1.01	-	-	-	-	1.01
	MCP	1.01	1.01	1.01	1.01	-	-	-	-	1.01

Case	Method	AIC1	AIC2	BIC1	BIC2	L_q-A1	L_q-A2	L_q-B1	L_q-B2	CV
20a	LASSO	1.18	1.17	1.21	1.18	-	-	-	-	1.17
	SCAD	1.08	1.08	1.08	1.07	-	-	-	-	1.07
	MCP	1.08	1.08	1.08	1.08	-	-	-	-	1.08
20b	LASSO	1.01	1.01	1.02	1.01	-	-	-	-	1.01
	SCAD	1.01	1.01	1.01	1.01	-	-	-	-	1.01
	MCP	1.01	1.01	1.01	1.01	-	-	-	-	1.01
21a	LASSO	-	1.52	-	1.40	-	-	-	-	1.46
	SCAD	-	1.83	-	1.67	-	-	-	-	1.49
	MCP	-	1.81	-	1.65	-	-	-	-	1.53
21b	LASSO	1.05	1.07	1.05	1.06	-	-	-	-	1.05
	SCAD	1.06	1.09	1.05	1.06	-	-	-	-	1.06
	MCP	1.07	1.09	1.06	1.06	-	-	-	-	1.06
22a	LASSO	-	3.78	-	3.78	-	-	-	-	3.97
	SCAD	-	3.05	-	3.06	-	-	-	-	19.9
	MCP	-	2.68	-	2.68	-	-	-	-	19.2
22b	LASSO	1.13	1.10	1.38	1.10	-	-	-	-	1.09
	SCAD	1.07	1.09	1.06	1.06	-	-	-	-	1.06
	MCP	1.08	1.09	1.06	1.06	-	-	-	-	1.06
23a	LASSO	-	225.5	-	225.5	-	-	-	-	232.6
	SCAD	-	127.7	-	127.7	-	-	-	-	1633
	MCP	-	85.16	-	85.16	-	-	-	-	1424
23b	LASSO	1.12	1.10	1.40	1.10	-	-	-	-	1.09
	SCAD	1.07	1.09	1.06	1.06	-	-	-	-	1.06
	MCP	1.08	1.09	1.06	1.06	-	-	-	-	1.06
24a	LASSO	-	22405	-	22405	-	-	-	-	22991
	SCAD	-	12795	-	12795	-	-	-	-	164365
	MCP	-	8537	-	8537	-	-	-	-	141781
24b	LASSO	1.22	1.22	1.40	1.22	-	-	-	-	1.22
	SCAD	1.06	1.06	1.06	1.06	-	-	-	-	1.06
	MCP	1.06	1.06	1.06	1.06	-	-	-	-	1.06
25a	LASSO	1.07	1.09	1.07	1.08	-	-	-	-	1.08
	SCAD	1.09	1.10	1.08	1.10	-	-	-	-	1.09
	MCP	1.10	1.10	1.09	1.09	-	-	-	-	1.10
25b	LASSO	1.01	1.01	1.01	1.01	-	-	-	-	1.01
	SCAD	1.01	1.01	1.01	1.01	-	-	-	-	1.01
	MCP	1.01	1.01	1.01	1.01	-	-	-	-	1.01
26a	LASSO	1.14	1.11	1.20	1.11	-	-	-	-	1.11
	SCAD	1.10	1.10	1.10	1.10	-	-	-	-	1.10
	MCP	1.10	1.11	1.10	1.10	-	-	-	-	1.10
26b	LASSO	1.01	1.01	1.02	1.01	-	-	-	-	1.01
	SCAD	1.01	1.01	1.01	1.01	-	-	-	-	1.01
	MCP	1.01	1.01	1.01	1.01	-	-	-	-	1.01
27a	LASSO	1.32	1.11	1.48	1.11	-	-	-	-	1.11
	SCAD	1.10	1.10	1.10	1.10	-	-	-	-	1.10
	MCP	1.10	1.11	1.10	1.10	-	-	-	-	1.10
27b	LASSO	1.01	1.01	1.02	1.01	-	-	-	-	1.01
	SCAD	1.01	1.01	1.01	1.01	-	-	-	-	1.01
	MCP	1.01	1.01	1.01	1.01	-	-	-	-	1.01
28a	LASSO	1.43	1.23	1.56	1.24	-	-	-	-	1.24
	SCAD	1.09	1.09	1.09	1.09	-	-	-	-	1.09
	MCP	1.09	1.09	1.09	1.09	-	-	-	-	1.09
28b	LASSO	1.02	1.02	1.02	1.02	-	-	-	-	1.02
	SCAD	1.01	1.01	1.01	1.01	-	-	-	-	1.01
	MCP	1.01	1.01	1.01	1.01	-	-	-	-	1.01
29a	LASSO	-	1.55	-	1.47	-	-	-	-	1.57
	SCAD	-	1.84	-	1.72	-	-	-	-	1.64
	MCP	-	1.83	-	1.71	-	-	-	-	1.70
29b	LASSO	1.06	1.08	1.07	1.08	-	-	-	-	1.07
	SCAD	1.07	1.10	1.07	1.08	-	-	-	-	1.07
	MCP	1.08	1.10	1.07	1.08	-	-	-	-	1.08

Case	Method	AIC1	AIC2	BIC1	BIC2	L_q-A1	L_q-A2	L_q-B1	L_q-B2	CV
30a	LASSO	-	4.76	-	4.76	-	-	-	-	5.63
	SCAD	-	3.54	-	3.55	-	-	-	-	32.16
	MCP	-	3.02	-	3.02	-	-	-	-	34.25
30b	LASSO	1.17	1.10	1.51	1.10	-	-	-	-	1.10
	SCAD	1.09	1.09	1.08	1.08	-	-	-	-	1.08
	MCP	1.09	1.09	1.08	1.08	-	-	-	-	1.08
31a	LASSO	-	321.6	-	321.6	-	-	-	-	394.4
	SCAD	-	174.2	-	174.2	-	-	-	-	2940
	MCP	-	119.1	-	119.1	-	-	-	-	2833
31b	LASSO	1.16	1.10	1.72	1.10	-	-	-	-	1.10
	SCAD	1.09	1.09	1.08	1.08	-	-	-	-	1.08
	MCP	1.09	1.09	1.08	1.08	-	-	-	-	1.08
32a	LASSO	-	32013	-	32013	-	-	-	-	38963
	SCAD	-	17287	-	17287	-	-	-	-	295232
	MCP	-	11803	-	11803	-	-	-	-	283219
32b	LASSO	1.36	1.33	2.12	1.33	-	-	-	-	1.33
	SCAD	19.38	1.08	1.08	1.08	-	-	-	-	1.08
	MCP	1.08	1.08	1.08	1.08	-	-	-	-	1.08

Table S32: The results on generalization for $q = 13/11$ (the t-error with $\nu = 10$).

Case	Method	AIC1	AIC2	BIC1	BIC2	L_q-A1	L_q-A2	L_q-B1	L_q-B2	CV
1a	LASSO	1.28	1.29	1.27	1.27	1.27	1.27	1.27	1.27	1.29
	SCAD	1.28	1.30	1.27	1.28	1.27	1.27	1.27	1.27	1.29
	MCP	1.30	1.31	1.28	1.28	1.27	1.27	1.27	1.27	1.29
1b	LASSO	1.25	1.26	1.25	1.25	1.25	1.25	1.25	1.25	1.25
	SCAD	1.25	1.26	1.25	1.25	1.25	1.25	1.25	1.25	1.25
	MCP	1.26	1.26	1.25	1.25	1.25	1.25	1.25	1.25	1.26
2a	LASSO	1.33	1.33	1.36	1.34	3.24	3.24	3.24	3.24	1.33
	SCAD	1.30	1.31	1.29	1.29	3.24	3.24	3.24	3.24	1.29
	MCP	1.31	1.31	1.29	1.29	3.24	3.24	3.24	3.24	1.30
2b	LASSO	1.26	1.26	1.26	1.26	1.45	1.45	1.45	1.45	1.26
	SCAD	1.26	1.26	1.25	1.25	1.45	1.45	1.45	1.45	1.26
	MCP	1.26	1.26	1.25	1.25	1.45	1.45	1.45	1.45	1.26
3a	LASSO	1.33	1.33	1.36	1.34	199.82	199.82	199.82	199.82	1.33
	SCAD	1.30	1.31	1.28	1.28	199.82	199.82	199.82	199.82	1.29
	MCP	1.31	1.31	1.29	1.29	199.82	199.82	199.82	199.82	1.29
3b	LASSO	1.26	1.26	1.26	1.26	21.27	21.27	21.27	21.27	1.26
	SCAD	1.26	1.26	1.25	1.25	21.27	21.27	21.27	21.27	1.26
	MCP	1.26	1.26	1.25	1.25	21.27	21.27	21.27	21.27	1.26
4a	LASSO	1.33	1.32	1.36	1.33	19861.33	19861.33	19861.33	19861.33	1.32
	SCAD	1.29	1.29	1.28	1.28	19861.33	19861.33	19861.33	19861.33	1.29
	MCP	1.30	1.31	1.29	1.29	19861.33	19861.33	19861.33	19861.33	1.29
4b	LASSO	1.26	1.26	1.26	1.26	2002.16	2002.16	2002.16	2002.16	1.26
	SCAD	1.26	1.26	1.25	1.25	2002.16	2002.16	2002.16	2002.16	1.25
	MCP	1.26	1.26	1.25	1.25	2002.16	2002.16	2002.16	2002.16	1.26
5a	LASSO	-	1.49	-	1.46	-	1.47	-	1.47	1.48
	SCAD	-	2.03	-	1.47	-	1.47	-	1.47	1.47
	MCP	-	2.03	-	1.49	-	1.47	-	1.47	1.48
5b	LASSO	1.27	1.27	1.27	1.27	1.27	1.27	1.27	1.27	1.27
	SCAD	1.27	1.28	1.27	1.27	1.27	1.27	1.27	1.27	1.27
	MCP	1.28	1.29	1.27	1.27	1.27	1.27	1.27	1.27	1.27
6a	LASSO	-	1.94	-	2.04	-	21.35	-	21.35	1.93
	SCAD	-	1.70	-	1.57	-	21.35	-	21.35	1.55
	MCP	-	1.92	-	1.57	-	21.35	-	21.35	1.55
6b	LASSO	1.34	1.32	1.46	1.35	3.26	3.26	3.26	3.26	1.32
	SCAD	1.28	1.28	1.30	1.29	3.26	3.26	3.26	3.26	1.28
	MCP	1.29	1.29	1.28	1.28	3.26	3.26	3.26	3.26	1.28
7a	LASSO	-	21.20	-	21.20	-	2009.84	-	2009.84	21.20
	SCAD	-	1.52	-	1.52	-	2009.84	-	2009.84	1.52
	MCP	-	1.52	-	1.52	-	2009.84	-	2009.84	1.52
7b	LASSO	1.34	1.32	1.46	1.35	201.58	201.58	201.58	201.58	1.32
	SCAD	1.28	1.28	1.28	1.28	201.58	201.58	201.58	201.58	1.28
	MCP	1.29	1.29	1.28	1.28	201.58	201.58	201.58	201.58	1.28
8a	LASSO	-	1965	-	1965	-	200862	-	200862	1965
	SCAD	-	1.52	-	1.52	-	200862	-	200862	1.52
	MCP	-	1.52	-	1.52	-	200862	-	200862	1.52
8b	LASSO	1.34	1.32	1.46	1.35	20032	20032	20032	20032	1.32
	SCAD	1.28	1.28	1.28	1.28	20032	20032	20032	20032	1.28
	MCP	1.29	1.29	1.28	1.28	20032	20032	20032	20032	1.28
9a	LASSO	1.29	1.31	1.29	1.29	1.29	1.29	1.29	1.29	1.30
	SCAD	1.30	1.32	1.29	1.30	1.29	1.29	1.29	1.29	1.31
	MCP	1.31	1.33	1.30	1.30	1.29	1.29	1.29	1.29	1.31
9b	LASSO	1.26	1.26	1.26	1.26	1.26	1.26	1.26	1.26	1.26
	SCAD	1.26	1.26	1.26	1.26	1.26	1.26	1.26	1.26	1.26
	MCP	1.26	1.26	1.26	1.26	1.26	1.26	1.26	1.26	1.26

Case	Method	AIC1	AIC2	BIC1	BIC2	L_q-A1	L_q-A2	L_q-B1	L_q-B2	CV
10a	LASSO	1.39	1.36	1.45	1.37	5.25	5.25	5.25	5.25	1.36
	SCAD	1.32	1.33	1.31	1.31	5.25	5.25	5.25	5.25	1.32
	MCP	1.33	1.34	1.31	1.31	5.25	5.25	5.25	5.25	1.32
10b	LASSO	1.26	1.26	1.27	1.27	1.65	1.65	1.65	1.65	1.26
	SCAD	1.26	1.26	1.26	1.26	1.65	1.65	1.65	1.65	1.26
	MCP	1.26	1.26	1.26	1.26	1.65	1.65	1.65	1.65	1.26
11a	LASSO	1.40	1.36	1.45	1.37	402.11	402.11	402.11	402.11	1.36
	SCAD	1.32	1.33	1.31	1.31	402.11	402.11	402.11	402.11	1.32
	MCP	1.33	1.33	1.31	1.31	402.11	402.11	402.11	402.11	1.32
11b	LASSO	1.26	1.26	1.27	1.26	41.30	41.30	41.30	41.30	1.26
	SCAD	1.26	1.26	1.26	1.26	41.30	41.30	41.30	41.30	1.26
	MCP	1.26	1.26	1.26	1.26	41.30	41.30	41.30	41.30	1.26
12a	LASSO	1.40	1.37	1.45	1.38	40100	40100	40100	40100	1.37
	SCAD	1.31	1.31	1.31	1.31	40100	40100	40100	40100	1.31
	MCP	1.32	1.32	1.31	1.31	40100	40100	40100	40100	1.31
12b	LASSO	1.26	1.26	1.27	1.27	4006	4006	4006	4006	1.26
	SCAD	1.26	1.26	1.26	1.26	4006	4006	4006	4006	1.26
	MCP	1.26	1.26	1.26	1.26	4006	4006	4006	4006	1.26
13a	LASSO	-	1.63	-	1.65	-	1.66	-	1.66	1.63
	SCAD	-	2.12	-	1.65	-	1.66	-	1.66	1.63
	MCP	-	2.09	-	1.68	-	1.66	-	1.66	1.65
13b	LASSO	1.29	1.29	1.29	1.29	1.29	1.29	1.29	1.29	1.29
	SCAD	1.29	1.30	1.29	1.29	1.29	1.29	1.29	1.29	1.29
	MCP	1.30	1.31	1.29	1.29	1.29	1.29	1.29	1.29	1.29
14a	LASSO	-	2.95	-	2.97	-	41.18	-	41.18	2.96
	SCAD	-	2.39	-	2.41	-	41.18	-	41.18	6.03
	MCP	-	2.56	-	2.24	-	41.18	-	41.18	4.84
14b	LASSO	1.42	1.35	1.75	1.39	5.25	5.25	5.25	5.25	1.35
	SCAD	1.31	1.31	1.37	1.32	5.25	5.25	5.25	5.25	1.30
	MCP	1.31	1.31	1.31	1.31	5.25	5.25	5.25	5.25	1.30
15a	LASSO	-	100.23	-	100.23	-	3992.99	-	3992.99	100.23
	SCAD	-	17.21	-	17.23	-	3992.99	-	3992.99	112.86
	MCP	-	7.86	-	7.87	-	3992.99	-	3992.99	45.44
15b	LASSO	1.42	1.35	1.77	1.39	401.25	401.25	401.25	401.25	1.35
	SCAD	1.31	1.31	1.30	1.30	401.25	401.25	401.25	401.25	1.30
	MCP	1.31	1.31	1.30	1.30	401.25	401.25	401.25	401.25	1.30
16a	LASSO	-	9830	-	9830	-	399173	-	399173	9830
	SCAD	-	1540	-	1540	-	399173	-	399173	11771
	MCP	-	728	-	729	-	399173	-	399173	3741
16b	LASSO	1.43	1.39	1.77	1.40	4007	4007	4007	4007	1.39
	SCAD	1.31	1.30	1.30	1.30	4007	4007	4007	4007	1.30
	MCP	1.31	1.31	1.30	1.30	4007	4007	4007	4007	1.30
17a	LASSO	1.31	1.32	1.31	1.31	1.31	1.31	1.31	1.31	1.32
	SCAD	1.31	1.34	1.31	1.32	1.31	1.31	1.31	1.31	1.33
	MCP	1.33	1.34	1.32	1.32	1.31	1.31	1.31	1.31	1.33
17b	LASSO	1.26	1.26	1.26	1.26	1.26	1.26	1.26	1.26	1.26
	SCAD	1.26	1.26	1.26	1.26	1.26	1.26	1.26	1.26	1.26
	MCP	1.26	1.26	1.26	1.26	1.26	1.26	1.26	1.26	1.26
18a	LASSO	1.47	1.37	1.52	1.38	7.22	7.22	7.22	7.22	1.37
	SCAD	1.34	1.35	1.34	1.34	7.22	7.22	7.22	7.22	1.35
	MCP	1.35	1.35	1.34	1.34	7.22	7.22	7.22	7.22	1.35
18b	LASSO	1.27	1.26	1.28	1.27	1.85	1.85	1.85	1.85	1.26
	SCAD	1.26	1.26	1.26	1.26	1.85	1.85	1.85	1.85	1.26
	MCP	1.26	1.26	1.26	1.26	1.85	1.85	1.85	1.85	1.26
19a	LASSO	1.45	1.37	1.53	1.38	600.25	600.25	600.25	600.25	1.37
	SCAD	1.34	1.35	1.33	1.34	600.25	600.25	600.25	600.25	1.34
	MCP	1.35	1.35	1.34	1.34	600.25	600.25	600.25	600.25	1.34
19b	LASSO	1.27	1.26	1.28	1.27	61.45	61.45	61.45	61.45	1.26
	SCAD	1.26	1.26	1.26	1.26	61.45	61.45	61.45	61.45	1.26
	MCP	1.26	1.26	1.26	1.26	61.45	61.45	61.45	61.45	1.26

Case	Method	AIC1	AIC2	BIC1	BIC2	L_q-A1	L_q-A2	L_q-B1	L_q-B2	CV
20a	LASSO	1.47	1.43	1.54	1.44	59924	59924	59924	59924	1.43
	SCAD	1.33	1.33	1.33	1.33	59924	59924	59924	59924	1.33
	MCP	1.34	1.34	1.33	1.33	59924	59924	59924	59924	1.34
20b	LASSO	1.27	1.27	1.28	1.27	6022	6022	6022	6022	1.27
	SCAD	1.26	1.26	1.26	1.26	6022	6022	6022	6022	1.26
	MCP	1.26	1.26	1.26	1.26	6022	6022	6022	6022	1.26
21a	LASSO	-	1.73	-	1.83	-	1.86	-	1.86	1.76
	SCAD	-	2.17	-	1.84	-	1.86	-	1.86	1.78
	MCP	-	2.14	-	1.84	-	1.86	-	1.86	1.81
21b	LASSO	1.30	1.31	1.31	1.31	1.31	1.31	1.31	1.31	1.31
	SCAD	1.30	1.32	1.31	1.31	1.31	1.31	1.31	1.31	1.31
	MCP	1.31	1.33	1.31	1.31	1.31	1.31	1.31	1.31	1.31
22a	LASSO	-	4.17	-	4.18	-	61.05	-	61.05	4.43
	SCAD	-	3.51	-	3.56	-	61.05	-	61.05	20.07
	MCP	-	3.12	-	3.16	-	61.05	-	61.05	19.42
22b	LASSO	1.52	1.37	1.99	1.40	7.26	7.26	7.26	7.26	1.36
	SCAD	1.33	1.33	1.39	1.34	7.26	7.26	7.26	7.26	1.33
	MCP	1.33	1.34	1.33	1.33	7.26	7.26	7.26	7.26	1.33
23a	LASSO	-	224.32	-	224.32	-	5985	-	5985	235.42
	SCAD	-	127.43	-	127.59	-	5985	-	5985	1609.98
	MCP	-	85.82	-	85.90	-	5985	-	5985	1351.60
23b	LASSO	1.54	1.37	2.21	1.40	601..62	601..62	601..62	601..62	1.36
	SCAD	1.33	1.33	1.33	1.33	601..62	601..62	601..62	601..62	1.33
	MCP	1.33	1.34	1.33	1.33	601..62	601..62	601..62	601..62	1.33
24a	LASSO	-	22282	-	22288	-	598401	-	598401	23403
	SCAD	-	12921	-	12974	-	598401	-	598401	160901
	MCP	-	8716	-	8724	-	598401	-	598401	140537
24b	LASSO	1.57	1.49	2.22	1.49	60034	60034	60034	60034	1.49
	SCAD	1.33	1.33	1.33	1.33	60034	60034	60034	60034	1.33
	MCP	1.33	1.33	1.33	1.33	60034	60034	60034	60034	1.33
25a	LASSO	1.32	1.34	1.33	1.33	1.33	1.33	1.33	1.33	1.33
	SCAD	1.33	1.35	1.33	1.34	1.33	1.33	1.33	1.33	1.35
	MCP	1.34	1.35	1.33	1.34	1.33	1.33	1.33	1.33	1.35
25b	LASSO	1.26	1.26	1.26	1.26	1.26	1.26	1.26	1.26	1.26
	SCAD	1.26	1.26	1.26	1.26	1.26	1.26	1.26	1.26	1.26
	MCP	1.26	1.26	1.26	1.26	1.26	1.26	1.26	1.26	1.26
26a	LASSO	1.49	1.38	1.55	1.39	9.28	9.28	9.28	9.28	1.38
	SCAD	1.36	1.37	1.36	1.36	9.28	9.28	9.28	9.28	1.37
	MCP	1.37	1.37	1.36	1.36	9.28	9.28	9.28	9.28	1.37
26b	LASSO	1.27	1.26	1.28	1.27	2.06	2.06	2.06	2.06	1.26
	SCAD	1.26	1.26	1.26	1.26	2.06	2.06	2.06	2.06	1.26
	MCP	1.26	1.26	1.26	1.26	2.06	2.06	2.06	2.06	1.26
27a	LASSO	1.49	1.38	1.55	1.38	804.48	804.48	804.48	804.48	1.38
	SCAD	1.36	1.37	1.36	1.36	804.48	804.48	804.48	804.48	1.37
	MCP	1.37	1.37	1.36	1.36	804.48	804.48	804.48	804.48	1.36
27b	LASSO	1.27	1.26	1.28	1.27	81.34	81.34	81.34	81.34	1.26
	SCAD	1.26	1.26	1.26	1.26	81.34	81.34	81.34	81.34	1.26
	MCP	1.26	1.26	1.26	1.26	81.34	81.34	81.34	81.34	1.26
28a	LASSO	1.56	1.51	1.61	1.51	80327	80327	80327	80327	1.51
	SCAD	1.36	1.36	1.36	1.36	80327	80327	80327	80327	1.36
	MCP	1.36	1.36	1.36	1.36	80327	80327	80327	80327	1.36
28b	LASSO	1.27	1.27	1.28	1.27	8006	8006	8006	8006	1.27
	SCAD	1.26	1.26	1.26	1.26	8006	8006	8006	8006	1.26
	MCP	1.26	1.26	1.26	1.26	8006	8006	8006	8006	1.26
29a	LASSO	-	1.81	-	2.01	-	2.06	-	2.06	1.87
	SCAD	-	2.20	-	2.02	-	2.06	-	2.06	1.92
	MCP	-	2.18	-	1.97	-	2.06	-	2.06	1.97
29b	LASSO	1.32	1.32	1.33	1.33	1.33	1.33	1.33	1.33	1.32
	SCAD	1.32	1.34	1.33	1.33	1.33	1.33	1.33	1.33	1.32
	MCP	1.33	1.34	1.33	1.33	1.33	1.33	1.33	1.33	1.33

Case	Method	AIC1	AIC2	BIC1	BIC2	L_q-A1	L_q-A2	L_q-B1	L_q-B2	CV
30a	LASSO	-	5.19	-	5.20	-	81.20	-	81.20	5.98
	SCAD	-	4.03	-	4.08	-	81.20	-	81.20	32.41
	MCP	-	3.51	-	3.54	-	81.20	-	81.20	33.11
30b	LASSO	1.63	1.37	2.09	1.39	9.26	9.26	9.26	9.26	1.37
	SCAD	1.35	1.36	1.40	1.36	9.26	9.26	9.26	9.26	1.35
	MCP	1.36	1.36	1.35	1.35	9.26	9.26	9.26	9.26	1.35
31a	LASSO	-	325.87	-	325.87	-	8001.53	-	8001.53	386.17
	SCAD	-	177.62	-	178.04	-	8001.53	-	8001.53	2957.55
	MCP	-	122.05	-	122.20	-	8001.53	-	8001.53	3004.89
31b	LASSO	1.70	1.37	3.07	1.39	801.68	801.68	801.68	801.68	1.37
	SCAD	1.35	1.36	1.35	1.35	801.68	801.68	801.68	801.68	1.35
	MCP	1.36	1.36	1.35	1.35	801.68	801.68	801.68	801.68	1.35
32a	LASSO	-	32492	-	32497	-	800092	-	800092	38123
	SCAD	-	18310	-	18382	-	800092	-	800092	289151
	MCP	-	12433	-	12456	-	800092	-	800092	299076
32b	LASSO	1.78	1.60	3.12	1.60	80040	80040	80040	80040	1.60
	SCAD	1.35	1.35	1.35	1.35	80040	80040	80040	80040	1.35
	MCP	1.35	1.35	1.35	1.35	80040	80040	80040	80040	1.35

Table S33: The results on generalization for $q = 3/2$ (the t-error with $\nu = 3$).

Case	Method	AIC1	AIC2	BIC1	BIC2	L_q-A1	L_q-A2	L_q-B1	L_q-B2	CV
1a	LASSO	2.88	2.89	2.87	2.87	2.90	2.91	2.88	2.89	2.89
	SCAD	2.89	2.90	2.87	2.87	2.91	2.93	2.88	2.90	2.89
	MCP	2.90	2.90	2.88	2.88	2.93	2.94	2.89	2.90	2.90
1b	LASSO	2.92	2.92	2.92	2.92	2.92	2.92	2.92	2.92	2.92
	SCAD	2.92	2.92	2.92	2.92	2.92	2.92	2.92	2.92	2.92
	MCP	2.92	2.92	2.92	2.92	2.92	2.92	2.92	2.92	2.92
2a	LASSO	3.08	3.00	3.11	3.02	3.07	3.05	3.12	3.09	3.02
	SCAD	3.01	2.95	3.02	2.94	2.99	2.99	3.03	3.00	2.96
	MCP	2.97	2.95	2.96	2.93	2.97	2.98	2.97	2.97	2.97
2b	LASSO	2.94	2.93	2.95	2.94	2.94	2.94	2.95	2.95	2.94
	SCAD	2.94	2.93	2.94	2.93	2.93	2.93	2.94	2.94	2.93
	MCP	2.93	2.93	2.93	2.93	2.93	2.93	2.93	2.93	2.93
3a	LASSO	3.10	3.01	3.13	3.02	3.07	3.05	3.12	3.08	3.02
	SCAD	2.92	2.93	2.91	2.91	2.94	2.96	2.92	2.93	2.93
	MCP	2.93	2.94	2.91	2.91	2.96	2.97	2.93	2.93	2.94
3b	LASSO	2.94	2.93	2.95	2.94	2.94	2.94	2.95	2.94	2.94
	SCAD	2.93	2.93	2.92	2.92	2.93	2.93	2.93	2.93	2.93
	MCP	2.93	2.93	2.93	2.92	2.93	2.93	2.93	2.93	2.93
4a	LASSO	3.10	3.00	3.13	3.02	3.07	3.05	3.12	3.08	3.01
	SCAD	2.92	2.93	2.91	2.91	2.94	2.96	2.92	2.93	2.92
	MCP	2.93	2.94	2.91	2.91	2.96	2.97	2.93	2.93	2.93
4b	LASSO	2.94	2.93	2.95	2.94	2.94	2.94	2.95	2.94	2.94
	SCAD	2.93	2.93	2.92	2.92	2.93	2.93	2.93	2.93	2.93
	MCP	2.93	2.93	2.93	2.93	2.93	2.93	2.93	2.93	2.93
5a	LASSO	-	3.49	-	3.29	-	3.55	-	10.56	41.90
	SCAD	-	5.31	-	3.29	-	3.85	-	3.61	3.31
	MCP	-	5.28	-	3.32	-	3.98	-	3.61	3.32
5b	LASSO	2.92	2.92	2.92	2.92	2.92	2.93	2.92	2.92	2.92
	SCAD	2.92	2.92	2.92	2.92	2.93	2.93	2.92	2.92	2.92
	MCP	2.92	2.93	2.92	2.92	2.93	2.93	2.92	2.92	2.92
6a	LASSO	-	4.74	-	4.73	-	4.96	-	4.82	4.65
	SCAD	-	5.17	-	4.64	-	4.68	-	4.80	4.47
	MCP	-	5.50	-	4.68	-	4.62	-	4.65	4.52
6b	LASSO	3.31	3.05	3.42	3.10	3.21	3.09	3.48	3.20	3.05
	SCAD	3.21	2.99	3.34	3.02	3.08	3.03	3.42	3.10	2.99
	MCP	3.06	2.99	3.11	3.00	3.00	3.01	3.17	3.04	2.99
7a	LASSO	-	23.55	-	23.55	-	23.55	-	23.55	23.55
	SCAD	-	3.73	-	3.72	-	3.75	-	3.75	3.73
	MCP	-	3.74	-	3.73	-	3.77	-	3.77	3.72
7b	LASSO	3.37	3.05	3.50	3.10	3.21	3.09	3.49	3.21	3.05
	SCAD	2.96	2.96	2.96	2.96	2.97	2.97	2.96	2.96	2.96
	MCP	2.96	2.96	2.96	2.96	2.97	2.97	2.96	2.96	2.96
8a	LASSO	-	1973	-	1973	-	1973	-	1973	1973
	SCAD	-	3.72	-	3.72	-	3.72	-	3.72	3.72
	MCP	-	3.72	-	3.72	-	3.72	-	3.72	3.72
8b	LASSO	3.37	3.05	3.50	3.10	3.21	3.09	3.49	3.21	3.05
	SCAD	2.96	2.96	2.96	2.96	2.97	2.97	2.96	2.96	2.96
	MCP	2.96	2.96	2.96	2.96	2.97	2.97	2.96	2.96	2.96
9a	LASSO	2.90	2.91	2.89	2.89	2.92	2.93	2.90	2.91	2.91
	SCAD	2.90	2.91	2.89	2.89	2.93	2.95	2.90	2.92	2.91
	MCP	2.92	2.93	2.89	2.90	2.95	2.96	2.91	2.92	2.92
9b	LASSO	2.92	2.92	2.92	2.92	2.92	2.92	2.92	2.92	2.93
	SCAD	2.92	2.92	2.92	2.92	2.92	2.92	2.92	2.92	2.93
	MCP	2.92	2.93	2.92	2.92	2.93	2.93	2.92	2.92	2.93

Case	Method	AIC1	AIC2	BIC1	BIC2	L_q-A1	L_q-A2	L_q-B1	L_q-B2	CV
10a	LASSO	3.26	3.07	3.34	3.09	3.26	3.12	3.36	3.20	3.09
	SCAD	3.12	3.02	3.16	3.01	3.08	3.05	3.18	3.06	3.04
	MCP	3.05	3.02	3.04	3.01	3.03	3.03	3.05	3.03	3.04
10b	LASSO	2.96	2.94	2.97	2.95	2.96	2.95	2.97	2.96	2.94
	SCAD	2.95	2.93	2.95	2.94	2.94	2.94	2.96	2.94	2.94
	MCP	2.94	2.93	2.94	2.93	2.94	2.94	2.94	2.94	2.94
11a	LASSO	3.33	3.08	3.41	3.10	3.27	3.13	3.38	3.20	3.09
	SCAD	2.98	2.99	2.96	2.96	3.00	3.01	2.97	2.98	2.99
	MCP	2.99	2.99	2.97	2.97	3.01	3.02	2.98	2.98	2.99
11b	LASSO	2.96	2.94	2.97	2.95	2.96	2.95	2.97	2.96	2.94
	SCAD	2.93	2.93	2.93	2.93	2.93	2.93	2.93	2.93	2.93
	MCP	2.93	2.93	2.93	2.93	2.93	2.93	2.93	2.93	2.93
12a	LASSO	3.33	3.08	3.41	3.11	3.27	3.12	3.38	3.20	3.08
	SCAD	2.98	2.98	2.96	2.96	3.00	3.01	2.97	2.98	2.97
	MCP	2.99	2.99	2.97	2.97	3.01	3.02	2.98	2.98	2.98
12b	LASSO	2.96	2.94	2.97	2.95	2.96	2.94	2.97	2.96	2.94
	SCAD	2.93	2.93	2.93	2.93	2.93	2.93	2.93	2.93	2.93
	MCP	2.93	2.93	2.93	2.93	2.93	2.93	2.93	2.93	2.93
13a	LASSO	-	3.80	-	3.50	-	3.75	-	3.65	3.53
	SCAD	-	5.46	-	3.50	-	4.08	-	3.82	3.50
	MCP	-	5.40	-	3.56	-	4.18	-	3.84	3.51
13b	LASSO	2.94	2.94	2.94	2.94	2.94	2.95	2.94	2.94	2.94
	SCAD	2.94	2.94	2.94	2.94	2.94	2.95	2.94	2.94	2.94
	MCP	2.94	2.95	2.94	2.94	2.95	2.95	2.94	2.94	2.94
14a	LASSO	-	5.82	-	5.83	-	5.93	-	6.97	5.87
	SCAD	-	6.04	-	6.08	-	6.20	-	6.99	11.92
	MCP	-	5.93	-	5.96	-	6.18	-	7.36	11.49
14b	LASSO	3.63	3.12	3.83	3.17	3.56	3.16	4.04	3.33	3.12
	SCAD	3.42	3.06	3.65	3.08	3.26	3.09	3.90	3.19	3.05
	MCP	3.18	3.05	3.27	3.07	3.09	3.08	3.43	3.14	3.05
15a	LASSO	-	103.52	-	103.53	-	103.52	-	103.52	103.52
	SCAD	-	51.08	-	49.24	-	51.56	-	51.55	46.11
	MCP	-	11.28	-	11.32	-	11.23	-	11.23	49.64
15b	LASSO	3.90	3.12	4.24	3.18	3.60	3.16	4.22	3.33	3.12
	SCAD	3.02	3.02	3.02	3.02	3.02	3.02	3.02	3.02	3.02
	MCP	3.02	3.03	3.02	3.02	3.03	3.03	3.02	3.02	3.02
16a	LASSO	-	9900	-	9901	-	9900	-	9900	9900
	SCAD	-	1621	-	1637	-	1523	-	1523	10216
	MCP	-	705	-	714	-	677	-	677	4249
16b	LASSO	3.90	3.14	4.24	3.18	3.60	3.16	4.23	3.33	3.14
	SCAD	3.02	3.02	3.02	3.02	3.02	3.03	3.02	3.02	3.02
	MCP	3.02	3.02	3.02	3.02	3.03	3.03	3.02	3.02	3.02
17a	LASSO	2.91	2.92	2.91	2.91	2.93	2.59	2.92	2.93	2.92
	SCAD	2.92	2.93	2.91	2.91	2.94	2.97	2.92	2.94	2.93
	MCP	2.93	2.94	2.91	2.91	2.96	2.98	2.93	2.94	2.94
17b	LASSO	2.93	2.93	2.92	2.92	2.93	2.93	2.92	2.92	2.93
	SCAD	2.93	2.93	2.92	2.92	2.93	2.93	2.93	2.93	2.93
	MCP	2.93	2.93	2.92	2.92	2.93	2.93	2.93	2.93	2.93
18a	LASSO	3.39	3.11	3.48	3.12	3.41	3.15	3.52	3.22	3.13
	SCAD	3.18	3.07	3.21	3.07	3.15	3.09	3.26	3.11	3.08
	MCP	3.10	3.07	3.10	3.06	3.08	3.08	3.12	3.09	3.09
18b	LASSO	2.97	2.95	2.98	2.95	2.97	2.95	3.00	2.96	2.95
	SCAD	2.96	2.94	2.96	2.94	2.95	2.94	2.98	2.95	2.94
	MCP	2.94	2.94	2.95	2.94	2.94	2.94	2.97	2.94	2.94
19a	LASSO	3.61	3.11	3.71	3.12	3.53	3.15	3.67	3.22	3.13
	SCAD	3.03	3.04	3.02	3.02	3.05	3.05	3.03	3.04	3.04
	MCP	3.04	3.04	3.02	3.02	3.06	3.06	3.03	3.04	3.04
19b	LASSO	2.98	2.95	2.98	2.95	2.97	2.95	2.99	2.96	2.95
	SCAD	2.94	2.94	2.94	2.94	2.94	2.94	2.94	2.94	2.94
	MCP	2.94	2.94	2.94	2.94	2.94	2.94	2.94	2.94	2.94

Case	Method	AIC1	AIC2	BIC1	BIC2	L_q-A1	L_q-A2	L_q-B1	L_q-B2	CV
20a	LASSO	3.62	3.16	3.72	3.17	3.53	3.18	3.67	3.24	3.16
	SCAD	3.02	3.03	3.02	3.02	3.05	3.05	3.03	3.04	3.03
	MCP	3.03	3.04	3.02	3.02	3.06	3.06	3.03	3.04	3.03
20b	LASSO	2.98	2.95	2.98	2.95	2.97	2.95	2.99	2.96	2.95
	SCAD	2.94	2.94	2.94	2.94	2.94	2.94	2.94	2.94	2.94
	MCP	2.94	2.94	2.94	2.94	2.94	2.94	2.94	2.94	2.94
21a	LASSO	-	3.99	-	3.68	-	3.94	-	3.84	3.69
	SCAD	-	5.48	-	3.69	-	4.26	-	4.01	3.67
	MCP	-	5.45	-	3.80	-	4.38	-	4.03	3.69
21b	LASSO	2.96	2.96	2.96	2.96	2.96	2.97	2.96	2.96	2.96
	SCAD	2.96	2.96	2.96	2.96	2.96	2.97	2.96	2.96	2.96
	MCP	2.96	2.97	2.96	2.96	2.97	2.98	2.96	2.96	2.96
22a	LASSO	-	7.05	-	7.09	-	7.10	-	7.87	7.38
	SCAD	-	6.90	-	6.97	-	7.05	-	7.90	24.41
	MCP	-	6.58	-	6.64	-	6.89	-	8.22	25.38
22b	LASSO	3.86	3.17	4.10	3.20	3.89	3.19	4.46	3.36	3.17
	SCAD	3.49	3.11	3.74	3.13	3.42	3.14	4.18	3.23	3.11
	MCP	3.23	3.11	3.34	3.12	3.17	3.13	3.64	3.19	3.11
23a	LASSO	-	229.03	-	229.13	-	229.03	-	229.03	238.30
	SCAD	-	135.98	-	137.91	-	133.22	-	133.22	1709.53
	MCP	-	92.80	-	93.58	-	91.05	-	91.05	1525.86
23b	LASSO	4.63	3.17	5.57	3.20	4.18	3.19	5.56	3.36	3.17
	SCAD	3.08	3.08	3.08	3.08	3.08	3.08	3.08	3.08	3.08
	MCP	3.08	3.08	3.08	3.08	3.09	3.09	3.08	3.08	3.08
24a	LASSO	-	22513	-	22568	-	22441	-	22441	23547
	SCAD	-	13357	-	13490	-	12689	-	12689	168984
	MCP	-	8995	-	9052	-	8584	-	8584	151715
24b	LASSO	4.65	3.26	5.60	3.27	4.18	3.27	5.60	3.37	3.26
	SCAD	3.08	3.08	3.08	3.08	3.08	3.08	3.08	3.08	3.08
	MCP	3.08	3.08	3.08	3.08	3.09	3.09	3.08	3.08	3.08
25a	LASSO	2.93	2.94	2.93	2.93	2.95	2.97	2.94	2.95	2.94
	SCAD	2.94	2.95	2.93	2.93	2.96	2.99	2.94	2.96	2.95
	MCP	2.95	2.96	2.93	2.94	2.98	3.00	2.95	2.96	2.96
25b	LASSO	2.93	2.93	2.93	2.93	2.93	2.93	2.93	2.93	2.93
	SCAD	2.93	2.93	2.93	2.93	2.93	2.93	2.93	2.93	2.93
	MCP	2.93	2.93	2.93	2.93	2.93	2.93	2.93	2.93	2.93
26a	LASSO	3.39	3.13	3.47	3.14	3.42	3.16	3.57	3.21	3.15
	SCAD	3.19	3.11	3.21	3.11	3.17	3.13	3.29	3.15	3.13
	MCP	3.13	3.11	3.13	3.10	3.12	3.12	3.18	3.13	3.13
26b	LASSO	2.98	2.95	2.98	2.95	2.97	2.95	3.01	2.96	2.95
	SCAD	2.96	2.95	2.96	2.95	2.95	2.95	2.99	2.95	2.95
	MCP	2.95	2.95	2.95	2.95	2.95	2.95	2.97	2.95	2.95
27a	LASSO	3.60	3.14	3.71	3.14	3.55	3.16	3.68	3.21	3.14
	SCAD	3.09	3.09	3.08	3.09	3.10	3.10	3.09	3.09	3.10
	MCP	3.09	3.09	3.08	3.09	3.11	3.11	3.09	3.09	3.10
27b	LASSO	2.98	2.95	2.98	2.95	2.97	2.95	2.98	2.96	2.95
	SCAD	2.94	2.94	2.94	2.94	2.94	2.94	2.94	2.94	2.94
	MCP	2.94	2.94	2.94	2.94	2.95	2.95	2.94	2.94	2.95
28a	LASSO	3.65	3.26	6.15	3.26	3.57	3.27	6.11	3.29	3.26
	SCAD	3.09	3.09	3.08	3.08	3.10	3.10	3.09	3.09	3.09
	MCP	3.09	3.09	3.08	3.08	3.10	3.10	3.09	3.09	3.09
28b	LASSO	2.98	2.96	2.98	2.96	2.98	2.96	2.99	2.96	2.96
	SCAD	2.94	2.94	2.94	2.94	2.94	2.94	2.94	2.94	2.94
	MCP	2.94	2.94	2.94	2.94	2.95	2.95	2.94	2.94	2.95
29a	LASSO	-	4.16	-	3.86	-	4.12	-	4.03	3.83
	SCAD	-	5.50	-	3.88	-	4.45	-	4.19	3.83
	MCP	-	5.54	-	3.99	-	4.56	-	4.22	3.87
29b	LASSO	2.98	2.98	2.98	2.98	2.98	2.99	2.98	2.98	2.98
	SCAD	2.98	2.98	2.98	2.98	2.98	2.99	2.98	2.98	2.98
	MCP	2.98	2.99	2.98	2.98	2.99	3.00	2.98	2.98	2.98

Case	Method	AIC1	AIC2	BIC1	BIC2	L_q-A1	L_q-A2	L_q-B1	L_q-B2	CV
30a	LASSO	-	8.09	-	8.12	-	8.12	-	8.58	9.06
	SCAD	-	7.44	-	7.53	-	7.58	-	8.26	35.07
	MCP	-	6.95	-	6.98	-	7.15	-	8.47	38.04
30b	LASSO	3.92	3.19	4.18	3.20	4.06	3.20	4.69	3.30	3.19
	SCAD	3.46	3.16	3.67	3.17	3.48	3.18	4.24	3.25	3.16
	MCP	3.26	3.16	3.35	3.16	3.23	3.17	3.79	3.23	3.16
31a	LASSO	-	331.08	-	331.21	-	331.08	-	331.08	404.68
	SCAD	-	185.22	-	187.28	-	180.93	-	180.93	2867.77
	MCP	-	128.18	-	129.15	-	125.76	-	125.76	2850.95
31b	LASSO	5.50	3.19	6.56	3.20	5.11	3.20	6.59	3.30	3.19
	SCAD	3.49	3.14	3.14	3.14	3.14	3.14	3.14	3.14	3.14
	MCP	3.14	3.14	3.14	3.14	3.14	3.14	3.14	3.14	3.14
32a	LASSO	-	32875	-	32966	-	32653	-	32653	40831
	SCAD	-	18575	-	18777	-	17583	-	17583	281689
	MCP	-	12711	-	12789	-	12104	-	12104	285042
32b	LASSO	16.32	3.40	17.37	3.40	15.92	3.40	17.40	3.42	3.40
	SCAD	28.91	3.14	3.14	3.14	3.14	3.14	3.14	3.14	3.14
	MCP	3.14	3.14	3.14	3.14	3.14	3.14	3.14	3.14	3.14

Table S34: The results on generalization for $q = 5/3$ (the t-error with $\nu = 2$).

Case	Method	AIC1	AIC2	BIC1	BIC2	L_q-A1	L_q-A2	L_q-B1	L_q-B2	CV
1a	LASSO	10.68	10.70	10.68	10.69	11.56	11.76	11.44	11.64	10.72
	SCAD	10.69	10.70	10.68	10.69	11.74	11.82	11.59	11.74	10.72
	MCP	10.69	10.71	10.69	10.69	11.78	11.82	11.67	11.75	10.74
1b	LASSO	25.40	25.42	25.40	25.41	25.65	25.67	25.62	25.65	25.41
	SCAD	25.40	25.42	25.40	25.41	25.67	25.68	25.64	25.67	25.41
	MCP	25.40	25.41	25.40	25.41	25.68	25.68	25.65	25.67	25.41
2a	LASSO	11.21	11.04	11.23	11.06	11.65	11.79	11.57	11.74	11.10
	SCAD	11.16	10.97	11.18	10.99	11.76	11.82	11.66	11.76	11.08
	MCP	11.05	10.97	11.05	10.97	11.80	11.82	11.71	11.76	11.08
2b	LASSO	25.47	25.45	25.47	25.46	25.66	25.68	25.64	25.66	25.46
	SCAD	25.47	25.45	25.47	25.45	25.67	25.68	25.65	25.67	25.46
	MCP	25.45	25.45	25.45	25.45	25.68	25.68	25.66	25.67	25.46
3a	LASSO	11.83	11.32	11.84	11.33	11.65	11.78	11.58	11.73	11.42
	SCAD	11.11	11.02	11.10	11.00	11.76	11.82	11.65	11.75	11.11
	MCP	11.05	11.02	11.04	11.00	11.80	11.82	11.71	11.76	11.09
3b	LASSO	25.54	25.48	25.54	25.49	25.69	25.67	25.64	25.66	25.51
	SCAD	25.46	25.45	25.46	25.45	25.67	25.68	25.65	25.67	25.47
	MCP	25.45	25.45	25.45	25.45	25.68	25.68	25.66	25.67	25.47
4a	LASSO	11.97	11.31	11.99	11.33	11.58	11.59	11.55	11.59	11.39
	SCAD	10.90	10.91	10.89	10.89	11.73	11.74	11.64	11.71	10.93
	MCP	10.90	10.92	10.90	10.90	11.78	11.78	11.70	11.75	10.93
4b	LASSO	25.67	25.52	25.67	25.52	25.65	25.65	25.64	25.65	25.53
	SCAD	25.44	25.44	25.44	25.44	25.67	25.67	25.65	25.66	25.44
	MCP	25.44	25.44	25.43	25.44	25.68	25.68	25.66	25.67	25.44
5a	LASSO	-	14.36	-	14.07	-	21.41	-	20.87	14.15
	SCAD	-	20.45	-	14.07	-	25.19	-	24.75	14.10
	MCP	-	20.98	-	14.09	-	25.24	-	24.74	14.12
5b	LASSO	12.63	12.63	12.63	12.63	13.42	13.79	13.17	13.53	12.63
	SCAD	12.63	12.63	12.63	12.63	13.72	13.86	13.38	13.71	12.63
	MCP	12.63	12.63	12.63	12.63	13.79	13.85	13.52	13.70	12.63
6a	LASSO	-	18.30	-	17.74	-	20.88	-	20.87	17.72
	SCAD	-	21.72	-	19.80	-	24.49	-	24.43	18.85
	MCP	-	21.99	-	20.04	-	24.80	-	24.72	19.12
6b	LASSO	13.44	13.08	13.51	13.13	13.53	13.82	13.39	13.68	13.09
	SCAD	13.43	13.07	13.51	13.12	13.75	13.86	13.54	13.74	13.09
	MCP	13.25	13.08	13.31	13.10	13.81	13.86	13.61	13.74	13.10
7a	LASSO	-	37.57	-	37.57	-	37.88	-	37.88	37.51
	SCAD	-	18.30	-	18.26	-	18.91	-	18.91	18.13
	MCP	-	18.47	-	18.45	-	19.24	-	19.24	18.05
7b	LASSO	15.20	13.25	15.22	13.31	13.54	13.81	13.42	13.68	13.26
	SCAD	13.20	12.90	13.20	12.90	13.75	13.86	13.58	13.73	12.91
	MCP	13.02	12.90	13.02	12.90	13.80	13.86	13.59	13.73	12.91
8a	LASSO	-	1978.49	-	1978.49	-	1978.49	-	1978.49	1978.49
	SCAD	-	16.41	-	16.41	-	16.41	-	16.41	16.42
	MCP	-	16.41	-	16.41	-	16.41	-	16.41	16.42
8b	LASSO	15.28	13.25	15.30	13.31	13.51	13.52	13.42	13.51	13.26
	SCAD	12.85	12.86	12.85	12.85	13.72	13.72	13.48	13.68	12.86
	MCP	12.85	12.86	12.85	12.85	13.79	13.79	13.58	13.72	12.86
9a	LASSO	10.71	10.73	10.71	10.71	11.57	11.76	11.44	11.65	10.75
	SCAD	10.71	10.73	10.71	10.71	11.74	11.82	11.60	11.75	10.74
	MCP	10.72	10.74	10.71	10.71	11.78	11.82	11.68	11.75	10.76
9b	LASSO	25.41	25.42	25.41	25.42	25.65	25.67	25.62	25.65	25.41
	SCAD	25.41	25.42	25.41	25.42	25.67	25.68	25.64	25.67	25.41
	MCP	25.41	25.42	25.41	25.41	25.68	25.68	25.65	25.67	25.41

Case	Method	AIC1	AIC2	BIC1	BIC2	L_q-A1	L_q-A2	L_q-B1	L_q-B2	CV
10a	LASSO	11.62	11.23	11.67	11.25	11.73	11.82	11.72	11.79	11.30
	SCAD	11.53	11.18	11.57	11.21	11.80	11.83	11.74	11.79	11.28
	MCP	11.34	11.19	11.35	11.20	11.82	11.83	11.77	11.80	11.30
10b	LASSO	25.52	25.48	25.52	25.48	25.67	25.68	25.66	25.67	25.48
	SCAD	25.51	25.48	25.52	25.48	25.68	25.68	25.67	25.68	25.49
	MCP	25.49	25.47	25.49	25.48	25.68	25.68	25.67	25.67	25.49
11a	LASSO	13.02	11.64	13.09	11.66	11.74	11.81	11.74	11.78	11.82
	SCAD	11.65	11.31	11.64	11.34	11.80	11.83	11.72	11.78	11.44
	MCP	11.52	11.32	11.51	11.30	11.82	11.83	11.76	11.79	11.48
11b	LASSO	25.66	25.53	25.66	25.54	25.67	25.68	25.66	25.67	25.54
	SCAD	25.51	25.50	25.51	25.49	25.68	25.68	25.66	25.67	25.51
	MCP	25.50	25.50	25.50	25.50	25.68	25.68	25.67	25.67	25.51
12a	LASSO	13.34	11.65	13.41	11.67	11.69	11.69	11.72	11.69	11.72
	SCAD	11.18	11.20	11.18	11.18	11.75	11.75	11.70	11.74	11.20
	MCP	11.19	11.21	11.18	11.18	11.79	11.79	11.75	11.77	11.23
12b	LASSO	25.89	25.56	25.89	25.57	25.66	25.66	25.66	25.66	25.57
	SCAD	25.47	25.47	25.47	25.47	25.67	25.67	25.66	25.67	25.47
	MCP	25.47	25.47	25.47	25.47	25.68	25.68	25.67	25.67	25.47
13a	LASSO	-	14.71	-	14.26	-	21.40	-	20.94	14.33
	SCAD	-	20.98	-	14.26	-	25.18	-	24.78	14.30
	MCP	-	21.37	-	14.29	-	25.25	-	24.79	14.31
13b	LASSO	12.65	12.65	12.65	12.65	13.43	13.79	13.19	13.55	12.66
	SCAD	12.65	12.65	12.65	12.65	13.73	13.86	13.40	13.72	12.66
	MCP	12.65	12.65	12.65	12.65	13.79	13.86	13.53	13.71	12.66
14a	LASSO	-	19.67	-	19.45	-	21.92	-	21.92	19.93
	SCAD	-	25.03	-	21.89	-	25.02	-	25.04	27.87
	MCP	-	25.08	-	22.11	-	25.09	-	25.09	28.74
14b	LASSO	13.89	13.32	14.06	13.37	13.65	13.85	13.63	13.77	13.33
	SCAD	13.85	13.34	14.03	13.38	13.79	13.87	13.70	13.79	13.36
	MCP	13.61	13.35	13.72	13.38	13.83	13.87	13.71	13.78	13.38
15a	LASSO	-	120.93	-	120.94	-	120.93	-	120.93	120.94
	SCAD	-	38.92	-	39.45	-	38.66	-	38.66	136.14
	MCP	-	27.59	-	27.69	-	27.55	-	27.55	68.83
15b	LASSO	18.14	13.57	18.23	13.61	13.67	13.83	13.68	13.78	13.58
	SCAD	13.78	13.18	13.78	13.17	13.78	13.87	13.58	13.77	13.18
	MCP	13.29	13.18	13.29	13.18	13.82	13.87	13.66	13.77	13.18
16a	LASSO	-	10079	-	10088	-	10079	-	10079	10079
	SCAD	-	1715	-	1751	-	1627	-	1627	11198
	MCP	-	696	-	706	-	664	-	664	4802
16b	LASSO	19.35	13.57	19.33	13.61	13.64	13.64	13.68	13.64	13.58
	SCAD	13.11	13.12	13.11	13.11	13.73	13.73	13.58	13.71	13.12
	MCP	13.11	13.12	13.11	13.12	13.79	13.79	13.66	13.75	13.12
17a	LASSO	10.72	10.73	10.72	10.72	11.57	11.76	11.45	11.65	10.76
	SCAD	10.72	10.74	10.72	10.72	11.74	11.81	11.61	11.75	10.76
	MCP	10.73	10.74	10.72	10.72	11.79	11.82	11.69	11.75	10.78
17b	LASSO	25.41	25.42	25.41	25.42	25.65	25.67	25.62	25.65	25.41
	SCAD	25.41	25.42	25.41	25.42	25.67	25.68	25.64	25.67	25.41
	MCP	25.41	25.42	25.41	25.42	25.68	25.68	25.66	25.67	25.42
18a	LASSO	11.76	11.32	11.85	11.34	11.80	11.84	11.81	11.83	11.41
	SCAD	11.67	11.31	11.73	11.33	11.82	11.84	11.80	11.82	11.41
	MCP	11.49	11.33	11.52	11.34	11.83	11.84	11.81	11.82	11.44
18b	LASSO	25.55	25.49	25.56	25.50	25.68	25.68	25.67	25.68	25.50
	SCAD	25.55	25.50	25.56	25.50	25.68	25.68	25.68	25.68	25.50
	MCP	25.52	25.50	25.52	25.50	25.68	25.68	25.68	25.68	25.51
19a	LASSO	13.39	11.74	13.49	11.76	11.83	11.82	11.85	11.82	11.85
	SCAD	11.71	11.51	11.70	11.53	11.82	11.84	11.78	11.81	11.69
	MCP	11.61	11.51	11.59	11.53	11.83	11.84	11.80	11.81	11.76
19b	LASSO	25.74	25.57	25.75	25.57	25.68	25.68	25.68	25.68	25.58
	SCAD	25.56	25.54	25.56	25.54	25.68	25.68	25.67	25.67	25.55
	MCP	25.54	25.54	25.54	25.54	25.68	25.68	25.67	25.68	25.55

Case	Method	AIC1	AIC2	BIC1	BIC2	L_q-A1	L_q-A2	L_q-B1	L_q-B2	CV
20a	LASSO	14.50	11.77	14.61	11.79	11.81	11.78	11.83	11.78	11.81
	SCAD	11.40	11.41	11.39	11.39	11.77	11.77	11.76	11.76	11.41
	MCP	11.41	11.41	11.39	11.39	11.80	11.80	11.79	11.79	11.43
20b	LASSO	25.94	25.58	25.94	25.58	25.67	25.67	25.67	25.67	25.59
	SCAD	25.50	25.50	25.50	25.50	25.68	25.68	25.67	25.67	25.50
	MCP	25.50	25.50	25.50	25.50	25.68	25.68	25.67	25.68	25.50
21a	LASSO	-	15.06	-	14.46	-	21.40	-	21.01	14.53
	SCAD	-	19.49	-	14.47	-	25.17	-	24.80	14.49
	MCP	-	21.77	-	14.53	-	25.25	-	24.79	14.49
21b	LASSO	12.67	12.67	12.67	12.67	13.44	13.79	13.20	13.56	12.68
	SCAD	12.67	12.67	12.67	12.67	13.73	13.86	13.41	13.73	12.68
	MCP	12.67	12.68	12.67	12.67	13.79	13.86	13.54	13.72	12.68
22a	LASSO	-	21.17	-	21.00	-	23.17	-	23.17	22.09
	SCAD	-	24.98	-	22.62	-	25.59	-	25.61	39.41
	MCP	-	25.46	-	23.14	-	25.46	-	25.47	41.60
22b	LASSO	14.10	13.49	14.37	13.52	13.75	13.87	13.86	13.83	13.50
	SCAD	14.06	13.52	14.31	13.55	13.83	13.88	13.86	13.84	13.55
	MCP	13.80	13.53	13.97	13.57	13.85	13.87	13.81	13.83	13.56
23a	LASSO	-	245.48	-	246.07	-	245.48	-	245.48	251.43
	SCAD	-	152.74	-	155.40	-	149.72	-	149.72	1750.79
	MCP	-	109.89	-	110.85	-	107.95	-	107.95	1504.74
23b	LASSO	21.35	13.75	21.74	13.78	13.79	13.85	13.93	13.84	13.76
	SCAD	13.88	13.42	13.88	13.42	13.82	13.87	13.68	13.81	13.42
	MCP	13.52	13.42	13.52	13.41	13.85	13.87	13.74	13.81	13.42
24a	LASSO	-	22552	-	22755	-	22466	-	22466	22979
	SCAD	-	13215	-	13410	-	12520	-	12520	167250
	MCP	-	8963	-	9051	-	8513	-	8513	149655
24b	LASSO	23.77	13.79	24.16	13.81	13.81	13.81	13.93	13.81	13.80
	SCAD	13.37	13.37	13.37	13.37	13.76	13.76	13.68	13.75	13.38
	MCP	13.37	13.38	13.37	13.37	13.81	13.81	13.74	13.79	13.38
25a	LASSO	10.74	10.76	10.74	10.74	11.58	11.77	11.45	11.66	10.78
	SCAD	10.74	10.76	10.74	10.74	11.75	11.82	11.62	11.75	10.78
	MCP	10.75	10.77	10.74	10.74	11.79	11.82	11.69	11.76	10.80
25b	LASSO	25.41	25.42	25.41	25.42	25.65	25.67	25.62	25.66	25.42
	SCAD	25.41	25.42	25.41	25.42	25.67	25.68	25.64	25.67	25.42
	MCP	25.41	25.42	25.41	25.42	25.68	25.68	25.65	25.67	25.42
26a	LASSO	11.80	11.40	11.89	11.41	11.85	11.85	11.89	11.84	11.48
	SCAD	11.67	11.41	11.74	11.42	11.84	11.84	11.84	11.84	11.48
	MCP	11.55	11.42	11.58	11.43	11.84	11.85	11.84	11.84	11.50
26b	LASSO	25.56	25.50	25.57	25.51	25.68	25.68	25.69	25.68	25.51
	SCAD	25.56	25.50	25.57	25.51	25.68	25.68	25.69	25.68	25.52
	MCP	25.53	25.51	25.53	25.51	25.68	25.68	25.68	25.68	25.52
27a	LASSO	13.35	11.78	13.47	11.78	11.86	11.84	11.93	11.84	11.84
	SCAD	11.93	11.67	11.92	11.65	11.84	11.84	11.83	11.83	11.71
	MCP	11.78	11.67	11.77	11.66	11.84	11.84	11.83	11.83	11.71
27b	LASSO	25.78	25.58	25.78	25.58	25.68	25.68	25.69	25.68	25.59
	SCAD	25.60	25.57	25.60	25.57	25.68	25.68	25.68	25.68	25.58
	MCP	25.59	25.57	25.59	25.57	25.68	25.68	25.68	25.68	25.58
28a	LASSO	14.32	11.89	14.43	11.90	11.92	11.92	11.97	11.92	11.92
	SCAD	11.61	11.61	11.60	11.60	11.81	11.81	11.80	11.80	11.63
	MCP	11.62	11.62	11.61	11.61	11.82	11.82	11.82	11.82	11.65
28b	LASSO	26.07	25.65	26.07	25.65	25.68	25.68	25.69	25.68	25.65
	SCAD	25.64	25.59	25.64	25.59	25.68	25.68	25.68	25.68	25.60
	MCP	25.59	25.59	25.59	25.59	25.68	25.68	25.68	25.68	25.60
29a	LASSO	-	15.41	-	14.65	-	21.42	-	21.06	14.71
	SCAD	-	21.32	-	14.66	-	25.17	-	24.82	14.67
	MCP	-	22.01	-	14.74	-	25.26	-	14.82	14.69
29b	LASSO	12.69	12.69	12.69	12.69	13.45	13.80	13.20	13.57	12.69
	SCAD	12.69	12.69	12.69	12.69	13.74	13.86	13.42	13.74	12.69
	MCP	12.69	12.69	12.69	12.69	13.80	13.86	13.55	13.73	12.69

Case	Method	AIC1	AIC2	BIC1	BIC2	L_q-A1	L_q-A2	L_q-B1	L_q-B2	CV
30a	LASSO	-	22.43	-	22.28	-	24.22	-	24.22	24.17
	SCAD	-	25.50	-	25.29	-	26.08	-	26.09	51.72
	MCP	-	25.85	-	24.98	-	25.85	-	25.86	54.17
30b	LASSO	14.14	13.59	14.41	13.61	13.86	13.88	14.06	13.87	13.61
	SCAD	14.06	13.63	14.33	13.65	13.88	13.88	14.01	13.88	13.64
	MCP	13.84	13.64	14.03	13.67	13.88	13.88	13.91	13.88	13.66
31a	LASSO	-	346.97	-	347.79	-	346.97	-	346.97	400.96
	SCAD	-	205.04	-	208.22	-	199.80	-	199.80	2953.96
	MCP	-	147.26	-	148.62	-	144.35	-	144.35	3098.65
31b	LASSO	22.53	13.84	23.21	13.86	13.91	13.87	14.23	13.87	13.85
	SCAD	14.10	13.66	14.13	13.66	13.86	13.88	13.79	13.85	13.66
	MCP	13.79	13.66	13.79	13.66	13.87	13.88	13.82	13.85	13.66
32a	LASSO	-	32921	-	33219	-	32608	-	32608	38121
	SCAD	-	18611	-	18926	-	17490	-	17490	302241
	MCP	-	12751	-	12914	-	12066	-	12066	299829
32b	LASSO	50.71	14.00	54.63	14.00	14.01	14.00	14.23	14.00	14.00
	SCAD	13.63	13.63	13.63	13.63	13.82	13.82	13.79	13.81	13.63
	MCP	13.63	13.63	13.63	13.63	13.84	13.84	13.82	13.83	13.64
